# Supplementary material for: Evaluation of Social Cognition Measures for Japanese Patients with Schizophrenia Using an Expert Panel and Modified Delphi Method
Source: J Pers Med. 2021 Apr 6;11(4):275. doi: 10.3390/jpm11040275 (PMC8067370; doi:10.3390/jpm11040275)
Supplement: Supplementary file 1 [file jpm-11-00275-s001.pdf]

Supplementary Materials

Title:
Evaluation of social cognition measures for Japanese patients with schizophrenia using an expert panel and modified Delphi method

List:

Table S1: Expert Panel Members
Table S2: List of Items Agreed Upon by the Expert Panel
Table S3: Supplementary Data for Candidate Social Cognition Measures

Table S1. Expert Panel Members

Panel Member	Institute	Specialty
Masafumi Mizuno*	Toho University Faculty of Medicine	Schizophrenia
Akiko Kikuchi	National Center of Neurology and Psychiatry	Social psychology
Toshiya Murai	Kyoto University Graduate School of Medicine	Social neuroscience
Shinichi Niwa	Fukushima Medical University	Schizophrenia
Tomiki Sumiyoshi	National Center of Neurology and Psychiatry	Schizophrenia
Tatsuya Koeda	National Center for Child Health and Development	Developmental disorders
Motomu Suga	Teikyo Heisei University/University of Tokyo	Schizophrenia
Daisuke Haga	One More Employment Transition Support Center	Schizophrenia
Jun Tayama	Waseda University Faculty of Human Sciences	Social psychology/neuroscience

*Head of panel.

Table S2. List of Items Agreed Upon by the Expert Panel

Item	Final Proposal
Main participants who would be enrolled	This study will enroll patients with schizophrenia whose symptoms have stabilized following the medication adjustment period in the acute phase and who are undergoing rehabilitation to improve social function.
Objectives	The objectives of this study are (1) to evaluate psychometric properties (including relatedness to scales for evaluating neurocognitive and social function) of existing social cognition tests and (2) determine the suitability of these tests for use in clinical practice in Japan by grading each test and obtaining consensus from panel of experts. Tests that are internationally comparable will be given preference in panel meeting discussions.
Definition of social cognition used in the study	Social cognition refers to cognitive functions that form the basis for interpersonal relations, such as perception of, understanding of, and opposition to another person's intentions, disposition, and behavior.
Main areas of social cognition examined in the study	The main areas of social cognition examined in this study will be the following four areas often seen in research on impairment of social cognitive in schizophrenia: emotional processing, attribution style/bias, social perception, and theory of mind.
Grading for suitability in Japanese patients	The suitability of each test for use in clinical practice in Japan will be graded on a 3-level scale as follows: "suitable for use," "suitable for use under certain conditions," and "use with caution." The suitability of each test will vary depending on the purpose of use, for example, whether it is for clinical research (observational and interventional studies) or for direct clinical use (e.g., for screening or evaluation of rehabilitation outcomes). When the research paper is finalized, grades will be considered for each purpose of use, and also the advantages and precautions for using each test will be described.
Assessment criterion 1: Feasibility and tolerability	A test will be considered feasible and tolerable if testing time for each area of social cognition is less than 15 min and tested patients' subjective rating of inconvenience is low.
Assessment criterion 2: Reliability	A test will be considered "suitable for use" if the correlation coefficient for test-retest reliability is ≥ 0.6.
Assessment criterion 3: Clinical effectiveness	The greatest weight will be given to absence of floor effects in both the first and second evaluations. However, if a test is to be used as an outcome for interventional studies, the greatest weight will be given to the absence of floor/ceiling effects in both the first and second evaluations.
Assessment criterion 4: Validity	The greatest weight will be given to whether results differ markedly between the patients and healthy volunteers and whether the test is strongly correlated with social function. However, whether the addition of a test further increases the ability to predict social function beyond neurocognitive function (i.e., incremental validity) will also be considered.
Assessment criterion 5: International comparability	When two tests are comparably suitable for use in clinical practice in Japan, the six tests recommended in the SCOPE study conducted in the U.S. (BLERT, Hinting, ER-40, Eyes, TASIT, and IBT) will be given preference.

Table S3: Supplementary Data for Candidate Social Cognition Measures

国立研究開発法人日本医療研究開発機構（AMED）

令和 2 年度 障害者対策総合研究開発事業

「社会認知機能に関する新たな検査バッテリーの開発」

専門家パネル資料

候補検査の計量心理学的指標

I.	SCOPE 研究 (2015, 2018)	p.1-9
II.	SCOPE 研究 in Singapore (2019)	p.10-14
III.	候補検査の計量心理学的指標	p.15-108

I.　SCOPE 研究 (2015, 2018)

【研究概要】

米国 SCOPE（Social Cognition Psychometric Evaluation）研究において、一回目（統合失調症 179 名、健常者 104 名）（Pinkham et al, 2015）、二回目（統合失調症 218 名、健常者 154 名）（Pinkham et al, 2018）と観察研究が行われた。

この研究では、今回の AMED 課題で検討する候補検査以外に、RAD、Trust、MiniPONS の３つの検査が組み込まれたが、いずれも十分な計量心理学的指標の結果が出なかった。

【得られた主な結果と今後の課題】

（１）結論

専門家の議論、投票を経て、

1) Emotion processing　2) Theory of mind(ToM)　3) Attributional style/Bias　4) Social

perception

の 4 領域を社会認知機能の core domains として決定した。

二回の観察研究の結果、専門家パネルの議論から、以下の推奨を決定した。

Hinting Task、ER-40、BLERT　→　社会認知を標的とした臨床試験での使用に推奨
Eyes, TASIT, IBT　→やや計量心理学的な指標に課題が残るが、使用は許容される
AIHQ、RAD、Trust、MiniPONS、SAT-MC →　計量心理学的な指標に課題があり、推奨しない

ER-40、BLERT は emotion processing、Eyes、TASIT、Hinting Task は ToM、IBT は Attributional Style/Bias に分類される尺度であり、social perception(社会知覚や原因帰属様式の評価尺度で現在推奨されるものはない（特に社会知覚)。これら領域を評価する尺度が含まれることは重要である。

（２）Eyes, TASIT, IBT の課題

Eyes は、独自に社会機能アウトカムの予測に寄与せず、その評価値は患者（r=.63）とコントロール（r=.47）の両方において WASI の「単語」の点数と強く相関した。過去の研究で、健常者で WASI の「単語」と Eyes の間に.49 の相関が報告されたことから、今回加えた修正がこの関係性を減じることに有効でなかったことが示唆される。

TASIT も社会機能アウトカムと限られた関連性しか示さず、第 3 相と同様に最も所要時間が長いことから一部の臨床試験において非実用的かもしれない。ただし、第 3 相で認められた二つの形式間の齟齬は、実施のカウンターバランスにより減じた様であり、両方の形式を使用する場合はカウンターバランスを実践することを推奨する。

IBT についての懸念事項は、再検査信頼性の低さや、回答時間を制限することによりデータの欠失が増え

ることなどを含む。ただし重要なこととして、IBT は社会機能的能力と実世界の社会機能の両方について、分散を独自に説明することが示された。したがって、IBT は原因帰属様式／バイアスの評価に有用な尺度として有望であり、より詳細な分析と開発（例：症状との関連についての検証、条件効果の影響、など）が成されるのが妥当だろう。

（３）AIHQ の除外の理由

AIHQ の blame score はそうではなかったが、bias score は再検査信頼性が低かった。また社会機能アウトカムとの相関がほとんど認められなった。その理由から、専門家パネルによって、その後の検証から除外された。ただ、原因帰属バイアスの尺度は必要である。近年では、社会認知能力（例：感情の状態を表出する能力）と社会認知バイアス（例：すべての陰性感情を怒りと解釈する傾向）の両方が社会機能不全に関与し、是正の対象とされるべきだ、と考えられ、両者の区別が注目を集めている。これらの尺度の計量心理学的特性が劣っているという結果は、社会認知バイアスの研究の重要性を否定するものではない。原因帰属バイアスの機能的意義は、社会的機能よりもむしろ、攻撃的な行動様式を評価するアウトカムにおいて最も顕著かもしれない。

（４）SAT-MC 除外の理由

社会機能アウトカムとの単相関が認められたものの、社会機能アウトカムに対する予測に独立した寄与はなかった。また、特に SAT-MC の形式 B で、最も大きい床効果を示した。さらに、SAT-MC はおそらくその二つ形式の不均等により、再検査信頼性が劣っていた。

（５）社会知覚の課題について今後の課題

社会知覚の尺度で推奨されたものはなかった。当初の専門家サーベイで社会知覚が重要な領域として示されたことから、既存の尺度を改善し、新たな尺度を開発し続ける必要性が強調される。社会知覚と他の社会認知領域との重複や区別についての更なる明確化は今後役立つかもしれない。

また、Green、Penn ら近年してきたように、社会神経科学の知見を活用することも有用かもしれない。Biological motion では、信号とノイズの弁別力を指標とした場合、再検査信頼性が Pearson’s r が 0.4 程度と低かった。一方、検出閾値といった指標は十分な検討が行われておらず、より優れた結果を残す可能性がある。

1. 実用可能性と忍容性

(Practicality for Administration and Tolerability for Patients)

Table 7. Practicality and Tolerability

	Practicality (Administration Time in Minutes)				Tolerability (Participant Ratings)			
	Patients (n = 95)		Controls (n = 59)		Patients (n = 95)		Controls (n = 59)	
Task	Mean	SD	Mean	SD	Mean	SD	Mean	SD
AIHQ	6.35	2.01	5.82	1.61	5.54	1.30	5.73	1.20
BLERT	7.09	1.50	6.94	0.99	5.14	1.72	5.54	1.58
ER-40	3.21	1.02	2.73	0.73	5.55	1.40	5.59	1.41
Eyes	6.56	3.56	5.45	2.58	5.43	1.59	5.31	1.33
Hinting	6.13	1.89	5.33	1.46	5.38	1.44	5.60	1.50
RAD	15.84	4.45	13.82	3.15	4.74	1.78	4.70	1.53
TASIT	17.92	3.93	17.46	2.12	5.04	1.59	4.83	1.67
Trust	4.46	2.78	3.48	1.29	5.28	1.66	5.19	1.76

Notes: AIHQ, Ambiguous Intentions Hostility Questionnaire; BLERT, Bell Lysaker Emotion Recognition Task; RAD, Relationships Across Domains; TASIT, The Awareness of Social Inferences Test.

RAD、TASIT に関しては、検査所用時間が長く、忍容性もやや低かった。

Table 7. Practicality and Tolerability

	Practicality (Administration Time in Minutes)				Tolerability (Participant Ratings)			
	Patients (n = 218)		Controls (n = 154)		Patients (n = 218)		Controls (n = 154)	
Task	Mean	SD	Mean	SD	Mean	SD	Mean	SD
BLERT	9.86	1.72	9.54	1.77	5.42	1.43	5.51	1.23
ER-40	8.38	1.61	7.82	1.75	5.52	1.44	5.65	1.22
Eyes	6.84	3.38	5.81	2.10	5.36	1.41	5.51	1.24
IBT	5.43	1.00	5.01	0.58	5.08	1.69	5.35	1.28
Hinting	6.85	2.05	6.76	1.44	5.35	1.54	5.75	1.06
MiniPONS	12.17	2.37	11.08	1.76	4.65	1.79	4.76	1.58
SAT-MC	10.26	1.75	9.58	1.22	5.22	1.58	5.55	1.29
TASIT	18.62	1.73	17.94	1.48	5.07	1.55	5.38	1.18

TASIT、MiniPONS は所要時間が長く、被験者にとって負担が大きく、好まれなかった。

２．信頼性

(Test-Retest, Internal Consistency, Inter-rater Reliability as applicable)

再検査信頼性に関しては、患者群で Pearson’ｓ r ≥.6 を満たしたのは、BLERT、ER-40、Eyes、Hinting、TASIT であった。

AIHQ の bias score は再検査信頼性が低かったが、blame score はそうではなかった。

Table 2. Test-Retest Reliability and Internal Consistency

Task	Test-Retest Reliability (Person *r*) Patients (*n* = 171)	Controls (*n* = 98)	Internal Consistency (Cronbach's Alpha) Patients (*n* = 179)	Controls (*n* = 104)
AIHQ				
Hostility bias (HB)	.516	.572	.859	.846
Aggression bias (AB)	.572	.700	.422	.467
Blame Score (BS)	.738	.756	.491	.338
BLERT	.699	.680	.737	.626
ER-40	.753	.753	.808	.645
Eyes	.753	.761	.735	.673
Hinting	.639	.424	.729	.563
RAD	.751	.756	.717	.700
TASIT	.600	.544	.807	.757
Trust	.737	.597	.960	.900

Notes: AIHQ, Ambiguous Intentions Hostility Questionnaire; BLERT, Bell Lysaker Emotion Recognition Task; RAD, Relationships Across Domains; TASIT, The Awareness of Social Inferences Test.

Table 2. Test-Retest Reliability and Internal Consistency

Task	Test-Retest Reliability (Person *r*) Patients (*n* = 208)	Controls (*n* = 148)	Internal Consistency (Cronbach's Alpha) Patients (*n* = 218)	Controls (*n* = 154)
BLERT	.809	.622	.778	.570
ER-40	.710	.679	.754	.555
Eyes	.806	.716	.750	.640
IBT	.587	.511	.538	.503
Hinting	.695	.509	.681	.635
MiniPONS	.721	.663	.712	.656
SAT-MC	.573	.554	.786	.735
TASIT	.636	.534	.807	.825
BLERT CR	.613	.701	.962	.932
BLERT RT	.658	.660	.939	.951
ER-40 CR	.625	.796	.973	.962
ER-40 RT	.662	.629	.915	.914
TASIT RT	.687	.559	.920	.881

Note: Due to the time limit on responding, many participants had missed trials on the IBT. Estimates of internal consistency for this task are therefore based on much smaller samples of participants (26 patients and 38 controls) who responded to all items.
Abbreviations: BLERT, Bell Lysaker Emotion Recognition Task; ER-40, Penn Emotion Recognition Test; IBT, Intentionality Bias Task; MiniPONS, Mini Profile of Nonverbal Sensitivity; SAT-MC, Social Attribution Test-Multiple Choice; TASIT, The Awareness of Social Inferences Test; CR, confidence ratings; RT, response time

再検査信頼性に関しては、SAT-MC、IBT 以外の検査は、患者群で Pearson’ｓ r ≥.6 を満たした。SAT-MC はおそらく二つの形式の不均等により、再検査信頼性が劣っていた。

３．有用性

(Floor and Ceiling Effects, Normality of Distributions)
(Utility as a Repeated Measure)
※この項目では、自然経過では不変で介入によってより大きく動く尺度が良いと考える。

Table 3. Utility as a Repeated Measure

Task	T_1 Mean	T_1 SD	T_2 Mean	T_2 SD	T_2–T_1 Difference Mean	T_2–T_1 Difference SD	Number at Floor/Ceiling T_1	Number at Floor/Ceiling T_2	t	P value	Cohen's d_z
Patients (n = 171)											
AIHQ-HB	2.38	0.61	2.21	0.64	−0.17	0.62	—	—	−3.57	<.001	0.27
AIHQ-AB	1.88	0.39	1.95	0.44	0.06	0.39	—	—	2.05	.04	0.16
AIHQ-BS	8.76	2.85	8.42	3.06	−0.34	2.15	—	—	−2.06	.04	0.16
BLERT	13.24	3.82	13.91	3.99	0.67	3.04	1/0	0/4	2.87	.005	0.22
ER-40	29.69	5.37	30.42	4.95	0.73	3.65	1/0	0/0	2.62	.01	0.20
Eyes	20.22	5.52	20.66	5.85	0.44	4.00	5/0	4/0	1.43	.15	0.11
Hinting	13.65	3.80	14.25	3.68	0.60	3.18	0/2	0/2	2.46	.02	0.19
RAD	24.79	5.79	25.86	5.70	1.07	4.06	77/0	56/0	3.40	.001	0.26
TASIT	44.55	7.55	42.92	6.36	−1.63	6.31	12/0	9/0	−3.37	.001	0.26
Trust	−0.12	1.13	−0.002	0.91	0.12	0.77	—	—	2.01	.05	0.15
Controls (n = 98)											
AIHQ-HB	2.00	0.60	1.78	0.53	−0.22	0.53	—	—	-4.19	<.001	0.42
AIHQ-AB	1.83	0.26	1.82	0.31	−0.01	0.22	—	—	−0.28	.78	0.05
AIHQ-BS	7.08	2.30	6.34	2.41	−0.73	1.65	—	—	−4.41	<.001	0.44
BLERT	15.74	2.89	16.12	2.96	0.38	2.34	0/2	0/1	1.59	.11	0.16
ER-40	32.61	3.53	33.13	3.41	0.52	2.44	0/0	0/0	2.11	.04	0.21
Eyes	23.50	4.71	23.55	5.34	0.05	3.52	0/0	2/0	0.14	.89	0.01
Hinting	16.85	2.01	17.45	1.50	0.59	1.93	0/6	0/7	3.02	.003	0.31
RAD	29.87	5.21	30.45	5.61	0.58	3.80	12/0	9/0	1.52	.13	0.15
TASIT	51.44	5.68	48.21	6.58	−3.22	5.91	0/0	0/0	−5.40	<.001	0.54
Trust	0.18	0.60	0.24	0.58	0.06	0.53	—	—	1.11	.27	0.11

RAD は床効果が著しく、学習効果（Cohen’s d）も高かった。
TASIT も床効果が認められ、学習効果は高かった。

Table 3. Utility as a Repeated Measure

Task	T_1/Version A Mean	T_1/Version A SD	T_2/Version B Mean	T_2/Version B SD	T_2-T_1 Difference Mean	T_2-T_1 Difference SD	Number at Floor/Ceiling T_1	Number at Floor/Ceiling T_2	t	P Value	Cohen's d_z
	Patients (n = 208)										
BLERT	13.96	3.96	14.93	3.80	.97	2.40	0/3	2/5	5.82	<.001	.40
ER-40	31.17	4.20	31.34	4.30	.17	3.24	0/0	0/0	.78	.439	.05
Eyes	21.20	5.52	20.76	5.68	−.44	3.49	3/0	9/0	−1.81	.072	.13
IBT	.44	.18	.40	.18	−.04	.16	—	—	−3.55	<.001	.26
Hinting	13.43	3.70	13.89	4.10	.47	3.07	1/2	1/4	2.20	.029	.15
MiniPONS	42.95	6.37	43.44	6.80	.49	4.94	14/0	12/0	1.42	.158	.10
SAT-MC	11.89	4.01	10.05	4.12	−1.84	3.76	9/3	24/2	−7.00	<.001	.49
TASIT	44.56	7.43	43.73	6.80	−.83	6.10	9/0	10/0	−1.96	.052	.14
BLERT CR	80.66	16.66	82.03	15.08	1.37	14.03	0/22	0/20	1.41	.161	.10
BLERT RT (s)	16.02	3.74	15.03	3.83	-.99	3.13	—	—	−4.55	<.001	.32
ER-40 CR	83.88	13.25	83.76	14.18	−.11	11.90	0/25	0/19	−.14	.891	.01
ER-40 RT (s)	3.87	1.11	3.45	1.09	−.42	.91	—	—	−6.55	<.001	.46
TASIT RT (s)	55.79	4.52	57.65	4.57	1.86	3.60	—	—	7.11	<.001	.52
	Controls (n = 148)										
BLERT	15.87	2.72	16.58	2.85	.71	2.43	0/3	0/7	3.56	.001	.29
ER-40	32.86	3.21	33.20	3.50	.33	2.70	0/0	0/0	1.49	.138	.12
Eyes	24.69	4.34	24.40	4.79	−.29	3.46	0/0	0/0	−1.02	.309	.08
IBT	.40	.15	.37	.16	−.03	.15	—	—	−2.14	.034	.18
Hinting	15.44	2.65	15.93	2.81	.49	2.71	0/8	0/12	2.16	.033	.18
MiniPONS	46.58	5.59	46.84	5.89	.257	4.72	3/0	2/0	0.66	.509	.05
SAT-MC	14.21	3.30	13.14	3.96	−1.07	3.48	1/2	5/7	−3.75	<.001	.31
TASIT	50.46	6.83	49.72	7.12	−.74	6.74	1/0	2/0	−1.32	.189	.11
BLERT CR	85.20	10.55	86.65	10.68	1.45	8.21	0/5	0/9	2.15	.033	.18
BLERT RT (s)	15.59	3.49	13.79	3.41	−1.80	2.85	—	—	−7.69	<.001	.63
ER-40 CR	84.92	10.69	85.20	10.71	.29	6.83	0/4	0/6	0.51	.610	.04
ER-40 RT (s)	3.56	1.03	3.14	0.87	−.42	0.83	—	—	−6.02	<.001	.50
TASIT RT (s)	53.83	3.90	55.37	3.47	1.54	3.48	—	—	5.24	<.001	.44

SAT-MC の形式 B が、最も大きい床効果を示した。

４．妥当性

(Relationship to social cognitive measures relative to other abilities and constructs)

※この項目では、他の社会認知機能との関連が強い尺度が良いと考える。

この研究では、社会認知機能検査間の関連は報告していない。

健常者との比較では、Trust が健常者との差が小さかった。

Table 8. Group Differences on Social Cognitive Measures

	Patients (n = 179)		Controls (n = 104)				
Task	Mean	SD	Mean	SD	t	P	Cohen's d
AIHQ-HB	2.38	0.60	1.99	0.60	5.29	<.001	.65
AIHQ-AB	1.89	0.38	1.83	0.26	1.46	.147	.18
AIHQ-BS	8.74	2.81	7.02	2.31	5.29	<.001	.67
BLERT	13.17	3.88	15.75	2.88	−6.38	<.001	.76
ER-40	29.55	5.40	32.80	3.54	−6.10	<.001	.71
Eyes	20.15	5.46	23.55	4.62	−5.58	<.001	.67
Hinting	13.59	3.87	16.82	2.05	−9.14	<.001	1.04
RAD	24.76	5.76	29.82	5.16	−7.37	<.001	.93
TASIT	44.43	7.64	51.48	5.62	−8.89	<.001	1.05
Trust	−0.09	1.14	0.16	0.62	−2.33	.02	.27

Table 8. Group Differences on Social Cognitive Measures

	Patients (n = 218)		Controls (n = 154)				
Task	Mean	SD	Mean	SD	t	P	Cohen's d
BLERT	13.93	4.02	15.92	2.70	5.70	<.001	.58
ER-40	31.12	4.28	32.94	3.19	4.69	<.001	.48
Eyes	21.28	5.49	24.79	4.33	6.88	<.001	.71
IBT	.44	.18	.40	.15	−2.09	.037	.24
Hinting	13.36	3.71	15.38	2.68	6.05	<.001	.62
MiniPONS	42.88	6.47	46.69	5.52	6.10	<.001	.63
SAT-MC	11.91	4.00	14.24	3.28	6.09	<.001	.64
TASIT	44.56	7.44	50.57	6.80	7.81	<.001	.84
BLERT CR	81.06	16.66	85.58	10.56	3.20	.001	.32
BLERT RT (s)	16.04	3.69	15.45	3.56	−1.54	.124	.16
ER-40 CR	84.08	13.48	85.05	10.72	.776	.438	.08
ER-40 RT (s)	3.89	1.11	3.55	1.04	−2.99	.003	.32
TASIT RT (s)	55.91	4.56	53.91	3.98	−4.24	<.001	.47

健常者との比較では、IBT が健常者との差が小さかった。

(Correlations with Real-World Social Outcomes)

BLERT と Hinting task は検査の追加によって神経認知機能よりもさらに社会機能の予測力を増す、有意な増分妥当性を示した（Table6）。

一方、AIHQ はほとんど相関が認められなかった。

Table 4. Correlations between Social Cognitive Tasks and Functional Outcome Measures in Patients

	UPSA Total	SSPA Average	SLOF Total
Social cognitive			
AIHQ-HB	−.071	.063	−.058
AIHQ-AB	.041	.078	−.071
AIHQ-BS	−.005	.094	−.137
BLERT	.317***	.261***	.310***
ER-40	.360***	.240***	.046
Eyes	.425***	.300***	.127
Hinting	.462***	.394***	.197**
RAD	.439***	.243**	.202**
TASIT	.437***	.310**	.304***
Trust	.052	−.030	.043
Neurocognitive			
Trails A	−.270***	−.103	−.237**
Symbol coding	.264***	.301***	.263***
HVLT-R	.421***	.358***	.174*
Letter number span	.544***	.317***	.255**
Animal naming	.174*	.168*	.078

Table 5. Regression Models Demonstrating the Overall Contribution of the Social Cognitive Tasks to Outcomes

	R^2	Adjusted R^2	F	P	b^*	t	P	sr^2
UPSA total	.332	.308	13.92	<.001				
BLERT					−.08	−.88	.382	.003
ER-40					.11	1.28	.20	.007
Eyes					.09	.99	.32	.004
Hinting					.29	3.91	<.001	.06
RAD					.19	2.29	.02	.02
TASIT					.13	1.47	.15	.008
SSPA average	.186	.156	6.35	<.001				
BLERT					.04	.39	.70	.000
ER-40					.05	.48	.63	.001
Eyes					.08	.77	.44	.002
BLERT					.21	2.35	.02	.03
Hinting					.04	.52	.60	.001
RAD					−.02	−.26	.79	.000
TASIT					.19	1.94	.05	.02
SLOF-HQ	.190	.159	6.13	<.001				
AIHQ-BS					−.12	−1.52	.13	.01
BLERT					.38	3.83	<.001	.09
Hinting					−.01	−.08	.94	.000
RAD					−.05	−.47	.64	.001
TASIT					.08	.72	.47	.003

Notes: SLOF-HQ indicates ratings from high quality informants (ie, professionals with mental health experience). AIHQ, Ambiguous Intentions Hostility Questionnaire; BLERT, Bell Lysaker Emotion Recognition Task; RAD, Relationships Across Domains; TASIT, The Awareness of Social Inferences Test. b^* indicates standardized coefficients.

Table 6. Final Regression Models Accounting for Additional Variance in Outcome beyond Neurocognitive Performance

	UPSA-B		SSPA		SLOF		SLOF-HQ	
	b^*	sr^2	b^*	sr^2	b^*	sr^2	b^*	sr^2
Block 1—Neurocognition								
Trails A	−.157*	.016*	—	—	−.109	.008	−.060	.002
Symbol coding	−.106	.006	.136,	.013	.069	.003	.007	.000
HVLT-R	.087	.004	.191*	.021*	−.028	.000	.005	.000
Letter number span	.310**	.046**	.071	.003	.087	.004	.072	.003
Animal Naming	−.050	.002	−.008	.000	—	—	—	—
Block 2—Social cognition								
AIHQ-BS	—	—	—	—	—	—	−.094	.008
BLERT	−.116	.007	.005	.000	.154	.015	.353**	.076**
ER-40	.094	.005	−.022	.000	—	—	—	—
Eyes	.043	.000	.052	.001	—	—	—	—
Hinting	.242**	.041**	.258**	.047**	.038	.001	−.020	.000
RAD	.082	.003	−.095	.004	−.060	.002	−.071	.003
TASIT	.090	.004	.059	.002	.158	.013	.065	.002
Overall model								
Adjusted R^2	.372***		.187***		.112**		.133**	
R^2 change	.082**		.068*		.047		.113**	

Notes: SLOF-HQ indicates ratings from high quality informants (ie, professionals with mental health experience). AIHQ, Ambiguous Intentions Hostility Questionnaire; BLERT, Bell Lysaker Emotion Recognition Task; RAD, Relationships Across Domains; TASIT, The Awareness of Social Inferences Test. b^* indicates standardized coefficients.
$^*P < .05$, $^{**}P < .01$, $^{***}P < .001$.

Table 4. Correlations Between Social Cognitive Tasks and Functional Outcome Measures in Patients

	UPSA Total (*n* = 208)	SSPA Average (*n* = 208)	SLOF Community Informant (*n* = 135)	SLOF-HQ Community Informant (*n* = 53)
Social cognitive				
BLERT	.368***	.415***	.208*	.062
ER-40	.361***	.410***	.174*	.088
Eyes	.381***	.277***	.154	.086
IBT	−.189**	−.137	−.191*	−.004
Hinting	.404***	.437***	.192*	.345*
MiniPONS	.391***	.379***	.169*	.092
SAT-MC	.265***	.329***	−.004	−.028
TASIT	.362***	.380***	.106	−.016
BLERT CR	−.080	−.030	.060	−.412**
BLERT RT (sec)	−.029	−.176*	−.102	.062
ER-40 CR	−.181**	−.090	−.030	−.371**
ER-40 RT (sec)	−.110	−.292***	.043	−.167
TASIT RT (sec)	−.018	−.105	.089	−.046
Neurocognitive				
TrailsA	−.291***	−.215**	.022	−.100
Symbol Coding	.388***	.290***	.095	.255
HVLT-R	.394***	.337***	.198*	.328*
Letter-Number Span	.423***	.322***	.217*	.096
Animal Naming	.236**	.195**	.042	.026

Note: SLOF informant ratings were available for only a subset of the patient sample. SLOF-HQ indicates ratings from high quality informants (ie, professionals with mental health experience).
**P* < .05, ***P* < .01, ****P* < .001.

Table 5. Regression Models Demonstrating the Overall Contribution of the Social Cognitive Tasks to Outcomes

	R^2	Adjusted R^2	*F*	*P*	*b**	*t*	*P*	sr^2
UPSA total	.28	.25	8.16	<.001				
BLERT					.01	.05	.96	.000
ER-40					.12	1.34	.18	.007
Eyes					.05	.56	.58	.001
IBT					−.15	−2.34	.020	.021
Hinting					.26	3.87	<.001	.057
MiniPONS					.14	1.66	.10	.010
SAT-MC					.02	.27	.79	.000
TASIT					.09	1.06	.29	.004
ER-40 CR					−.11	−1.64	.10	.010
SSPA average	.34	.31	10.74	<.001				
BLERT					.08	.87	.39	.003
ER-40					.16	1.93	.06	.017
Eyes					−.07	−.74	.46	.002
Hinting					.26	4.04	<.001	.057
MiniPONS					.09	1.11	.27	.004
SAT-MC					.03	.43	.67	.001
TASIT					.11	1.38	.17	.007
BLERT RT					.01	.10	.92	.000
ER-40 RT					−.20	−2.93	.004	.030
SLOF total	.095	.059	2.63	.03				
BLERT					.10	.89	.38	.006
ER-40					.04	.40	.69	.001
IBT					−.18	−2.06	.04	.031
Hinting					.13	1.45	.15	.015
MiniPONS					.06	.58	.56	.002
SLOF-HQ total	.221	.173	4.63	.006				
Hinting					.23	1.63	.11	.042
BLERT CR					−.31	−1.61	.11	.041
ER-40 CR					−.04	−.23	.82	.001

Note: SLOF-HQ indicates ratings from high quality informants (ie, professionals with mental health experience).

Table 6. Final Regression Models Accounting for Additional Variance in Outcome beyond Neurocognitive Performance

	UPSA-B (n = 196)		SSPA (n = 193)		SLOF (n = 128)		SLOF-HQ (n = 50)	
	b^*	sr^2	b^*	sr^2	b^*	sr^2	b^*	sr^2
Block 1—Neurocognition								
Trails A	−.06	.002	.05	.002	—	—	—	—
Symbol Coding	.13	.008	.07	.002	—	—	—	—
HVLT-R	.16*	.016*	.09	.004	.07	.003	.23	.035
Letter-Number Span	.12	.008	.05	.002	.09	.005	—	—
Animal Naming	−.06	.003	−.05	.002	—	—	—	—
Block 2— Social Cognition								
BLERT	−.08	.002	.07	.002	.03	.000	—	—
ER-40	.13	.009	.17*	.014*	.06	.002	—	—
Eyes	.004	.000	−.11	.004	—	—	—	—
IBT	−.12	.014	—		−.19*	.035*	—	—
Hinting	.22**	.043**	.25***	.050***	.11	.009	.18	.022
MiniPONS	.11	.006	.07	.003	.05	.001	—	—
SAT-MC	.03	.000	.03	.000	—	—	—	—
TASIT	.003	.000	.08	.003	—	—	—	—
BLERT CR	—		—	—	—	—	−.38	.062
BLERT RT	—		−.001	.000	—	—	—	—
ER-40 CR	−.12	.013	—	—	—	—	.10	.004
ER-40 RT	—		−.19*	.024*	—	—	—	—
Overall Model								
Adjusted R^2	.295***		.287***		.06*		.188**	
R^2 Change	.11**		.18***		.06		.146*	

Note: SLOF-HQ indicates ratings from high-quality informants (ie, professionals with mental health experience).
*P < .05, **P < .01, ***P < .001.

Eyes は、独自に社会機能アウトカムの予測に寄与せず、その評価値は患者（r=.63）とコントロール（r=.47）の両方において WASI の「単語」の点数と強く相関した。過去の研究で、健常者で WASI の「単語」と Eyes の間に.49 の相関が報告されたことから、今回加えた修正がこの関係性を減じることに有効でなかったことが示唆される。

II.　SCOPE 研究 in Singapore (2019)

【研究概要】

米国 SCOPE（Social Cognition Psychometric Evaluation）研究の文化差を越えた追試を目的に、シンガポールで研究（2019）（英語話者である、統合失調症 116 名、健常者 73 名）（Lim et al, 2019）が行われた。

この研究では両群に社会認知機能検査を行っただけで、神経認知機能、社会機能を評価していない。

この研究では、米国 SCOPE 研究で推奨された 6 つ（Hinting、BLERT、TASIT、ER-40、Eyes、IBT）のうち４つの検査（Hinting、BLERT、TASIT、ER-40）と、それ以外の AIHQ、MiniPONS、RAD、IPSAQ、MSCEIT の 5 つの検査を社会認知機能検査として用いている。AIHQ の一部で米国 SCOPE 研究と異なる結果が出たが、残り 4 つの検査は計量心理学的指標で劣った結果であった。

【得られた主な結果と今後の課題】

（１）BLERT と ER-40 は、忍容性、信頼性、有用性が高かった。

選ばれた尺度のうち、BLERT と ER-40 が最も好ましい結果を示した。いずれも床／天井効果をほとんど認めず、忍容性が高く、所要時間が短かった。これらは臨床現場での実装において、重要な条件である。いずれも emotional processing を指標化する尺度である。

（２）TASIT の課題

TASIT-3 は十分な再検査信頼性と内的一貫性を示した。一方で、患者群ではわずかに床効果が観察されたが、コントロール群では認められなかった。

（３）AIHQ は米国の研究より、再検査が少し良かった。

SCOPE 研究（Pinkham et al., 2016）では、AIHQ-BS のみが 0.6 を超える再検査信頼性（相関係数）を示し、社会機能との関連が認められたのに対し、本研究では AIHQ-HB、AIHQ-BS が 0.6 を超える再検査信頼性（相関係数）を示した。この結果は、SCOPE 研究では曖昧なシナリオのみを使用した（Pinkham et al., 2016）のに対し、本研究では偶然や故意のものを含む AIHQ の全シナリオを使用したことによって説明できるかもしれない。SCOPE 研究の第 3 相の結果により AIHQ の使用には注意を要するとされた（Pinkham et al., 2016）が、その発展として、パラノイアや敵意の症状への関与について付加的な情報を提供することや、対人関係の困難との関連において有用かもしれない（Buck et al., 2016b）。より直近では、Buck et al.（2017）は偶然のシナリオの追加により、曖昧なシナリオのみの場合と比べて、AIHQ-BS の自己評価領域が社会機能的能力により強く関与し、AIHQ-HB の評価者スコア領域が role functioning に僅かながら関連するという結果を得た。このことは、AIHQ の曖昧なシナリオと故意のシナリオの両方を含めることが、社会機能的なアウトカムとの関連を改善させることを示唆する（Pinkham et al., 2016）。

（４）Hinting は米国 SCOPE に比べて悪い結果

Hinting task は過去の研究 (Davidson et al., 2018; Pinkham et al., 2018, 2016) と比べて再検査信頼性が低かった(相関係数が 0.6 を下回った)。この結果を説明しうる要因として、Hinting task で被験者の推論能力を調べる際に使用する社会的場面の文章題が、文化的な差異の影響を受けやすい可能性が考えられる。今後、文化的に適切な文章題の使用が尺度の計量心理学的な特性を向上させるか否かという点に関しては特に、さらなる検証が推奨される。

（５）社会知覚の課題について今後の課題

社会知覚の課題である MiniPONS、RAD が今回の検討に加えられたが、米国での検討同様、不十分な計量心理学的特性を示した。十分な計量心理学的特性を示す、社会知覚を評価する検査が求められる。

1. 実用可能性と忍容性

(Practicality for Administration and Tolerability for Patients)

Table 4
Practicality and tolerability.

	Practicality (administration time in minutes)				Tolerability			
	Cases		Controls		Cases		Controls	
	T_1	T_2	T_1	T_2	T_1	T_2	T_1	T_2
Hinting	7.44 (2.03)	6.72 (1.92)**	6.24 (1.42)	5.59 (1.08)**	5.42 (1.43)	5.29 (1.44)	5.66 (1.05)	5.60 (1.16)
BLERT	7.25 (1.02)	7.06 (1.03)	7.13 (1.08)	6.93 (0.82)	5.30 (1.35)	5.33 (1.28)	5.41 (1.14)	5.29 (1.19)
MiniPONS	13.57 (1.47)	14.20 (6.67)	13.29 (0.76)	13.03 (0.61)*	4.83 (1.59)	4.96 (1.47)	4.76 (1.56)	5.01 (1.49)
RAD	17.28 (6.61)	13.85 (4.59)**	14.80 (5.05)	11.26 (4.12)**	4.79 (1.59)	4.91 (1.52)	4.90 (1.29)	5.23 (1.21)
AIHQ	18.90 (7.52)	16.87 (6.13)**	16.46 (6.96)	12.37 (4.58)**	5.21 (1.34)	5.10 (1.51)	5.60 (0.95)	5.44 (1.10)
IPSAQ	18.24 (7.78)	18.01 (8.58)	17.07 (7.34)	14.30 (5.75)**	5.17 (1.50)	5.07 (1.56)	4.99 (1.35)	5.21 (1.30)
MSCEIT[a]	37.21 (15.39)	33.14 (16.10)**	28.16 (10.12)	24.43 (9.58)**	5.17 (1.47)	5.06 (1.35)	5.13 (1.35)	5.26 (1.08)
TASIT[a]	48.84 (6.68)	46.55 (4.61)**	44.7 (4.47)	42.89 (3.18)**	4.79 (1.70)	4.68 (1.58)	5.01 (1.31)	4.90 (1.49)
ER40	3.82 (2.02)	3.53 (1.01)	2.94 (0.83)	2.74 (1.02)**	5.58 (1.35)	5.55 (1.39)	5.87 (0.98)	5.99 (0.96)

Note. All values in cells represent mean (SD). $**p < 0.01$ and $*p < 0.05$ represents significant difference in task administration time across study visits. BLERT = Bell Lysaker Emotion Recognition Task; MiniPONS = Mini Profile of Nonverbal Sensitivity; RAD = Relationships Across Domains; AIHQ = Ambiguous Intentions and Hostility Questionnaire; IPSAQ = Internal Personal and Situational Attributions Questionnaire; MSCEIT = Mayer-Salovey-Caruso Emotional Intelligence; TASIT = The Awareness of Social Inference Test; ER40 = Penn Emotion Recognition Task.

[a] Outcome measured includes all branches of task.

10 分以内で検査が終了したのは、Hinting、BLERT、ER-40 のみであった。

２．信頼性

(Test-Retest, Internal Consistency, Inter-rater Reliability as applicable)

Table 2
Test-retest reliability and internal consistency.

	Cases				Controls			
	Spearman's rho	ICC	T_1 Cronbach's α	T_2 Cronbach's α	Spearman's rho	ICC	T_1 Cronbach's α	T_2 Cronbach's α
Hinting	0.573**	0.608**	0.640	0.616	0.505**	0.551**	0.479	0.294
BLERT	0.727**	0.727**	0.701	0.747	0.513**	0.525**	0.544	0.548
MiniPONS	0.557**	0.572**	0.657	0.652	0.618**	0.628**	0.562	0.527
RAD	0.796**	0.790**	0.775	0.801	0.776**	0.790**	0.727	0.780
AIHQ-HB	0.662**	0.694**	0.626	0.688	0.615**	0.644**	0.541	0.428
AIHQ-AB	0.514**	0.486**	0.683	0.667	0.747**	0.739**	0.545	0.752
AIHQ-BS	0.632**	0.634**	0.936	0.952	0.780**	0.786**	0.933	0.947
IPSAQ-EB	0.613**	0.606**	0.834	0.850	0.468**	0.516**	0.764	0.774
IPSAQ-PB	0.227*	0.219**	0.845	0.818	0.558**	0.561**	0.821	0.814
MSCEIT-PE	0.671**	0.702**	0.919	0.938	0.589**	0.568**	0.893	0.940
MSCEIT-FE	0.688**	0.659**	0.801	0.844	0.548**	0.564**	0.756	0.821
MSCEIT-UE	0.778**	0.778**	0.822	0.827	0.827**	0.795**	0.718	0.744
MSCEIT-ME	0.716**	0.731**	0.813	0.828	0.561**	0.570**	0.725	0.764
TASIT-1	0.383**	0.432**	0.706	0.707	0.340**	0.377**	0.514	0.680
TASIT-2	0.481**	0.461**	0.823	0.778	0.438**	0.464**	0.798	0.863
TASIT-3	0.657**	0.635**	0.755	0.726	0.525**	0.547**	0.802	0.794
ER40	0.585**	0.606**	0.656	0.752	0.575**	0.589**	0.508	0.602

Note. BLERT = Bell Lysaker Emotion Recognition Task; MiniPONS = Mini Profile of Nonverbal Sensitivity; RAD = Relationships Across Domains; AIHQ-HB = Ambiguous Intentions and Hostility Questionnaire – Hostility Bias; AIHQ-AB = Ambiguous Intentions and Hostility Questionnaire – Aggression Bias; AIHQ-BS = Ambiguous Intentions and Hostility Questionnaire – Blame Score; IPSAQ-EB = Internal Personal and Situational Attributions Questionnaire – Externalizing Bias; IPSAQ-PB = Internal Personal and Situational Attributions Questionnaire – Personalizing Bias; MSCEIT-PE = Mayer-Salovey-Caruso Emotional Intelligence Test – Perceiving Emotions; MSCEIT-FE = Mayer-Salovey-Caruso Emotional Intelligence Test – Facilitating Emotions; MSCEIT-UE = Mayer-Salovey-Caruso Emotional Intelligence Test – Understanding Emotions; MSCEIT-ME = Mayer-Salovey-Caruso Emotional Intelligence Test – Managing Emotions; TASIT-1 = The Awareness of Social Inference Test – Branch 1; TASIT-2 = The Awareness of Social Inference Test – Branch 2; TASIT-3 = The Awareness of Social Inference Test – Branch 3; ER40 = Penn Emotion Recognition Task.

** $p < 0.01$.

* $p < 0.05$.

患者群で 0.6 を超えたのは、BLERT、RAD、AIHQ-HB、AIHQ-BS、IPSAQ-EB、MSCEIT、TASIT-3 であった。

３．有用性

(Floor and Ceiling Effects, Normality of Distributions)

(Utility as a Repeated Measure)

※この項目では、自然経過では不変で介入によってより大きく動く尺度が良いと考える。

Table 3
Utility as a repeated measure.

	N	T_1		T_2		*P*	Cohen's d	Floor/ceiling effect	
		Mean	SD	Mean	SD			T1	T2
Cases (*n* = 108)									
Hinting	108	14.31	3.34	14.98	3.24	0.03	0.20	0/3	0/5
BLERT	108	14.26	3.34	15.44	3.37	< 0.01	0.35	1/0	1/3
MiniPONS	107	43.42	5.73	44.36	5.87	0.14	0.16	3/0	4/0
RAD	108	27.92	6.12	28.01	6.38	0.80	0.01	21/0	23/0
AIHQ-HB	108	1.38	0.26	1.40	0.30	0.56	0.07	–	–
AIHQ-AB	108	1.40	0.31	1.37	0.29	0.24	−0.09	–	–
AIHQ-BS	108	2.92	0.68	2.93	0.73	0.76	0.01	–	–
IPSAQ-EB	107	1.67	4.40	1.70	4.49	0.99	0.01	–	–
IPSAQ-PB	107	0.56	0.33	0.52	0.34	0.24	−0.11	–	–
MSCEIT-PE	108	0.46	0.14	0.46	0.15	0.99	0.01	–	–
MSCEIT-FE	107	0.43	0.11	0.42	0.12	0.77	−0.06	–	–
MSCEIT-UE	108	0.43	0.10	0.44	0.11	0.51	0.02	–	–
MSCEIT-ME	108	0.32	0.09	0.32	0.10	0.92	0.00	–	–
TASIT-1	108	19.99	4.08	20.38	4.03	0.28	0.10	0/0	0/3
TASIT-2	108	38.96	7.90	37.49	7.36	0.06	−0.19	17/0	15/0
TASIT-3	108	40.93	7.43	40.94	7.06	0.78	0.00	14/0	15/0
ER40	106	30.19	3.82	30.48	4.70	0.30	0.07	0/0	0/0
Controls (*n* = 70)									
Hinting	70	15.63	2.50	15.91	2.20	0.37	0.12	0/5	0/2
BLERT	70	16.83	2.34	17.24	2.20	0.15	0.18	0/0	0/2
MiniPONS	70	47.80	4.79	49.63	4.47	< 0.01	0.39	0/0	0/0
RAD	70	32.93	5.13	33.34	5.47	0.30	0.08	2/0	3/0
AIHQ-HB	70	1.41	0.25	1.34	0.22	0.01	−0.30	–	–
AIHQ-AB	70	1.36	0.21	1.35	0.26	0.56	−0.02	–	–
AIHQ-BS	70	2.85	0.54	2.82	0.54	0.65	−0.06	–	–
IPSAQ-EB	70	1.63	3.97	2.11	3.88	0.29	0.12	–	–
IPSAQ-PB	70	0.44	0.28	0.42	0.27	0.28	−0.07	–	–
MSCEIT-PE	70	0.55	0.10	0.54	0.14	0.54	−0.04	–	–
MSCEIT-FE	70	0.47	0.08	0.47	0.10	0.63	0.03	–	–
MSCEIT-UE	70	0.50	0.08	0.51	0.08	0.34	0.09	–	–
MSCEIT-ME	70	0.36	0.08	0.35	0.08	0.59	−0.12	–	–
TASIT-1	70	22.73	2.73	23.40	3.16	0.03	0.23	0/0	0/3
TASIT-2	70	49.76	6.15	44.67	8.41	< 0.01	−0.69	0/3	3/1
TASIT-3	70	50.26	6.72	50.17	6.72	0.77	−0.01	1/0	0/0
ER40	70	33.57	3.05	33.74	3.25	0.57	0.05	0/0	0/1

Note. BLERT = Bell Lysaker Emotion Recognition Task; MiniPONS = Mini Profile of Nonverbal Sensitivity; RAD = Relationships Across Domains; AIHQ-HB = Ambiguous Intentions and Hostility Questionnaire – Hostility Bias; AIHQ-AB = Ambiguous Intentions and Hostility Questionnaire – Aggression Bias; AIHQ-BS = Ambiguous Intentions and Hostility Questionnaire – Blame Score; IPSAQ-EB = Internal Personal and Situational Attributions Questionnaire – Externalizing Bias; IPSAQ-PB = Internal Personal and Situational Attributions Questionnaire – Personalizing Bias; MSCEIT-PE = Mayer-Salovey-Caruso Emotional Intelligence Test – Perceiving Emotions; MSCEIT-FE = Mayer-Salovey-Caruso Emotional Intelligence Test – Facilitating Emotions; MSCEIT-UE = Mayer-Salovey-Caruso Emotional Intelligence Test – Understanding Emotions; MSCEIT-ME = Mayer-Salovey-Caruso Emotional Intelligence Test – Managing Emotions; TASIT-1 = The Awareness of Social Inference Test – Branch 1; TASIT-2 = The Awareness of Social Inference Test – Branch 2; TASIT-3 = The Awareness of Social Inference Test – Branch 3; ER40 = Penn Emotion Recognition Task.

RAD、TASIT で床効果が認められた。

Mini-PONS の学習効果が高かった。

４．妥当性

(Relationship to social cognitive measures relative to other abilities and constructs)

※この項目では、他の社会認知機能との関連が強い尺度が良いと考える。

Table 5
Case-control differences on social cognitive measures.

	Cases (n = 116)		Controls (n = 73)		Control-case		P	Cohen's d
	Mean	SD	Mean	SD	Mean	SE		
Hinting	14.12	3.41	15.52	2.58	1.40	0.44	< 0.01	0.46
BLERT	14.14	3.33	16.77	2.37	2.63	0.41	< 0.01	0.91
MiniPONS	43.00	5.91	47.66	4.81	4.66	0.79	< 0.01	0.86
RAD	27.71	6.18	32.85	5.05	5.14	0.83	< 0.01	0.91
AIHQ-HB	1.37	0.25	1.41	0.25	0.04	0.04	0.21	0.16
AIHQ-AB	1.40	0.30	1.36	0.20	−0.05	0.04	0.57	−0.18
AIHQ-BS	2.93	0.67	2.86	0.53	−0.06	0.09	0.29	−0.10
IPSAQ-EB	1.59	4.31	1.85	4.19	0.26	0.64	0.52	0.06
IPSAQ-PB	0.56	0.33	0.44	0.28	−0.12	0.04	0.02	−0.38
MSCEIT-PE	0.46	0.14	0.55	0.10	0.09	0.02	< 0.01	0.74
MSCEIT-FE	0.42	0.11	0.47	0.08	0.05	0.01	< 0.01	0.49
MSCEIT-UE	0.43	0.11	0.50	0.08	0.07	0.01	< 0.01	0.75
MSCEIT-ME	0.31	0.09	0.36	0.08	0.05	0.01	< 0.01	0.58
TASIT-1	19.82	4.08	22.78	2.72	2.96	0.50	< 0.01	0.85
TASIT-2	38.60	7.94	49.79	6.12	11.19	1.03	< 0.01	1.58
TASIT-3	40.63	7.42	50.21	6.89	9.58	1.08	< 0.01	1.34
ER40	30.03	4.06	33.52	3.01	3.49	0.52	< 0.01	0.98

Note. BLERT = Bell Lysaker Emotion Recognition Task; MiniPONS = Mini Profile of Nonverbal Sensitivity; RAD = Relationships Across Domains; AIHQ-HB = Ambiguous Intentions and Hostility Questionnaire – Hostility Bias; AIHQ-AB = Ambiguous Intentions and Hostility Questionnaire – Aggression Bias; AIHQ-BS = Ambiguous Intentions and Hostility Questionnaire – Blame Score; IPSAQ-EB = Internal Personal and Situational Attributions Questionnaire – Externalizing Bias; IPSAQ-PB = Internal Personal and Situational Attributions Questionnaire – Personalizing Bias; MSCEIT-PE = Mayer-Salovey-Caruso Emotional Intelligence Test – Perceiving Emotions; MSCEIT-FE = Mayer-Salovey-Caruso Emotional Intelligence Test – Facilitating Emotions; MSCEIT-UE = Mayer-Salovey-Caruso Emotional Intelligence Test – Understanding Emotions; MSCEIT-ME = Mayer-Salovey-Caruso Emotional Intelligence Test – Managing Emotions; TASIT-1 = The Awareness of Social Inference Test – Branch 1; TASIT-2 = The Awareness of Social Inference Test – Branch 2; TASIT-3 = The Awareness of Social Inference Test – Branch 3; ER40 = Penn Emotion Recognition Task.

本研究では、健常者との比較のみ評価している。

(Correlations with Real-World Social Outcomes)

本研究では神経認知機能、社会機能との関連を評価していない。

III. 候補検査の計量心理学的指標

1. Bell Lysaker Emotion Recognition Task (BLERT)

1. 実用可能性と忍容性

(Practicality for Administration and Tolerability for Patients)

Notes:

Notes: The BLERT contains 21, 10 second video clips allowing for a minimum administration time of approximately 3.5 minutes. Approximately 5 seconds is provided between clips.

２. 信頼性

(Test-Retest, Internal Consistency, Inter-rater Reliability as applicable)

Author and Date	Sample	Findings
Bell et al. (1997)	50 SCZ; 25 non-psychotic individuals with substance abuse; 81 college student controls	At 5 month follow-up, categorical stability, categorizing participant performance as normal, mild impairment, moderate impairment, moderately severe or severe, was .94 (weighted k)<br>Test-retest reliability was .76 (Pearson‘s r)
Roberts et al. (2009)	31 SCZ	α =.77
Hamm et al. (2012)	49 SCZ/SCZaff	Six month test-retest reliability was .54
Pinkham and Penn (2006)	49 patients (35 SCZ, 12 SCZaff, 2 Psychosis NOS); 44 HC	α =.73

３. 有用性

(Floor and Ceiling Effects, Normality of Distributions)

Author and Date	Sample	Findings
Bell et al. (1997)	50 SCZ; 25 non-psychotic individuals with substance abuse;	Mean percents correct were 92.3% for controls, 77.2% for substance abuse, and 64.8% for SCZ<br>No SDs reported.

	81 college student controls	
Bryson et al. (2003)	96 SCZ/SCZaff	Mean number correct was 12.95 (SD=3.75) out of 21. Thus approx. 61.7% correct.
Combs et al. (2004)	65 SCZ	Mean number correct was 12.1 (SD=4.4) out of 21. Thus approx. 57.6% correct.
Combs et al. (2004)	60 healthy students (29 high in subclinical paranoia; 31 low in subclinical paranoia)	High paranoia mean 12.0 (approximately 57% correct; SD= 8.6%); Low paranoia mean was 13.5 (approximately 64% correct; SD=6.7%)
Fiszdon and Johannesen (2010)	48 SSIs, 56 HC	HC mean: 17.18 (SD = 1.29)<br>SCZ mean: 11.39 (SD=3.13)

(Utility as a Repeated Measure)
※この項目では、自然経過では不変で介入によってより大きく動く尺度が良いと考える。

Author and Date	Sample	Treatment Study?<br>Testing Interval	Findings
Bell et al. (2001)	65 SCZ/SCZaff randomly assigned to neurocognitive enhancement training plus work therapy (NET+WT) or work therapy alone (WT)	Yes<br>5 months	20 NET+WT patients (65%) had small or large effect-size improvements compared to 10 WT patients (29%).<br>12 NET+WT patients (39%) had large effect-size changes compared to 3 WT patients (9%).
Wexler et al. (2005)	145 SCZ/SCZaff randomly assigned to cognitive remediation training plus	Yes<br>6 months	The percentage of CRT + WT subjects with normal scores on the BLERT increased from 35 to 60%.<br>The percentage of WT patients with normal scores declined from 47 to 42%.

	work therapy (CRT+WT) or work therapy alone (WT)		
Hamm et al. (2012)	49 SCZ/SCZaff	No<br>6 months	No significant change between Time 1 (M=12.57, SD=3.28) and Time 2 (M=12.35, SD=3.81).<br>36 participants had less than a 33% change, while 13 had a change from baseline to follow-up of 33%-67%.
Roberts et al. (2009)	10 SCZ in Social Cognition and Interaction Training plus TAU (SCIT+TAU); 7 SCZ in TAU alone	Yes<br>20 weeks	SCIT effect size d = .29<br>TAU effect size d = -.19

４．妥当性

(Relationship to social cognitive measures relative to other abilities and constructs)

※この項目では、他の社会認知機能との関連が強い尺度が良いと考える。

Author and Date	Sample	Findings
Bell et al. (2010)	66 patients (49 SCZ, 16 SCZaff, 1 Psychosis NOS); 85 controls not screened for psychopathology	Correlated significantly (r=.37, p = .0002) with the Social Attribution Test-Multiple Choice (SAT-MC)
Combs et al. (2004)	65 SCZ	BLERT and FEIT were found to be very highly correlated r = .85, p < .0001;<br>Attention predicted affect recognition (R_2=.786) as defined by combined BLERT and FEIT
Hamm et al. (2012)	49 SCZ/SCZaff	Baseline BLERT correlated .46 with Metacognitive Assessment Scale abbreviated (MAS-A) and .43 at 6 month MAS-A<br>Correlated .29 (p<.05) with Wisconsin Card Sort
Pinkham and Penn (2006)	49 patients (35 SCZ, 12 SCZaff, 2 Psychosis NOS); 44 HC	In HC:<br>Social cognitive tasks: ns with FEIT, r=.476 with FEDT, ns with SCST, r=.387 with SCST time, ns

	Note: SCST = Schema Component Sequencing Task	with Hinting and ToM vignettes<br>Neurocognitive tasks: r=.49 with WRAT, ns with Immediate Memory, ns with Trails A, r=-.44 with Trails B,<br>In patients:<br>Social cognitive tasks: r=.373 with FEIT, r=.326 with FEDT, r=.418 with ToM vignettes, ns with SCST #, SCST Time, Hinting;<br>Neurocognitive tasks: r=.47 with WRAT, ns with TrailsA, TrailsB
Bell et al. (2009)	151 SZ	r = .17 with Hinting Task,<br>r=.18 with WCST, r=.17 with digit span test<br>All correlations significant at p<.05

(Correlations with Real-World Social Outcomes)

Author and Date	Sample	Findings
Bell et al. (2001)	33 SCZ	The BLERT failed to relate to Social Skills as measured by the Work Behavior Inventory
Pinkham and Penn (2006)	49 patients (35 SCZ, 12 SCZaff, 2 Psychosis NOS); 44 HC	r=.368 (p<.05) with interpersonal skill (Conversation probe role play) in HC, and r=.38 (p<.01) with interpersonal skill in patients
Nienow et al. (2006)	56 SCZ	r = .31 (p<.05) with AIPSS social problem solving
Bell et al. (2009)	151 SCZ	No significant correlations with perceived social discomfort at work, or composite rehabilitation outcomes
Fiszdon and Johannesen (2010)	48 SSIs, 56 HCs	r = .49 (p<.01) with UPSA, .r=52 (p<.01) with the medication management ability assessment or MMAA, ns correlations with SSPA, QLS, and ILSS

文献

ORIGINAL CITATION:

Title: Positive and negative affect recognition in schizophrenia: a comparison with substance abuse and normal control subjects

Author(s): Bell, M ; Bryson, G ; Lysaker, P

Source: PSYCHIATRY RESEARCH Volume: 73 Issue: 1-2 Pages: 73-82 DOI: 10.1016/S0165-1781(97)00111-X Published: NOV 14 1997

Times Cited: 84 (from All Databases)

CITING ARTICLES

Title: Affect recognition in schizophrenia: A function of global impairment or a specific cognitive deficit

Author(s): Bryson, G; Bell, M; Lysaker, P

Source: PSYCHIATRY RESEARCH Volume: 71 Issue: 2 Pages: 105-113 DOI: 10.1016/S0165-1781(97)00050-4 Published: JUL 4 1997

Times Cited: 110 (from All Databases)

Title: Positive and negative affect recognition in schizophrenia: a comparison with substance abuse and normal control subjects

Author(s): Bell, M; Bryson, G; Lysaker, P

Source: PSYCHIATRY RESEARCH Volume: 73 Issue: 1-2 Pages: 73-82 DOI: 10.1016/S0165-1781(97)00111-X Published: NOV 14 1997

Times Cited: 84 (from All Databases)

Title: Neurocognitive enhancement therapy with work therapy - Effects on neuropsychological test performance

Author(s): Bell, M; Bryson, G; Greig, T; et al.

Source: ARCHIVES OF GENERAL PSYCHIATRY Volume: 58 Issue: 8 Pages: 763-768 DOI: 10.1001/archpsyc.58.8.763 Published: AUG 2001

Times Cited: 134 (from All Databases)

Title: Work Rehabilitation in Schizophrenia: Does Cognitive Impairment Limit Improvement?

Author: Morris D. Bell and Gary Bryson

Source: Schizophr Bull (2001) 27(2): 269-279

Title: Cognitive remediation and vocational rehabilitation for schizophrenia

Author(s): Wexler, BE; Bell, MD

Conference: 7th Biennial Sinai Conference on Cognition in Schizophrenia Location: Savannah, GA Date: APR 02, 2005

Source: SCHIZOPHRENIA BULLETIN Volume: 31 Issue: 4 Pages: 931-941 DOI: 10.1093/schbul/sbi038 Published: OCT 2005

Times Cited: 68 (from All Databases)

Title: Social attribution test - multiple choice (SAT-MC) in schizophrenia: Comparison with community sample and relationship to neurocognitive, social cognitive and symptom measures

Author(s): Bell, Morris D.; Fiszdon, Joanna M.; Greig, Tamasine C.; et al.

Source: SCHIZOPHRENIA RESEARCH Volume: 122 Issue: 1-3 Pages: 164-171 DOI: 10.1016/j.schres.2010.03.024 Published: SEP 2010

Times Cited: 2 (from All Databases)

Title: Initial and final work performance in schizophrenia: Cognitive and symptom predictors
Author(s): Bryson, G; Bell, MD
Source: JOURNAL OF NERVOUS AND MENTAL DISEASE Volume: 191 Issue: 2 Pages: 87-92 DOI: 10.1097/01.NMD.0000050937.06332.3C Published: FEB 2003
Times Cited: 70 (from All Databases)
Title: The role of attention in affect perception: An examination of Mirsky's four factor model of attention in chronic schizophrenia
Author(s): Combs, DR; Gouvier, WD
Source: SCHIZOPHRENIA BULLETIN Volume: 30 Issue: 4 Pages: 727-738 Published: 2004
Times Cited: 12 (from All Databases)
Title: The role of subclinical paranoia on social perception and behavior
Author(s): Combs, DR; Penn, DL
Source: SCHIZOPHRENIA RESEARCH Volume: 69 Issue: 1 Pages: 93-104 DOI: 10.1016/S0920-9964(03)00051-3 Published: JUL 1 2004
Times Cited: 36 (from All Databases)
Title: A comparison of basic and social cognition between schizophrenia and schizoaffective disorder
Author(s): Fiszdon, J. M.; Richardson, R.; Greig, T.; et al.
Conference: 10th International Congress on Schizophrenia Research Location: Savannah, GA Date: APR 02-06, 2005
Source: SCHIZOPHRENIA BULLETIN Volume: 33 Issue: 2 Pages: 558-558 Published: MAR 2007
Times Cited: 0 (from All Databases)
Title: Metacognition and Social Cognition in Schizophrenia: Stability and Relationship to Concurrent and Prospective Symptom Assessments
Author: Jay A. Hamm[1], Selwyn B. Renard[2], Rebecca L. Fogley[1], Bethany L. Leonhardt[1], Giancarlo Dimaggio[3], Kelly D. Buck[4], Paul H. Lysaker[5,*]

Source: Journal of Clinical Psychology Article first published online: 8 AUG 2012
DOI: 10.1002/jclp.21906
Title: Suspiciousness and low self-esteem as predictors of misattributions of anger in schizophrenia spectrum disorders
Author(s): Lysaker, Paul Henry; Davis, Louanne Whitman; Tsai, Jack
Source: PSYCHIATRY RESEARCH Volume: 166 Issue: 2-3 Pages: 125-131 DOI: 10.1016/j.psychres.2008.03.014 Published: APR 30 2009
Times Cited: 6 (from All Databases)
Title: Neurocognitive deficits and history of childhood abuse in schizophrenia spectrum disorders: associations with Cluster B personality traits
Author(s): Lysaker, PH; Wickett, AM; Lancaster, RS; et al.
Source: SCHIZOPHRENIA RESEARCH Volume: 68 Issue: 1 Pages: 87-94 DOI: 10.1016/S0920-

9964(03)00195-6 Published: MAY 1 2004

Times Cited: 21 (from All Databases)

Title: Neurocognitive and social cognitive predictors of interpersonal skill in schizophrenia

Author(s): Pinkham, Amy E.; Penn, David L.

Source: PSYCHIATRY RESEARCH Volume: 143 Issue: 2-3 Pages: 167-178 DOI: 10.1016/j.psychres.2005.09.005 Published: AUG 30 2006

Times Cited: 70 (from All Databases)

Title: Social cognition and interaction training (SCIT) for outpatients with schizophrenia: A preliminary study

Author(s): Roberts, David L.; Penn, David L.

Source: PSYCHIATRY RESEARCH Volume: 166 Issue: 2-3 Pages: 141-147 DOI: 10.1016/j.psychres.2008.02.007 Published: APR 30 2009

Times Cited: 23 (from All Databases)

2. Penn Emotion Recognition Task (ER-40)

1. 実用可能性と忍容性

(Practicality for Administration and Tolerability for Patients)

Author and Date	Sample	Notes
Carter et al., 2009	1023 SCZ; 424 HC	Average testing time is under 5 minutes.<br>Computerized task administered via dedicated hardware or the internet.<br>Automated scoring provides accuracy and median response times.

２. 信頼性

(Test-Retest, Internal Consistency, Inter-rater Reliability as applicable)

Author and Date	Sample	Findings
Carter et al. 2009	13 SCZ; 6 HC	Test-retest reliability (no time frame provided) is .80 for HC and .76 in SCZ
Gur et al. (2010)	448 HC (226 females)	α=.91

３. 有用性

(Floor and Ceiling Effects, Normality of Distributions)

Author and Date	Sample	Findings
Carter et al., 2009	1023 SCZ; 424 HC	Mean percents correct were 73.1% for patients and 84% for controls. SDs not available. No SDs reported.
Kohler et al. (2003)	28 SCZ outpatients; 61 healthy comparison subjects	Mean percents correct were 63.6% for patients and 71% for control subjects. There was a ceiling effect for happy expressions (97.1% correct for patients, 98.4% for controls). No SDs reported.
Pinkham et al. (2008)	270 SCZ; 270 HC	Mean percents correct were 75.6% for patients and 82.8% for controls. No SDs reported.
Gur et al. (2007)	16 SCZ; 17 HC	Mean percent correct for patients ranged from 90.66% (SD=10.72%) for happy to 76.90% (SD=13.47%) for fear. Controls ranged from 95.97% (SD=5.21%) for happy to 82.42% (SD=12.11%) for fear.

Silver et al. (2004)	20 male chronic SCZ	Mean % correct at pretest was 68.75% (SD= 14.25%) and 76% (SD=10.25%) at post test.
Sasson et al. (2010)	7,320 individuals (1,989 male, 5,331 female)<br>- recruited online so clinical status uncertain	Mean total number correct ranged from 81.14% (SD=7.33%) to 85.70% (SD=6.81%) across the entire sample. Males scored 93.56% (SD=7.30) correct on high intensity stimuli and 70.41% (SD=12.47%) on low intensity. Females scored 95.04% (6.11%) correct on high intensity and 75.00% (11.97%) on low intensity.
Pinkham et al. (2011)	132 SCZ	Mean % correct ranging from 93% (SE=1.3%) for happy to 63% (SE=2.2% for anger
Kohler et al. (2005)	20 Alzheimers patients (M:F = 11:9); 22 healthy caretakers (M:F = 9:13)	Mean % correct was 66.75% (SD=13%) in Alzheimer patients; 82% (SD= 8%) in caretakers.
Goodman et al. (2005)	35 male forensic SCZ	Mean % correct ranged from 65.7% (SD=12.55%) to 71% (11.55%)
Gur et al. (2010)	448 HC (226 females)	Mean percent correct: 84% (SD=7.8%)
Irani et al. (2012)	624 SCZ; 624 HC.	Z-scores reported only: -.25 young HC (under age 45,) -.1 older HC (over 45), -1.0 and greater for patients across ages
Roddy et al. (2012)	793 children aged 10–13 years	Mean number correct 30.38 (S.D=3.95) out of 40. Thus approx. 76% correct.
Weiss et al. (2007)	100 healthy students	Mean % correct ranged from 97-98% (SD=3.5%) for happy, 55-61% (SD=8%) for anger, 75-85% (SD=6%) for fear, and 70-82% (SD= 8%) for sad. Average standard deviations extrapolated from figure.
Weiss et al. (2007)	56 chronic, inpatient SCZ	Mean percents correct were: happy expressions (92.6%) (95% CI 89.9-94.9%), fear (61.4%) (95% CI 56.7-65.9%), neutral (60.9%) (95% CI 56.3-65.5%), sad (59.8%) (95% CI 55.1-64.4%), and anger (55.6%) (95% CI<br>50.8-60.2)

(Utility as a Repeated Measure)

※この項目では、自然経過では不変で介入によってより大きく動く尺度が良いと考える。

Author and Date	Sample	Treatment Study?	Findings

		Testing Interval	
Silver et al. (2004)	20 male chronic SCZ	Yes<br>3 days	Significant change due to 3 days of brief emotion training (pre-test mean 27.5, SD=5.7; post-test mean 30.4, SD=4.1; *t*=-2.67, *p*=.02, *d*=.60).

４．妥当性

(Relationship to social cognitive measures relative to other abilities and constructs)

Please note that for this criterion, the candidate measure should be more strongly correlated to other measures of social cognition than to non-social cognitive tasks.

Author and Date	Sample	Findings
Gur et al. (2010)	448 HC (226 females)	The ER40 significantly correlated with immediate face memory (r=.31) and delayed face memory (r=.29).<br>Significantly correlated with a number of other neurocognitive tests in the Penn CNB at p<.01: PCET (r=.27), CPT L (r=.26), LNB (r=.29), Spatial IMM (r=.34), spatial DEL (r=.31).<br>Significant at p<.001 with PVRT (r=.36) and CJOLO (r=.35)
Roddy et al. (2012)	793 children aged 10–13 years	Recognition of sad faces was significantly correlated with the Hinting Task (*p*=0.031)

(Correlations with Real-World Social Outcomes)

Author and Date	Sample	Findings
Grant and Beck (2010)	123 SCZ/SCZaff	Emotion perception (comprised of ER40 and Penn emotion discrimination test) was significantly correlated with vocational functioning (r=.28, p<.01), but not social functioning (r=-.06), measured with the SFS.

文献

ORIGINAL CITATION:

Title: FACIAL EMOTION DISCRIMINATION .1. TASK CONSTRUCTION AND BEHAVIORAL FINDINGS IN NORMAL SUBJECTS

Author(s): ERWIN, RJ; GUR, RC; GUR, RE; et al.

Source: PSYCHIATRY RESEARCH Volume: 42 Issue: 3 Pages: 231-240 DOI: 10.1016/0165-1781(92)90115-J Published: JUN 1992

Times Cited: 135 (from All Databases)

CITING ARTICLES

Title: CNTRICS Final Task Selection: Social Cognitive and Affective Neuroscience-Based Measures

Author(s): Carter, Cameron S.; Barch, Deanna M.; Gur, Ruben; et al.

Source: SCHIZOPHRENIA BULLETIN Volume: 35 Issue: 1 Pages: 153-162 DOI: 10.1093/schbul/sbn157 Published: JAN 2009

Times Cited: 26 (from All Databases)

Title: Facial emotion recognition in schizophrenia: Intensity effects and error pattern

Author(s): Kohler, CG; Turner, TH; Bilker, WB; et al.

Conference: 57th Annual Meeting of the Society-of-Biological-Psychiatry Location: PHILADELPHIA, PENNSYLVANIA Date: MAY 16-18, 2002

Sponsor(s): Soc Biol Psychiat

Source: AMERICAN JOURNAL OF PSYCHIATRY Volume: 160 Issue: 10 Pages: 1768-1774 DOI: 10.1176/appi.ajp.160.10.1768 Published: OCT 2003

Times Cited: 196 (from All Databases)

Title: A method for obtaining 3-dimensional facial expressions and its standardization for use in neurocognitive studies

Author(s): Gur, RC; Sara, R; Hagendoorn, M; et al.

Source: JOURNAL OF NEUROSCIENCE METHODS Volume: 115 Issue: 2 Pages: 137-143 Article Number: PII S0165-0270(02)00006-7 DOI: 10.1016/S0165-0270(02)00006-7 Published: APR 15 2002

Times Cited: 146 (from All Databases)

Title: The other-race effect in face processing among African American and Caucasian individuals with schizophrenia

Author(s): Pinkham, Amy E.; Sasson, Noah J.; Calkins, Monica E.; et al.

Source: AMERICAN JOURNAL OF PSYCHIATRY Volume: 165 Issue: 5 Pages: 639-645 DOI: 10.1176/appi.ajp.2007.07101604 Published: MAY 2008

Times Cited: 21 (from All Databases)

Title: Limbic activation associated with misidentification of fearful faces and flat affect in schizophrenia

Author(s): Gur, Raquel E.; Loughead, James; Kohler, Christian G.; et al.

Conference: 61st Annual Convention of the Society-of-Biological-Psychiatry Location: Toronto, CANADA Date: MAY 18-20, 2006

Sponsor(s): Soc Biol Psychiat

Source: ARCHIVES OF GENERAL PSYCHIATRY Volume: 64 Issue: 12 Pages: 1356-+ DOI: 10.1001/archpsyc.64.12.1356 Published: DEC 2007

Times Cited: 62 (from All Databases)

Title: Brief emotion training improves recognition of facial emotions in chronic schizophrenia. A pilot study

Author(s): Silver, H; Goodman, C; Knoll, G; et al.

Source: PSYCHIATRY RESEARCH Volume: 128 Issue: 2 Pages: 147-154 DOI: 10.1016/j.psychres.2004.06.002 Published: SEP 30 2004

Times Cited: 39 (from All Databases)

Title: Controlling for Response Biases Clarifies Sex and Age Differences in Facial Affect Recognition

Author(s): Sasson, Noah J.; Pinkham, Amy E.; Richard, Jan; et al.

Source: JOURNAL OF NONVERBAL BEHAVIOR Volume: 34 Issue: 4 Pages: 207-221 DOI: 10.1007/s10919-010-0092-z Published: DEC 2010

Times Cited: 3 (from All Databases)

Jaracz et al.: Facial emotion recognition in schizophrenia: neuropsychological and psychosocial correlates. Annals of General Psychiatry 2010 9(Suppl 1):S206.

Title: Actively paranoid patients with schizophrenia over attribute anger to neutral faces

Author(s): Pinkham, Amy E.; Brensinger, Colleen; Kohler, Christian; et al.

Source: SCHIZOPHRENIA RESEARCH Volume: 125 Issue: 2-3 Pages: 174-178 DOI: 10.1016/j.schres.2010.11.006 Published: FEB 2011

Times Cited: 1 (from All Databases)

Title: Association between facial emotion recognition and odor identification in schizophrenia

Author(s): Kohler, Christian G.; Barrett, Frederick S.; Gur, Ruben C.; et al.

Source: JOURNAL OF NEUROPSYCHIATRY AND CLINICAL NEUROSCIENCES Volume: 19 Issue: 2 Pages: 128-131 DOI: 10.1176/appi.neuropsych.19.2.128 Published: MAY 2007

Times Cited: 3 (from All Databases)

Title: Emotion-discrimination deficits in mild Alzheimer disease

Author(s): Kohler, CG; Anselmo-Gallagher, G; Bilker, W; et al.

Conference: Meeting of the Psychiatry-Research-Society Location: Park City, UT Date: FEB 11-14, 2004

Sponsor(s): Psychiatry Res Soc

Source: AMERICAN JOURNAL OF GERIATRIC PSYCHIATRY Volume: 13 Issue: 11 Pages: 926-933 DOI: 10.1176/appi.ajgp.13.11.926 Published: NOV 2005

Times Cited: 23 (from All Databases)

Title: Social Cognition Deficits Among Individuals at Familial High Risk for Schizophrenia

Author(s): Eack, Shaun M.; Mermon, Diana E.; Montrose, Debra M.; et al.

Source: SCHIZOPHRENIA BULLETIN Volume: 36 Issue: 6 Pages: 1081-1088 DOI: 10.1093/schbul/sbp026 Published: NOV 2010

Times Cited: 11 (from All Databases)

Title: Insight into illness in schizophrenia

Author(s): Goodman, C; Knoll, G; Isakov, V; et al.

Source: COMPREHENSIVE PSYCHIATRY Volume: 46 Issue: 4 Pages: 284-290 DOI: 10.1016/j.comppsych.2004.11.002 Published: JUL-AUG 2005

Times Cited: 21 (from All Databases)

Title: Asocial beliefs as predictors of asocial behavior in schizophrenia

Author(s): Grant, Paul M.; Beck, Aaron T.

Source: PSYCHIATRY RESEARCH Volume: 177 Issue: 1-2 Pages: 65-70 DOI: 10.1016/j.psychres.2010.01.005 Published: MAY 15 2010

Times Cited: 4 (from All Databases)

Title: A cognitive neuroscience-based computerized battery for efficient measurement of individual differences: Standardization and initial construct validation

Author(s): Gur, Ruben C.; Richard, Jan; Hughett, Paul; et al.

Source: JOURNAL OF NEUROSCIENCE METHODS Volume: 187 Issue: 2 Pages: 254-262 DOI: 10.1016/j.jneumeth.2009.11.017 Published: MAR 30 2010

Times Cited: 17 (from All Databases)

Title: Computerized Neurocognitive Test Performance in Schizophrenia: A Lifespan Analysis

Author(s): Irani, Farzin; Brensinger, Colleen M.; Richard, Jan; et al.

Source: AMERICAN JOURNAL OF GERIATRIC PSYCHIATRY Volume: 20 Issue: 1 Pages: 41-52 DOI: 10.1097/JGP.0b013e3182051a7d Published: JAN 2012

Times Cited: 1 (from All Databases)

S. Roddy, L. Tiedt, I. Kelleher, M. C. Clarke, J. Murphy, C. Rawdon, R. A. P. Roche, M. E. Calkins, J. A. Richard, C. G. Kohler and M. Cannon Facial emotion recognition in adolescents with psychotic-like experiences: a school-based sample from the general population. Psychological Medicine, Available on CJO 2012 doi:10.1017/S0033291712000311

Title: Differential effect of catechol-O-methyltransferase Val(158) Met genotype on emotional recognition abilities in healthy men and women

Author(s): Weiss, Elisabeth M.; Stadelmann, Edith; Kohler, Christian G.; et al.

Source: JOURNAL OF THE INTERNATIONAL NEUROPSYCHOLOGICAL SOCIETY Volume: 13 Issue: 5 Pages: 881-887 Published: SEP 2007

Times Cited: 9 (from All Databases)

Title: Gender differences in facial emotion recognition in persons with chronic schizophrenia

Author(s): Weiss, Elisabeth M.; Kohler, Christian G.; Brensinger, Colleen M.; et al.

Source: EUROPEAN PSYCHIATRY Volume: 22 Issue: 2 Pages: 116-122 DOI: 10.1016/j.eurpsy.2006.05.003 Published: MAR 2007

Times Cited: 15 (from All Databases)

Title: Controlling for Response Biases Clarifies Sex and Age Differences in Facial Affect Recognition

Author(s): Sasson, Noah J.; Pinkham, Amy E.; Richard, Jan; et al.

Source: JOURNAL OF NONVERBAL BEHAVIOR Volume: 34 Issue: 4 Pages: 207-221 DOI: 10.1007/s10919-010-0092-z Published: DEC 2010

Times Cited: 3 (from All Databases)

3. The Hinting Task

1. 実用可能性と忍容性

(Practicality for Administration and Tolerability for Patients)

Notes:

Notes: This is a paper and pencil test. It comprises 10 vignettes in which a person drops a hint. Probably takes no more than 10 minutes or so. Scoring is generally straightforward and quick.

２．信頼性

(Test-Retest, Internal Consistency, Inter-rater Reliability as applicable)

Author and Date	Sample	Findings
Roberts and Penn (2009)	31 SSI	α = 0.65.
Liu et al. (2011)	180 SSI	α = 0.59.
Roberts and Penn (2009)	31 SSI	In the treatment as usual (TAU) group, hinting task demonstrated good test-retest reliability (effect size of d=-.06, pre-test mean 15.45 (SD=2.94), post-test mean 15.27 (SD=3.38) (over 6 months)
Lysaker et al. (2011)	36 SCZ/SCZaff	M=11.89 (4.80) baseline; M=12.89 (4.40) retest.

３．有用性

(Floor and Ceiling Effects, Normality of Distributions)

Author and Date	Sample	Findings
Corcoran, Mercer, and Frith (1995)	55 SCZ<br>30 healthy controls	SCZ = 78% correct (SD=19.5%)<br>HC = 91.5% correct (SD=8%)
Bertrand et al. (2007)	36 SSI (first episode)<br>25 HC	SSI = 76.55% correct (15.55%)<br>HC = 90.35% correct (7.35%)
Corcoran (2003)	39 SCZ<br>44 HC	SCZ = 82.5% (20%) correct<br>HC = 92.5% (6%) correct
Bora et al. (2008)	91 SCZ<br>55 HC	SCZ = 74% (22.5%) correct<br>HC = 80% (17%) correct

Corcoran and Frith (2003)	59 SCZ<br>44 HC	SCZ = 74.3% (26.6%) correct<br>HC = 94.5% (5.1%) correct
Greig et al. (2004)	128 SCZ/SCZaff	SCZ/SCZaff = 80.4% (19.15%) correct
Liu et al. (2011)	180 SSI	Ranges from 73%(16.5%) at intake to 77.2%(20.5%) 12 months later
Lysaker et al. (2011)	65 SSI	Mean 11.17 (4.80)
Menon, Addington, and Remington (2011)	18 SCZ or SCZaff<br>17 HC	SCZ/SCZaff = 16.67 (3.6)<br>HC = 19.18 (1.1)
Meijer et al. (2012)	1093 SSI<br>1044 unaffected siblings<br>911 Parents<br>587 HC	SSI = 17.54 (2.78)<br>Siblings = 18.84 (1.66)<br>Parents = 18.79 (1.62)<br>HC = 19.08 (1.31)
Uhlhaas et al. (2006)	12 SCZ (disorganized)<br>36 SCZ (non-disorganized)<br>26 non-psychotic patients-controls	Z scores only; scz disorganized type -1.2, scz nondisorganized -.2, controls .65
Silverstein, Wallace, and Schenkel (2005)	26 SCZ	Scores on the Hinting Task were negatively skewed (i.e., absolute values of the ratio of skewness to standard error of skewness > 2).
Mizrahi et al. (2007)	71 SSI	16.82 (S.D.=2.8) at baseline.
Marjoram et al. (2005)	15 SCZ<br>15 affective disorder<br>15 HC	SCZ = 15.5 (2.2)<br>Affective disorder = 18.2 (1.7)<br>HC = 19.2 (1.1)
Couture et al. (2011)	178 SCZ/SCZaff	13.2 (4.5)
Bell et al. (2008)	151 SCZ/SCZaff	16.02(3.68)
Bell et al.	77 SCZ/SCZaff	All subjects 16.7 (2.2); high negative symptoms 15.7 (2.4);

(2011)		higher social cognition 17.3 (2.0); lower social cognition 16.9 (2.0)

(Utility as a Repeated Measure)

※この項目では、自然経過では不変で介入によってより大きく動く尺度が良いと考える。

Author and Date	Sample	Treatment study? Testing interval	Findings
Roberts and Penn (2009)	31 SSI	Yes<br>20 weeks (SCIT versus TAU)	SCIT: Pretest = 16.14 (2.66) Posttest = 15.92 (2.59)<br>TAU: Pretest = 15.45 (2.94) Posttest = 15.27 (3.38)<br>No significant treatment effect
Tas et al. (2012)	52 SCZ	Yes<br>14 weeks<br>-SCIT versus social stimulation	**SCIT**<br>Pre-test: 13.16 (2.41); Post-test: 15.63 (1.80)<br>**Social stimulation**<br>Pre-test: 12.96 (4.28); Post-test: 11.92 (4.28)
Liu et al. (2011)	180 SSI	No<br>12 months	Baseline: 14.6 (3.3)<br>6 months: 15.19 (3.3)<br>12 months: 15.44 (4.1)
Penn and Roberts (2005)	7 SSI	Yes<br>3 months (open-trial of social cognition training)	Baseline = 12.1 (4.8)<br>Post-test = 15.3 (3.4)
Lysaker et al. (2011)	36 SCZ/SCZaff	No<br>6 months	Baseline = 11.89 (4.80)<br>6 months = 12.89 (4.40)
Mizrahi et al. (2007)	71 SSI	Yes,<br>6 weeks<br>antipsychotics<br>6 weeks	Baseline = 14.58 (3.1)<br>6 weeks = 16.82 (2.8)<br>t = -2.30, p = .034

４．妥当性

(Relationship to social cognitive measures relative to other abilities and constructs)

※この項目では、他の社会認知機能との関連が強い尺度が良いと考える。

Author and Date	Sample	Findings
Corcoran, Mercer, and	55 SCZ<br>30 HC	HC: IQ (r=.08, ns)<br>SCZ: IQ (r=.49*).

Frith (1995)		
Bertrand et al. (2007)	38 SSI (first episode)<br>25 HC	SSI: IQ (r = .46*)<br>HC: IQ (r = .18, ns)
Corcoran (2003)	39 SCZ<br>44 HC	HC: Hinting uncorrelated with IQ and means ends social problem solving<br>SCZ: Means-ends problem solving (r=.38*); IQ (.29, ns).
Krabbendam, Jolles, and Van Os (2003)	43 SCZ/SCZaff<br>41 first degree relatives<br>43 HC	Hinting task was associated with the false belief task (OR = 1.43, 95% CI 0.45–4.56).
Corcoran and Frith (2003)	59 SCZ<br>44 HC	Hinting task and ToM stories (.63, $p < .05$)
Greig et al. (2004)	128 SCZ/SCZaff	Pearson correlations:<br>Verbal memory (.42*); Figure memory (.28*), executive functioning (.34*), Global IQ (.31*).
Bora et al. (2006)	50 SCZ	Pearson correlations:<br>Eyes (.51*) (note: correlation attenuated when accounting for neurocognitive index).<br>Auditory consonant trigrams (.61*), IQ (.54*); trails B (-.44*)
Schenkel et al. (2005)	42 SCZ/SCZaff	Pearson correlations:<br>Goodness of fit context task (.47*); Contour task (.43*).<br>Executive functioning, verbal fluency, IQ (ns).
van Hooren et al. (2008)	186 psychosis vulnerability<br>44 SSI<br>47 familial risk<br>41 psychometric risk<br>54 HC	Hinting uncorrelated with speech attribution task, beads task and internal, personal, situational attributions task (IPSAQ).<br>Hinting significantly correlated with the following neurocognitive measures: SCWT-speed (.35), SCWT (.31), TMT-speed (.33), TMT (.30), semantic fluency (.44) (all significant to $p<.01$).
Rubio et al. (2011)	42 SSI<br>21 siblings<br>77 HC	Hinting and Draws to Decisions (jumping to conclusions task) (r=-.389*)
Menon, Addington, and Remington (2011)	18 SCZ/SCZaff	Hinting and social variant of probabilistic reasoning (-.35*).<br>Hinting uncorrelated with IPSAQ.
Choi, Liu, and	36 SSI	Hinting uncorrelated with emotion context processing scale

Spalding (2012)		
Uhlhaas et al. (2006)	48 SCZ/SCZaff<br>26 HC<br>5 mood disorder<br>10 substance use<br>11 personality disorders	HC: visual size performance task (r = -.35*)<br>SCZ/SCZaff: (r = -.33*)
Lysaker et al. (2011)	36 SCZ/SCZaff	Hinting and Bell Lysaker Emotion Recognition Test (BLERT) (r=.52*)<br>Hinting and Eyes test (r=.47*).
McGlade et al. (2008)	73 SCZ/SCZaff<br>78 HC	Eyes task (r=.38*).<br>Working memory (p < .05); IQ and spatial memory (ns).
Bell et al. (2010)	66 SCZ/SCZaff<br>85 HC	Social attributions task (r=.37*) across both samples
Bell et al. (2008)	151 SCZ/SCZaff	BLERT total (r = .17*)<br>Proverbs total (r = .45*); Verbal learning (r=.25*), Wisconsin card sorting test (WCST) (.26*), digit span (r=.23*).
Bell et al. (2011)	77 SCZ/SCZaff	MSCEIT managing emotions branch (r=.22*); social attribution task (r=.14, ns).
Fanning et al. (2012)	119 SCZ/SCZaff	MATRICS battery correlations:<br>processing speed (r=.39, p<.001), working memory (r=.24, p<.01), verbal learning (r=.23, p<.05), and composite neurocognition (r=.28, p<.01).
Couture et al. (2011)	178 SCZ/SCZaff	Hinting was significantly correlated with neurocognition (r=.516, p<.01)

(Correlations with Real-World Social Outcomes)

Author and Date	Sample	Findings
Bora et al. (2006)	50 SCZ	Social functioning Scale total (r=.43*); after controlling for cognitive functioning: (r=.24, ns).
Schenkel et al. (2005)	42 SCZ/SCZaff	Poorer performance on the Hinting Task was associated with poor premorbid social functioning rated from medical charts (social history interview) (t(40) = 3.86, p < 0.0001).
Liu et al. (2011)	180 SSI (inpatients)	Hinting task not correlated with social functioning on the unit as measured by the NOSIE.
Silverstein, Wallace, and Schenkel (2005)	26 SCZ	Micro-module learning test (MMLT) (r=.77*).

=

McGlade et al. (2008)	73 SCZ/SCZaff 78 HC	Independent Living Scale (ILS) was not significantly associated with Hinting task performance
Couture et al. (2011)	178 SCZ/SCZaff	Social competence (role play) (r=.41*); self-reported functioning (r=.17*).
Bell et al. (2008)	151 SCZ/SCZaff	Work behavior inventory (r = .355*).

文献

Retrieved 8/23/2012 from Web of Knowledge

ORIGINAL CITATION:

Title: SCHIZOPHRENIA, SYMPTOMATOLOGY AND SOCIAL INFERENCE - INVESTIGATING THEORY OF MIND IN PEOPLE WITH SCHIZOPHRENIA

Author(s): CORCORAN, R; MERCER, G; FRITH, CD

Source: SCHIZOPHRENIA RESEARCH Volume: 17 Issue: 1 Pages: 5-13 DOI: 10.1016/0920-9964(95)00024-G Published: SEP 1995

Times Cited: 279 (from All Databases)

CITING ARTICLES

Title: Social cognitive impairments in first episode psychosis

Author: Marie-Claude Bertrand, Hazel Sutton, Amélie M. Achim, Ashok K. Malla, Martin Lepage

Source: Schizophrenia Research - September 2007 (Vol. 95, Issue 1, Pages 124-133, DOI: 10.1016/j.schres.2007.05.033)

Title: Inductive reasoning and the understanding of intention in schizophrenia.

Author(s): Corcoran, Rhiannon

Source: Cognitive neuropsychiatry Volume: 8 Issue: 3 Pages: 223-35 DOI: 10.1080/13546800244000319 Published: 2003-Aug

Times Cited: 13 (from All Databases)

Title: Deficits of social-cognitive and social-perceptual aspects of theory of mind in remitted patients with schizophrenia - Effect of residual symptoms

Author(s): Bora, Emre; Gokcen, Sezen; Kayahan, Buelent; et al.

Source: JOURNAL OF NERVOUS AND MENTAL DISEASE Volume: 196 Issue: 2 Pages: 95-99 DOI: 10.1097/NMD.0b013e318162a9e1 Published: FEB 2008

Times Cited: 15 (from All Databases)

Title: Alterations in theory of mind in patients with schizophrenia and non-psychotic relatives

Author(s): Janssen, I; Krabbendam, L; Jolles, J; et al.

Source: ACTA PSYCHIATRICA SCANDINAVICA Volume: 108 Issue: 2 Pages: 110-117 DOI: 10.1034/j.1600-0447.2003.00092.x Published: AUG 2003

Times Cited: 89 (from All Databases)

Title: Autobiographical memory and theory of mind: evidence of a relationship in schizophrenia
Author(s): Corcoran, R; Frith, CD
Source: PSYCHOLOGICAL MEDICINE Volume: 33 Issue: 5 Pages: 897-905 DOI: 10.1017/S0033291703007529 Published: JUL 2003
Times Cited: 68 (from All Databases)

Title: Theory of mind performance in schizophrenia: Diagnostic, symptom, and neuropsychological correlates
Author(s): Greig, TC; Bryson, GJ; Bell, MD
Source: JOURNAL OF NERVOUS AND MENTAL DISEASE Volume: 192 Issue: 1 Pages: 12-18 DOI: 10.1097/01.nmd.0000105995.67947.fc Published: JAN 2004
Times Cited: 80 (from All Databases)

Title: Social cognition and interaction training (SCIT) for outpatients with schizophrenia: A preliminary study
Author(s): Roberts, David L.; Penn, David L.
Source: PSYCHIATRY RESEARCH Volume: 166 Issue: 2-3 Pages: 141-147 DOI: 10.1016/j.psychres.2008.02.007 Published: APR 30 2009
Times Cited: 23 (from All Databases)

Title: Social functioning, theory of mind and neurocognition in outpatients with schizophrenia; mental state decoding may be a better predictor of social functioning than mental state reasoning
Author(s): Bora, Emre; Eryavuz, Ayse; Kayahan, Bulent; et al.
Source: PSYCHIATRY RESEARCH Volume: 145 Issue: 2-3 Pages: 95-103 DOI: 10.1016/j.psychres.2005.11.003 Published: DEC 7 2006
Times Cited: 72 (from All Databases)

Lindsay S. Schenkel, William D. Spaulding, Steven M. Silverstein, Poor premorbid social functioning and theory of mind deficit in schizophrenia: evidence of reduced context processing?, Journal of Psychiatric Research, Volume 39, Issue 5, September 2005, Pages 499-508, ISSN 0022-3956, 10.1016/j.jpsychires.2005.01.001.

Title: Impact of family involvement on social cognition training in clinically stable outpatients with schizophrenia - A randomized pilot study
Author(s): Tas, Cumhur; Danaci, Aysen E.; Cubukcuoglu, Zeynep; et al.
Source: PSYCHIATRY RESEARCH Volume: 195 Issue: 1-2 Pages: 32-38 DOI: 10.1016/j.psychres.2011.07.031 Published: JAN 30 2012
Times Cited: 1 (from All Databases)

Title: Persecutory beliefs, attributions and theory of mind: comparison of patients with paranoid delusions, Asperger's syndrome and healthy controls
Author(s): Craig, JS; Hatton, C; Craig, FB; et al.
Source: SCHIZOPHRENIA RESEARCH Volume: 69 Issue: 1 Pages: 29-33 DOI: 10.1016/S0920-9964(03)00154-3 Published: JUL 1 2004
Times Cited: 65 (from All Databases)

Title: Social cognition and neurocognition as independent domains in psychosis

Author(s): van Hooren, S.; Versmissen, D.; Janssen, I.; et al.
Source: SCHIZOPHRENIA RESEARCH Volume: 103 Issue: 1-3 Pages: 257-265 DOI: 10.1016/j.schres.2008.02.022 Published: AUG 2008
Times Cited: 24 (from All Databases)

Heterogeneity and the Longitudinal Recovery of Functioning During Inpatient Psychiatric Rehabilitation for Treatment-Refractory Severe Mental Illness
Nancy H. Liu, Kee-Hong Choi, Felice Reddy, William D. Spaulding American Journal of Psychiatric Rehabilitation Vol. 14, Iss. 1, 2011

Title: Jumping to conclusions in psychosis: A faulty appraisal
Author(s): Luis Rubio, Jose; Ruiz-Veguilla, Miguel; Hernandez, Laureno; et al.
Source: SCHIZOPHRENIA RESEARCH Volume: 133 Issue: 1-3 Pages: 199-204 DOI: 10.1016/j.schres.2011.08.008 Published: DEC 2011
Times Cited: 0 (from All Databases)

Title: Social Cognitive Impairments and Psychotic Symptoms: What is the Nature of Their Association?
Author: Anne-Kathrin J. Fett, Arija Maat, and GROUP Investigators
Source: Schizophr Bull first published online June 22, 2011 doi:10.1093/schbul/sbr058

Title: Is it possible to have impaired neurocognition but good social cognition in schizophrenia?
Author: Fanning JR, Bell MD, Fiszdon JM.
Source: Schizophr Res. 2012 Mar;135(1-3):68-71. Epub 2012 Jan 14.

Title: Pilot study of social cognition and interaction training (SCIT) for schizophrenia
Author(s): Penn, D; Roberts, DL; Munt, ED; et al.
Source: SCHIZOPHRENIA RESEARCH Volume: 80 Issue: 2-3 Pages: 357-359 DOI: 10.1016/j.schres.2005.07.011 Published: DEC 15 2005
Times Cited: 32 (from All Databases)

Title: Poor insight in schizophrenia: links between different forms of metacognition with awareness of symptoms, treatment need, and consequences of illness
Author(s): Lysaker, Paul H.; Dimaggio, Giancarlo; Buck, Kelly D.; et al.
Source: COMPREHENSIVE PSYCHIATRY Volume: 52 Issue: 3 Pages: 253-260 DOI: 10.1016/j.comppsych.2010.07.007 Published: MAY-JUN 2011
Times Cited: 7 (from All Databases)

M. Menon, J. Addington, G. Remington, Examining cognitive biases in patients with delusions of reference, European Psychiatry, Available online 11 June 2011, ISSN 0924-9338, 10.1016/j.eurpsy.2011.03.005.

Title: Cognitive alterations in patients with non-affective psychotic disorder and their unaffected siblings and parents
Author(s): Meijer, J.; Simons, C. J. P.; Quee, P. J.; et al.
Group Author(s): GRP Investigators
Source: ACTA PSYCHIATRICA SCANDINAVICA Volume: 125 Issue: 1 Pages: 66-76 DOI: 10.1111/j.1600-0447.2011.01777.x Published: JAN 2012

Times Cited: 2 (from All Databases)

Emotional context processing in severe mental illness: Scale development and preliminary construct validity

Kee-Hong Choi; Nancy Liu; Will Spaulding (Profiled Author: William D Spaulding)

Psychiatry Research 2012;():.

Title: Theory of mind and perceptual context-processing in schizophrenia.

Author(s): Uhlhaas, Peter J; Phillips, William A; Schenkel, Lindsay S; et al.

Source: Cognitive neuropsychiatry Volume: 11 Issue: 4 Pages: 416-36 DOI: 10.1080/13546800444000272

Published: 2006-Jul

Times Cited: 20 (from All Databases)

Title: The micro-module learning tests: Work-sample assessments of responsiveness to skills training

Author(s): Silverstein, SM; Wallace, CJ; Schenkel, LS

Source: SCHIZOPHRENIA BULLETIN Volume: 31 Issue: 1 Pages: 73-83 DOI: 10.1093/schbul/sbi008

Published: JAN 2005

Times Cited: 6 (from All Databases)

Title: The development of the social cognition and interaction training program for schizophrenia spectrum disorders

Author(s): Penn, David L.; Roberts, David L.; Combs, Dennis; et al.

Source: PSYCHIATRIC SERVICES Volume: 58 Issue: 4 Pages: 449-451 DOI: 10.1176/appi.ps.58.4.449

Published: APR 2007

Times Cited: 38 (from All Databases)

Title: The effect of antipsychotic treatment on theory of mind

Author(s): Korostil, M.; Mizrahi, R.; Starkstein, S.; et al.

Conference: 10th International Congress on Schizophrenia Research Location: Savannah, GA Date: APR 02-06, 2005

Source: SCHIZOPHRENIA BULLETIN Volume: 33 Issue: 2 Pages: 564-565 Published: MAR 2007

Times Cited: 0 (from All Databases)

Title: Alterations in theory of mind in patients with schizophrenia and non-psychotic relatives

Author(s): Janssen, I; Krabbendam, L; Jolles, J; et al.

Source: ACTA PSYCHIATRICA SCANDINAVICA Volume: 108 Issue: 2 Pages: 110-117 DOI: 10.1034/j.1600-0447.2003.00092.x Published: AUG 2003

Times Cited: 89 (from All Databases)

Title: Symptomatology and social inference: a theory of mind study of schizophrenia and psychotic affective disorder.

Author(s): Marjoram, Dominic; Gardner, Clare; Burns, Jonathan; et al.

Source: Cognitive neuropsychiatry Volume: 10 Issue: 5 Pages: 347-59 DOI: 10.1080/13546800444000092

Published: 2005-Nov

Times Cited: 22 (from All Databases)

Title: Metacognition in schizophrenia: Correlates and stability of deficits in theory of mind and self-

reflectivity
Author(s): Lysaker, Paul H.; Olesek, Kyle L.; Warman, Debbie M.; et al.
Source: PSYCHIATRY RESEARCH Volume: 190 Issue: 1 Pages: 18-22 DOI: 10.1016/j.psychres.2010.07.016 Published: NOV 30 2011
Times Cited: 6 (from All Databases)
Title: Mental state decoding v. mental state reasoning as a mediator between cognitive and social function in psychosis
Author(s): McGlade, Nicola; Behan, Caragh; Hayden, Judy; et al.
Source: BRITISH JOURNAL OF PSYCHIATRY Volume: 193 Issue: 1 Pages: 77-78 DOI: 10.1192/bjp.bp.107.044198 Published: JUL 2008
Times Cited: 18 (from All Databases)
Title: Social attribution test - multiple choice (SAT-MC) in schizophrenia: Comparison with community sample and relationship to neurocognitive, social cognitive and symptom measures
Author(s): Bell, Morris D.; Fiszdon, Joanna M.; Greig, Tamasine C.; et al.
Source: SCHIZOPHRENIA RESEARCH Volume: 122 Issue: 1-3 Pages: 164-171 DOI: 10.1016/j.schres.2010.03.024 Published: SEP 2010
Times Cited: 2 (from All Databases)
Title: A path model investigation of neurocognition, theory of mind, social competence, negative symptoms and real-world functioning in schizophrenia
Author(s): Couture, Shannon M.; Granholm, Eric L.; Fish, Scott C.
Source: SCHIZOPHRENIA RESEARCH Volume: 125 Issue: 2-3 Pages: 152-160 DOI: 10.1016/j.schres.2010.09.020 Published: FEB 2011
Times Cited: 7 (from All Databases)
Title: Neurocognition, Social Cognition, Perceived Social Discomfort, and Vocational Outcomes in Schizophrenia
Author(s): Bell, Morris; Tsang, Hector W. H.; Greig, Tamasine C.; et al.
Source: SCHIZOPHRENIA BULLETIN Volume: 35 Issue: 4 Pages: 738-747 DOI: 10.1093/schbul/sbm169 Published: JUL 2009
Times Cited: 39 (from All Databases)
Title: Social Cognitive Impairments and Negative Symptoms in Schizophrenia: Are There Subtypes With Distinct Functional Correlates?
Author: Morris D. Bell, Silvia Corbera, Jason K. Johannesen, Joanna M. Fiszdon, and Bruce E. Wexler

Source: Schizophr Bull first published online October 5, 2011 doi:10.1093/schbul/sbr125
Morris D. Bell, Aaron L. Mishara, Does negative symptom change relate to neurocognitive change in schizophrenia? Implications for targeted treatments, Schizophrenia Research, Volume 81, Issue 1, 1 January 2006, Pages 17-27, ISSN 0920-9964, 10.1016/j.schres.2005.09.016.

4. Reading the Mind in the Eyes Test

1. 実用可能性と忍容性

(Practicality for Administration and Tolerability for Patients)

Author and Date	Sample	Findings
Bora et al. (2006)	50 SCZ	36 items, multiple choice, requires no informant rating

Notes: The above is the standard stimulus set. It is typically administered on a PC or laptop, is quick to score, and takes about 25-30 minutes.

２. 信頼性

(Test-Retest, Internal Consistency, Inter-rater Reliability as applicable)

Author and Date	Sample	Findings
Hallerback et al. (2009)	158 CS	Pearson's r between initial and follow up testing (3 weeks) = .60, p < .01
Yildirim et al. (2011)	130 HC	Test-retest (2 weeks) = .650, p < .01, two items (19 and 21 were found to be unreliable upon test-retest)

３. 有用性

(Floor and Ceiling Effects, Normality of Distributions)

Author and Date	Sample	Findings
Bora et al. (2006)	50 SCZ	SCZ (good outcome) = 16.2(0.6)<br>SCZ (poor outcome) = 12.7(0.6)<br>(only used first 27 items)
Irani et al. (2006)	10 SCZ<br>10 first degree relatives<br>10 HC	**Only figures provided**<br>HC did not answer more than 80% correctly
Couture et al. (2008)	26 young SSI<br>88 Clinical High Risk (CHR)<br>41 HC	SSI = 62.7%(13.7) correct<br>CHR = 69.6% (13.8) correct<br>HC = 68.8% (14.0) correct
Craig et al. (2004)	17 Aspergers Syndrome<br>16 HC<br>16 SSI	Aspergers: 19.88 (6.10)<br>SSI: 18.19 (6.65)<br>HC: 27.63 (4.33)
Hallerback et al. (2009)	158 CS	Scores were not normally distributed.<br>For all participants (out of 28)<br>Including 4 questionable items = 20.5(2.4)

		W/o questionable items (out of 24) = 18.9 (2.1)<br>58 participants who took it twice-<br>1st test (with questionable items)- 20.7(2.5), 2nd test-19.2(2.2)<br>1st test (with questionable items- 20.9(2.8), 2nd test-18.9(2.6)
Baron-Cohen et al. (2001)	15 HFA or Aspergers<br>122 HC<br>103 CS	HFA/AS = 21.9 (6.6)<br>HC = 26.2 (3.6)<br>CS = 28.0 (3.5)
Kettle et al. (2008)	27 CS<br>16 HC<br>13 SCZ<br>14 depressed	No means provided in the text; only a scatterplot. Pattern of performance:<br>CS=HC>depressed=SCZ
Fertuck et al. (2009)	30 borderline PD<br>25 HC	BPD= 28.5(3.3) out of 36<br>HC= 25(3.63) out of 36
Kelemen et al. (2005)	52 SCZ<br>30 HC (split into remitted and non-remitted)	HC = 22.5 (2.9)<br>SCZ (remitted) = 19.6 (4.5)<br>SCZ (non-remitted) = 17.9 (5.4)
Kelemen et al. (2004)	40 HC<br>79 first degree relatives of people with SCZ (split into ―affected‖ and ―unaffected‖ relatives)	HC = 27.8 (5.0)<br>Relatives (unaffected) = 28.6 (5.2)<br>Relatives (affected) = 21.5 (4.4)
Russell et al. (2000)	5 SCZ<br>7 HC	SCZ = 12.6 (5.03) errors<br>HC = 6.14 (3.84) errors
Shaw et al. (2004)	53 HC	HC = 27.6/36(4)
Bora et al. (2007)	58 SCZ	SCZ=18.16(4.68) (out of 36).
Domes et al. (2007)	30 HC	69.4% correct (8.1)

(Utility as a Repeated Measure)

※この項目では、自然経過では不変で介入によってより大きく動く尺度が良いと考える。

Author and Date	Sample	Treatment study?	Findings

		Testing interval	
Domes et al. (2007)	30 HC	Yes<br>No interval (immediate check on ToM after administration of intranasal oxytocin).	Placebo: 69.4 +/- 8.1; oxytocin: 72.4 +/- 8.6; t = 2.18, df = 29, p = .019
Tas et al. (2012)	52 SCZ	Yes<br>14 weeks<br>-SCIT versus social stimulation	**SCIT**<br>Pre-test: 20.26 (4.12); Post-test: 20.16 (4.32)<br>**Social stimulation**<br>Pre-test: 18.27 (4.85); Post-test: 17.45 (5.40)

４．妥当性

(Relationship to social cognitive measures relative to other abilities and constructs)

※この項目では、他の社会認知機能との関連が強い尺度が良いと考える。

Author and Date	Sample	Findings
Bora et al. (2006)	50 SCZ	**Pearson correlations (* < .05)**:<br>-Hinting task (.51*)<br>-Auditory Consonant Trigrams (.47*)<br>-WAIS information (.25) (ns)
Craig et al. (2004)	17 Aspergers Syndrome<br>16 HC<br>16 SSI	Eyes and Hinting task (r = .54, p < .01)
Baron-Cohen et al. (2001)	15 HFA/Aspergers Syndrome<br>103 CS<br>14 HC	Eyes and autism quotient (r = -.53, p = .004)<br>Eyes and IQ (r = .09, ns)
Uhlhaas et al. (2006)	48 SCZ/SCZaff<br>26 HC<br>5 mood disorder<br>10 substance use<br>11 personality disorders	Eyes and visual size perception task (r=.10, ns)
Kelemen et al. (2005)	52 SCZ<br>30 HC	Eyes uncorrelated with IQ

Kelemen et al. (2004)	14 affected relatives<br>65 unaffected first degree relatives of people with schizophrenia	Eyes uncorrelated with IQ
Shaw et al. (2004)	53 HC	Eyes test and verbal IQ :<br>(n=48, r=0.385, p=0.07)
Bora et al. (2007)	58 SCZ	Pearson correlations<br>First order ToM (.24, ns); Second order ToM (.40*)<br>Digit span forward (.41*), Digit span backward (.44*), letter to number (.44*); WCST and verbal fluency (ns)

(Correlations with Real-World Social Outcomes)

Author and Date	Sample	Findings
Bora et al. (2006)	50 SCZ	beta = .34, p = .03 with Social Functioning Scale (SFS) total; beta = .36 p = .01 for SFS interpersonal, beta = .40 (p = .001) for SFS social activities, and beta = .30 p = .05

文献

ORIGINAL CITATION:

Title: The "Reading the Mind in the Eyes" test revised version: A study with normal adults, and adults with Asperger syndrome or high-functioning autism

Author(s): Baron-Cohen, S ; Wheelwright, S ; Hill, J ; et al.

Source: JOURNAL OF CHILD PSYCHOLOGY AND PSYCHIATRY AND ALLIED DISCIPLINES Volume: 42 Issue: 2 Pages: 241-251 DOI: 10.1017/S0021963001006643 Published: FEB 2001

Times Cited: 404

CITING ARTICLES

Title: Theory of mind and unawareness of illness in schizophrenia - Is poor insight a mentalizing deficit?

Author(s): Bora, E (Bora, Emre); Sehitoglu, G (Sehitoglu, Gulsah); Aslier, M (Aslier, Mustafa); Atabay, I

(Atabay, Ihsan); Veznedaroglu, B (Veznedaroglu, Baybars)

Source: EUROPEAN ARCHIVES OF PSYCHIATRY AND CLINICAL NEUROSCIENCE Volume: 257

Published: 2007

Title: Social functioning, theory of mind and neurocognition in outpatients with schizophrenia; mental state

decoding may be a better predictor of social functioning than mental state reasoning
Author(s): Bora, E (Bora, Emre); Eryavuz, A (Eryavuz, Ayse); Kayahan, B (Kayahan, Bulent); Sungu, G
(Sungu, Gulcin); Veznedaroglu, B (Veznedaroglu, Baybars)
Source: PSYCHIATRY RESEARCH Volume: 145 Issue: 2-3 Pages: 95-103 Published: 2006
Title: Assessment of social judgments and complex mental states in the early phases of psychosis
Author(s): Couture, SM (Couture, Shannon M.); Penn, DL (Penn, David L.); Addington, J (Addington, Jean);
Woods, SW (Woods, Scott W.); Perkins, DO (Perkins, Diana O.)
Source: SCHIZOPHRENIA RESEARCH Volume: 100 Issue: 1-3 Pages: 237-241 Published: 2008
Title: Persecutory beliefs, attributions and theory of mind: comparison of patients with paranoid delusions,
Asperger's syndrome and healthy controls
Author(s): Craig, JS (Craig, JS); Hatton, C (Hatton, C); Craig, FB (Craig, FB); Bentall, RP (Bentall, RP)
Source: SCHIZOPHRENIA RESEARCH Volume: 69 Issue: 1 Pages: 29-33 Published: 2004
Title: Oxytocin improves "mind-reading" in humans
Author(s): Domes, G (Domes, Gregor); Heinrichs, M (Heinrichs, Markus); Michel, A (Michel, Andre);
Berger, C (Berger, Christoph); Herpertz, SC (Herpertz, Sabine C.)
- 142 -
Source: BIOLOGICAL PSYCHIATRY Volume: 61 Issue: 6 Pages: 731-733 Published: 2007
Title: Enhanced 'Reading the Mind in the Eyes' in borderline personality disorder compared to healthy controls
Author(s): Fertuck, EA (Fertuck, E. A.); Jekal, A (Jekal, A.); Song, I (Song, I.); Wyman, B (Wyman, B.);
Morris, MC (Morris, M. C.); Wilson, ST (Wilson, S. T.); Brodsky, BS (Brodsky, B. S.); Stanley, B (Stanley, B.)
Source: PSYCHOLOGICAL MEDICINE Volume: 39 Issue: 12 Published: 2009
Title: The Reading the Mind in the Eyes Test: test-retest reliability of a Swedish version.
Author(s): Hallerback, Maria Unenge; Lugnegard, Tove; Hjarthag, Fredrik; et al.
Source: Cognitive neuropsychiatry Volume: 14 Issue: 2 Pages: 127-43 Published: 2009-Mar
Title: Self-face recognition and theory of mind in patients with schizophrenia and first-degree relatives
Author(s): Irani, F (Irani, Farzin); Platek, SM (Platek, Steven M.); Panyavin, IS (Panyavin, Ivan S.); Calkins, ME
(Calkins, Monica E.); Kohler, C (Kohler, Christian); Siegel, SJ (Siegel, Steven J.); Schachter, M

(Schachter, Michael); Gur, RE (Gur, Raquel E.); Gur, RC (Gur, Ruben C.)
Source: SCHIZOPHRENIA RESEARCH Published: DEC 2006
Title: Impaired theory of mind in first-episode schizophrenia: comparison with community, university and
depressed controls
Author(s): Kettle, JWL (Kettle, Jonathan W. L.); O'Brien-Simpson, L (O'Brien-Simpson, Laurie); Allen, NB
(Allen, Nicholas B.)
Source: SCHIZOPHRENIA RESEARCH Volume: 99 Issue: 1-3 Published: FEB 2008
Title: Theory of mind and motion perception in schizophrenia
Author(s): Kelemen, O (Kelemen, O); Erdelyi, R (Erdelyi, R); Pataki, I (Pataki, I); Benedek, G (Benedek, G);
Janka, Z (Janka, Z); Keri, S (Keri, S)
Source: NEUROPSYCHOLOGY Volume: 19 Published: JUL 2005
Title: No evidence for impaired 'theory of mind' in unaffected first-degree relatives of schizophrenia patients
Author(s): Kelemen, O (Kelemen, O); Keri, S (Keri, S); Must, A (Must, A); Benedek, G (Benedek, G);
Janka, Z (Janka, Z)
Source: ACTA PSYCHIATRICA SCANDINAVICA Volume: 110 Published: AUG 2004
Title: Measuring empathy: reliability and validity of the Empathy Quotient
Author(s): Lawrence, EJ (Lawrence, EJ); Shaw, P (Shaw, P); Baker, D (Baker, D); Baron-Cohen, S
(Baron-Cohen, S); David, AS (David, AS)

Source: PSYCHOLOGICAL MEDICINE Volume: 34 Published: JUL 2004
Title: Exploring the social drain in schizophrenia: Left prefrontal underactivation during mental state attribution Author(s): Russell, TA; Rubia, K; Bullmore, ET; et al. Source: AMERICAN JOURNAL OF PSYCHIATRY Published: DEC 2000
Title: [Reading mind from pictures of eyes: theory of mind, language ability, general intellectual ability, and autism]. Author(s): Senju, Atsushi; Tojo, Yoshikuni; Konno, Michiko; et al. Source: Shinrigaku kenkyu : The Japanese journal of psychology Published: 2002-Apr
Title: Theory of mind and perceptual context-processing in schizophrenia.
Author(s): Uhlhaas, Peter J; Phillips, William A; Schenkel, Lindsay S; et al.Source: Cognitive neuropsychiatry Volume: 11 Issue: 4 Published: 2006-Jul
Title: Investigation of the Reliability of the "Reading the Mind in the Eyes Test" in a Turkish Population

4. Eyes

Author(s): Yildirim, EA (Yildirim, Ejder Akgun); Kasar, M (Kasar, Muzaffer); Guduk, M (Guduk, Mehmet);
Ates, E (Ates, Elif); Kucukparlak, I (Kucukparlak, Ilker); Ozalmete, EO (Ozalmete, Erdem Onur)
Source: TURK PSIKIYATRI DERGISI Published: 2011

5. Intentional Bias Task（IBT）

1. 実用可能性と忍容性

(Practicality for Administration and Tolerability for Patients)

Author and Date	Sample	Notes

２. 信頼性

(Test-Retest, Internal Consistency, Inter-rater Reliability as applicable)

Author and Date	Sample	Findings

３. 有用性

(Floor and Ceiling Effects, Normality of Distributions)

Author and Date	Sample	Findings
Slavny et al., 2018	59 participants aged 15–42 years (M = 22.95, SD = 6.08) with 38 females	The mean intentionality bias score was 65.95% (SD = 23.00) for the Neutral/Prototypically Intentional sentences and 20.12% (SD = 11.87) for Prototypically Accidental test sentences. There were no significant gender differences in the intentionality bias scores in the Prototypically Accidental (Mann-Whitney U = 292.0, p = 0.378) or Neutral/Prototypically Intentional condition (Mann-Whitney U = 268.5, p = 0.194).
Brotherton et el., 2015	Study 2: 102 first-year psychology undergraduate students<br>Study 3: 86 psychology students	(Modified version; 12 ambiguous sentences selected, no time constraints, score range 1-12)<br>Study 2: Participants tended to offer intentional attributions for significantly more than half (M = 7.47; SD = 1.52) of the 12 items (t (85) = 9.14, p <. 001, d = 0.99). The data were approximately normally distributed about the mean, with slight negative skew; scores ranged from 4 to 10 (median = 7; skew = -.14).<br>Study 3: Participants generally interpreted significantly more than half (M = 7.75, SD = 2.41) of the ambiguous sentences

		as being intentional actions (t (85) = 6.70, p <. 001, d = 0.72).
Begue et al., 2010	80 French men, 2 X 2 balanced placebo design, participant received either a high dose of alcohol (target BAC=.10%) or no alcohol, with half of each group believing they had or had not consumed alcohol.	(20 ambiguous sentences)

Table 1. Intentionality as a Function of Alcohol Consumption and Alcohol Expected

	Expected					
	Alcohol		No alcohol		All	
	M	*SD*	*M*	*SD*	*M*	*SD*
Alcohol	0.40	0.14	0.45	0.17	0.43	0.15
No alcohol	0.40	0.17	0.32	0.14	0.36	0.15

(Utility as a Repeated Measure)

※この項目では、自然経過では不変で介入によってより大きく動く尺度が良いと考える。

Author and Date	Sample	Treatment Study? Testing Interval	Findings

4．妥当性

(Relationship to social cognitive measures relative to other abilities and constructs)

※この項目では、他の社会認知機能との関連が強い尺度が良いと考える。

Author and Date	Sample	Findings
Slavny et al., 2018	59 participants aged 15–42 years (M = 22.95, SD = 6.08) with 38 females	Relationship to Questionnaire of Cognitive and Affective Empathy (QCAE)<br>・ Prototypically Accidental condition: Cognitive empathy subset significantly predicted intentionality bias scores (β = 0.40; p = 0.012), while the independent prediction of affective empathy subset was non-significant (β = − 0.01; p = 0.941). Among cognitive empathy components, perspective taking (β = 0.41; p = 0.008), but not online simulation subset (β = 0.02; p = 0.885), independently predicting

		intentionality bias scores.<br>・Neutral/Prototypically Intentional condition: Neither cognitive empathy (β = 0.19; p = 0.239) or affective empathy (β = 0.11; p = 0.511) subsets significantly predicted intentionality bias scores.
Brotherton et el., 2015	Study 2: 102 first-year psychology undergraduate students<br>Study 3: 86 psychology students	(Modified version; 12 ambiguous sentences selected, no time constraints, score range 1-12)<br>Study 2: There was a small but statistically significant positive correlation between GCB (generic conspiracy beliefs) scores and the number of intentional inferences participants offered ($r(84) = .22$, $p < .05$).<br>Study 3: There was no significant association between intentional inferences and anthropomorphism ($r(84) = -.10$, $p = .37$).
Hughes et al., 2012	35 female, 18 male healthy students	(74 action sentences drawn from Rosset, 2018 study 1)<br>2 (Condition: speeded vs. unspeeded)×4(SentenceType: accidental control vs. intentional control vs. prototypically intentional vs.prototypically accidental) mixed factorial:<br>・No effect of condition, $F(1, 51) = 2.49$, $p = .12$, $\eta p2 = 0.05$.<br>・Main effect of Sentence Type, $F(3, 153) = 700.78$, $p < .001$, $\eta p2 = 0.93$; intentional control sentences ($M = 0.95$, $SD = 0.06$) > prototypically intentional sentences ($M = 0.63$, $SD = 0.15$) > prototypically accidental sentences ($M = 0.24$, $SD = 0.13$) > accidental control sentences ($M = 0.11$, $SD = 0.12$).<br>・Interaction between Condition and Sentence Type emerged, $F(3, 153) = 7.86$, $p < .001$, $\eta p2 = 0.13$;<br>accidental control sentences: speeded scores ($M = 0.16$, $SD = 0.13$) > unspeeded ($M = 0.05$, $SD = 0.05$), $t(51) = -4.15$, $p < .001$, $d = 1.16$.<br>intentional control sentences: unspeeded scores ($M = 0.99$, $SD = 0.02$) > speeded ($M = 0. 91$, $SD = 0.06$), $t(51) = 5.24$, $p < .001$, $d = 1.47$.<br>・No difference in the speeded (prototypically accidental: $M = 0.27$, $SD = 0.16$; prototypically intentional: $M = 0.63$, $SD = 0.16$) versus unspeeded conditions (prototypically accidental: $M = 0.20$, $SD = 0.09$; prototypically intentional: $M = 0.62$, $SD = 0.14$), $t(51) = -1.82$, $p = .07$, $d = 0.51$ and $t(51) = -0.34$, $p = .73$, $d = 0.20$, for prototypically accidental and intentional sentences respectively.<br>・Collapsed across test sentence type, scores were significantly different in the speeded ($M = 0.39$, $SD = 0.14$) versus unspeeded ($M = 0.35$, $SD = 0.09$) conditions, $t(51) = -2.05$, $p = .045$, $d = 0.57$.

		・RTs: intentional control sentences (M = 1,584, SD = 353) > accidental control sentences (M = 1,642, SD = 390), t(53) = 2.29, p = .02, d = 0.63. However, intentional test sentences (M = 1,954, SD = 603) < accidental test sentences (M = 1,836, SD = 514), t(53) = −3.08, p = .003, d = 0.85.<br>・Kuder Richardson coefficients revealed that participants were more consistent when responding to accidental control (KR-20 = 0.74) and accidental test sentences (KR-20 = 0.72) compared to intentional control and intentional test sentences (KR-20 = −0.04 and 0.08, respectively).
Begue et al., 2010	80 French men, 2 X 2 balanced placebo design, participant received either a high dose of alcohol (target BAC=.10%) or no alcohol, with half of each group believing they had or had not consumed alcohol.	(20 ambiguous sentences)

Table 2. Result of a 2 × 2 Between-Subjects ANOVA

Source	*SS*	*df*	*Ms*	*F*	*p*
Alcohol	0.09	1	0.09	3.75	.05
Expectancies	0.00	1	0.00	0.26	.60
Alcohol × expectancies	0.08	1	0.08	3.24	.07
Error	1.88	76	0.02		
Total	14.57	80			

(Correlations with Real-World Social Outcomes)

Author and Date	Sample	Findings

文献

ORIGINAL CITATION:

Title: It's no accident: Our bias for intentional explanations.

Author(s): Rosset, Evelyn

Source: COGNITION Volume: 108 Issue: 3 Pages: 771-780 DOI: 10.1016/j.cognition.2008.07.001 Published: 2008 Sep

Times Cited: 100 (from Web of Science)

CITING ARTICLES

Title: Individual differences in the intentionality bias and its association with cognitive empathy

Author(s): Slavny, Rachel J. M.; Moore, James W.

Source: PERSONALITY AND INDIVIDUAL DIFFERENCES Volume: 122 Pages: 104-108 DOI: 10.1016/j.paid.2017.10.010 Published: FEB 1 2018
Times Cited: 3 (from Web of Science)

Title: Intention Seekers: Conspiracist Ideation and Biased Attributions of Intentionality
Author(s): Brotherton, Robert; French, Christopher C.
Source: PLOS ONE Volume: 10 Issue: 5 Pages: UNSP e0124125 DOI: 10.1371/journal.pone.0124125 Published: May 13, 2015
Times Cited: 17 (from Web of Science)

Title: Intentional Inferences Are Not More Likely Than Unintentional Ones: Some Evidence Against the Intentionality Bias Hypothesis
Author(s): Hughes, Jamie S.; Sandry, Joshua; Trafimow, David
Source: JOURNAL OF SOCIAL PSYCHOLOGY Volume: 152 Issue: 1 Pages: 1-4 DOI: 10.1080/00224545.2011.565383 Published: 2012
Times Cited: 5 (from Web of Science)

Title: "There Is No Such Thing as an Accident," Especially When People Are Drunk
Author(s): Begue, Laurent; Bushman, Brad J.; Giancola, Peter R.; et al.
Source: PERSONALITY AND SOCIAL PSYCHOLOGY BULLETIN Volume: 36 Issue: 10 Pages: 1301-1304 DOI: 10.1177/0146167210383044 Published: OCT 2010
Times Cited: 15 (from Web of Science)

6. The Awareness of Social Inference Test (TASIT)

1. 実用可能性と忍容性

(Practicality for Administration and Tolerability for Patients)

Author and Date	Sample	Findings
McDonald et al. (2006)	70 chronic brain injury	Part 1: 28 vignettes with a professional actor (Requiring DVD), taking 15-20 minutes<br>Part 2: 15 vignettes of dialogues between two actors - administration takes 22-27 minutes, scoring of part 2 takes 5 minutes<br>Part 3: 16 vignettes takes 22-27 minutes to administer and scoring takes about 5 minutes
McDonald et al. (2004)	21 TBI	The three parts of TASIT had a combined playing time of approximately 35 minutes. Each subject was tested individually. It was explained to the subject that he/she would be shown a video of some people interacting and that he/she would be asked questions about these. Practice items preceded each section to familiarize the subject with the task requirements. The video was then paused after each vignette and the subject was asked to respond to questions concerning the content of the video.

２. 信頼性

(Test-Retest, Internal Consistency, Inter-rater Reliability as applicable)

Author and Date	Sample	Findings
McDonald et al. (2006)	32 adults with severe chronic brain injuries and 38 adults with brain injuries were administered alternate forms over a period of 5 to 26 weeks	Test retest reliability ranged from .74 to .88,<br>Alternate forms reliability ranged from .62 to .83

３. 有用性

(Floor and Ceiling Effects, Normality of Distributions)

Author and Date	Sample	Findings
McDonald et al. (2006)	32 adults with severe chronic brain injuries and 38 adults with brain	**Normative data**<br>**Part I:** FORM A = 24.86 out of 28 (SD = 2.11) FORM B = 24.15 (SD = 2.52) **Part II:** FORM A = 54.11 out of 60 (4.29), FORM B = 52.88 out of 60 (5.30) **Part III:** FORM A = 55.64 out of 64 (SD = 4.82), FORM B = 55.11 (5.28) out of 64

	injuries were administered alternate forms over a period of 5 to 26 weeks	**TBI participants**<br>**Part I:** FORM A (first) = 19.22 (5.06), FORM A (second) = 19.53 (4.72) Form B = 19.53 (4.72)<br>**Part II:** FORM A (first) = 44.13 (8.16), FORM A (second) = 44.52 (10.52) Form B = 40.59 (8.62)<br>**Part III:** FORM A (first) = 44.47(7.38), FORM A (second) = 43.94 (9.53)<br>Form B = 42.44 (8.09)
McDonald et al. (2004)	34 HC<br>34 traumatic brain injury (TBI)	**Controls**<br>**Part 1 = 25.5 (1.8) out of 28, Part 2 = 53.5 (4.1) out of 60, Part 3 = 54.7 (5.3) out of 64**<br>**TBI**<br>**Part 1 = 19.5 (4.5), Part 2 = 45.2 (9.1), Part 3 = 45.4 (7.9)**
McDonald et al. (2003)	169 HC and 7 adults with severe TBI (from pilot studies)<br>283 adults HC and 12 people with severe TBI studies (main studies)	**Part I: 95th percentile - 28/28, median 25/28, 5th percentile 20/28**<br>**Part I: FORM B 95th: 27/28, median 25/28, 5th percentile 17.8/28**<br>**Part II - 95th percentile - 60/60, median 55/60, 5th percentile 46/60**<br>**Part II FORM B 95th 60/60, median 54/60, 5th percentile 42/60**<br>**Part III: 95th percentile 62/64, median 56/64, 5 percentile 46/64**<br>**Part III: FORM B 95th percentile 62/64, median 56/64, 5 percentile 44.7/64**
McDonald et al. (2004b)	34 adults with severe TBI and 34 HC	**TBI: Part 1 = 19.6(4.7), Part 2 = 45.9(8.0), Part 3 = 53.1(4.2)**<br>**HC: Part 1 =25.0(2.3), Part 2 = 45.9(7.4), Part 3 = 53.9(6.0)**
Wynn et al. (2010)	33 SCZ<br>42 HC	**Part III only (out of 64)**<br>**SCZ = 47.82 (8.2); HC = 50.5 (6.2)**
Jahshan et al. (2007)	52 people high in schizotypy<br>40 people low in schizotypy	**Part I (upper limit = 28)**<br>**High schizotypy 23.5 (2.9), Low schizotypy 24.2 (2.1)**<br>**Part II (upper limit = 60)**<br>**High schizotypy 50.6 (5.0), Low schizotypy 50.8 (4.5)**<br>**Part III (upper limit = 64)**<br>**High schizotypy 53.2 (4.7), Low schizotypy 53.3 (4.0)**
Sparks et al. (2010)	25 HC<br>30 SCZaff	**TASIT Part I**<br>**SCZaff = 76% (18.8) correct; HC = 91.7% (6.5) correct**

	**TASIT Part II**<br>**SCZaff = 76% correct; HC = 93% correct total (SDs not provided)**<br>**TASIT Part III** SCZaff = 75% correct; HC = 90.7% correct total (SDs not provided)

(Utility as a Repeated Measure)

※この項目では、自然経過では不変で介入によってより大きく動く尺度が良いと考える。

Author and Date	Sample	Treatment Study?<br>Testing interval	Findings
McDonald et al. (2003)	452 HC<br>19 severe TBI	No<br>Ranged from a subset who took form B immediately after form A to a small subset who were able to delay their retest 6 months	**Testing for practice effects:**<br>Part I: 1 point increase, P = .012<br>Part II: 6 point increase, P = .008<br>Part II form B: 3 point increase, P = .013,<br>Part III 4.5 points increase, P = .000,<br>Part III form B: 5 points increment, P = .000).<br>There was no increment but rather a small and insignificant decrement when six subjects viewed form B of each test 6 months after form A, (EET: 2-point decrement, SI-M and SI-E, 1-point decrement), indicating that practice effects had well and truly dissipated by this time.
Roberts et al. (2009)	31 SSI	Yes<br>20 weeks<br>-Social Cognition and Interaction Training (SCIT)<br>-TAU	**Social cognition and interaction training**<br>Baseline: 26.30 (6.90)<br>Post-test: 29.50 (5.72)<br>**TAU**<br>Baseline: 27.38 (5.42)<br>Post-test: 27.50 (5.73)
McDonald et al. (2008)	51 TBI	Yes<br>12 weeks, social skills training	In the table below, treatment is social skills training, social group is a comparison social group intervention, and waitlist<br>**Waitlist**<br>Part 1: PRE = 18.5 (5.6) POST = 19.1 (4.8)<br>Part 2: PRE = 40.8 (10.4) POST = 39.5 (7.9)<br>Part 3: PRE = 43.4 (9.1) POST = 40.6 (7.2)<br>**Social group**<br>Part 1: PRE = 17.3 (4.1) POST = 15.6 (5.3)<br>Part 2: PRE = 42.4 (9.2) POST 41.7 (12.1)

			Part 3: PRE = 43.9 (9.4) POST = 43.6 (7.2)<br>**Social skills training**<br>Part 1: PRE = 20.2 (4.4) POST = 21.7 (3.3)<br>Part 2: PRE = 48.1 (9.9) POST = 45.1 (10.4)<br>Part 3: PRE = 47.3 (8.3) POST 49.2 (6.8)<br>.44 treatment effect size in the TBI population, .30 placebo effect size - non-significant treatment by time interaction
Bornhofen et al. (2008)a	12 TBI	Yes<br>-25 hours across 8 weeks of emotion perception training (EPT)<br>-Waitlist	PART I<br>Pretreatment: EPT: 20.07 (1.07) Waitlist: 20.89 (4.09)<br>Posttreatment: EPT: 27 (1.0) Waitlist: 21.83 (3.19)<br>PART II<br>Pretreatment: EPT: 46.47 (5.65) Waitlist: 45.11 (5)<br>Postreatment: EPT: 54.4 (2.3) Waitlist 47 (4.43)<br>PART III<br>Pretreatment: EPT: 46.4 (3.85) Waitlist 43 (2.97)<br>Posttreatment: EPT: 54.6 (2.51) Waitlist 41.83 (3.92)
Bornhofen et al. (2008)b	18 TBI	Yes<br>-10 weeks of errorless learning (EL)<br>-10 weeks of self-instructional training (SIL)	PART I<br>Pretreatment: SIL: 20.20 (3.5) Waitlist: 18.94 (4.09), EL: 16.67 (5.86)<br>Posttreatment: SIL: 21.40 (3.91) Waitlist: 19.60 (7.44), EL: 18.75 (5.12)<br>PART II: .47 Effect Size of SIT vs WL<br>Pretreatment: SIL: 40.75 (15.71), Waitlist: 42.89 (11.10), EL 40.75 (15.71)<br>Postreatment: SIL: 43.80 (13.03), Waitlist 35.80 (12.81), EL 40.75 (13.94)<br>PART III: ns treatment effect<br>Pretreatment: SIL: 41.87 (8.01) Waitlist 42.89 (11.10), EL 41.92 (11.27)<br>Posttreatment: SIL: 40.25 (13.43) Waitlist 40.20 (7.95) EL 40.25 (13.43)

４．妥当性

(Relationship to social cognitive measures relative to other abilities and constructs)

※この項目では、他の社会認知機能との関連が強い尺度が良いと考える。

Author and Date	Sample	Findings

McDonald et al. (2006)	70 adults with brain injuries	(Correlation coefficients and p values listed below)<br>**Part 1** .69 (.01) with Ekman Face Identification; .70 (.01) with Ekman Face Matching.<br>**Part 2** .50 (.01) with Ekman Face Identification; .45 (.01) with Ekman Face Matching; .49, .68 (.05) with second order ToM stories<br>**Part 3** .37 (.05) with Identification; .42 (.01) with Matching<br>**Part 1** .50 (.01) with premorbid IQ; .32 (.01), -.39 (.05) with Trails A; -.37 (.05) with Trails B; .25 (.05) with Digit Span; .27 (.05) with letter number sequencing; .33 (.01) with logical memory, Wechsler Faces I; .69 (.01), .35 (.05) with similarities, .66 (.01) with Matrix Reasoning, .45 (.05) with face recognition.<br>**Part 2** .45 (.01) with Symbol Search; -.53 (.01) with TrailsA; -.56 (.01) with TrailsB; .35 (.05) with Digit Span; .36 (.01) with Letter Number; .39 (.05) with Logical Memory; .50 (.01) with Faces 1; .31 (.05) with Verbal Paired Associates, .49 (.05) with similarities; 77 (.01) with matrix reasoning<br>**Part 3** .54 with Symbol search (.01); -.34 wih trailsA (.05); -.35 (.05) with trailsB; .30 (.01) digit span; .30 (.05) letter number sequence; .34 (.01) with logical memory, .42 with WAIS faces; .29 (.01) with similarities, .78 (.01) with matrix reasoning
Jahshan et al. (2007)	52 CS high in schizotypy<br>40 CS low in schizotypy	TASIT Part III correlated:<br>-.36 (.01) (short delay cured recall)<br>.32 (.05) (long delay free recall)<br>.48 (.01) (long delay cued recall)
McDonald et al. (2004)	21 TBI	Social manners, Use of Reinforcers, Egocentric Behaviour, and Partner Involvement were each correlated with Part 3 (r = .57, .57, .77, .49, respectively) (Rated using the Behavioral Referenced Rating System of Intermediate Social Skills)

(Correlations with Real-World Social Outcomes)

Author and Date	Sample	Findings
McDonald et al. (2006)	70 adults with brain injuries	**Part 1** *NS with all scales of interpersonal problem solving skills*<br>**Part 2** .49 (.05) with sending - performance scale of interpersonal problem solving, and .47 (.05) and sending-overall scales<br>**Part 3** .*NS with all Interpersonal Problem Solving Skills*
McDonald et al. (2004)	21 TBI	No correlation was found between the TASIT and the Social Performance Survey Schedule (modified)

Jahshan et al. (2007)	52 high in schizotypy<br>40 low in schizotypy	NS correlations between all three parts of the TASIT and academic functioning, social functioning, and family relationships as measured by the Social Adjustment Scale
Sparks et al. (2010)	25 HC<br>30 SCZ/SCZaff	**TASIT part 1 total:** Life-RIFT satisfaction (ß = −.43*)<br>**TASIT part 3 sarcasm:** LIFE-RIFT recreational engagement ((ß = −.53*)

文献

ORIGINAL CITATION:

Title: A new clinical tool for assessing social perception after traumatic brain injury

Author(s): McDonald, S; Flanagar, S; Rollins, J; et al.

Source: JOURNAL OF HEAD TRAUMA REHABILITATION Volume: 18 Issue: 3 Pages: 219-238 DOI: 10.1097/00001199-200305000-00001 Published: MAY-JUN 2003

Times Cited: 69 (from All Databases)

CITING ARTICLES

Title: The ecological validity of TASIT: A test of social perception

Author(s): McDonald, S; Flanagan, S; Martin, I; et al.

Source: NEUROPSYCHOLOGICAL REHABILITATION Volume: 14 Issue: 3 Pages: 285-302 DOI: 10.1080/09602010343000237 Published: JUL 2004

Times Cited: 25 (from All Databases)

Title: Social perception deficits after traumatic brain injury: Interaction between emotion recognition, mentalizing ability, and social communication

Author(s): McDonald, S; Flanagan, S

Source: NEUROPSYCHOLOGY Volume: 18 Issue: 3 Pages: 572-579 DOI: 10.1037/0894-4105.18.3.572 Published: JUL 2004

Times Cited: 59 (from All Databases)

Title: Reliability and validity of The Awareness of Social Inference Test (TASIT): A clinical test of social perception

Author(s): McDonald, Skye; Bornhofen, Cristina; Shum, David; et al.

Source: DISABILITY AND REHABILITATION Volume: 28 Issue: 24 Pages: 1529-1542 DOI: 10.1080/09638280600646185 Published: DEC 2006

Times Cited: 22 (from All Databases)

Title: Mismatch Negativity, Social Cognition, and Functioning in Schizophrenia Patients

Author(s): Wynn, Jonathan K.; Sugar, Catherine; Horan, William P.; et al.

Source: BIOLOGICAL PSYCHIATRY Volume: 67 Issue: 10 Pages: 940-947 DOI: 10.1016/j.biopsych.2009.11.024 Published: MAY 15 2010

Times Cited: 12 (from All Databases)

Title: Social cognition and interaction training (SCIT) for outpatients with schizophrenia: A preliminary study
Author(s): Roberts, David L.; Penn, David L.
Source: PSYCHIATRY RESEARCH Volume: 166 Issue: 2-3 Pages: 141-147 DOI: 10.1016/j.psychres.2008.02.007 Published: APR 30 2009
Times Cited: 23 (from All Databases)
Title: Social skills treatment for people with severe, chronic acquired brain injuries: A multicenter trial
Author(s): McDonald, Skye; Tate, Robyn; Togher, Leanne; et al.
Source: ARCHIVES OF PHYSICAL MEDICINE AND REHABILITATION Volume: 89 Issue: 9 Pages: 1648-1659 DOI: 10.1016/j.apmr.2008.02.029 Published: SEP 2008
Times Cited: 23 (from All Databases)
Title: Theory of mind, neurocognition, and functional status in schizotypy
Author(s): Jahshan, Carol S.; Sergi, Mark J.
Source: SCHIZOPHRENIA RESEARCH Volume: 89 Issue: 1-3 Pages: 278-286 DOI: 10.1016/j.schres.2006.09.004 Published: JAN 2007
Times Cited: 45 (from All Databases)
Title: Social cognition, empathy and functional outcome in schizophrenia
Author(s): Sparks, Amy; McDonald, Skye; Lino, Bianca; et al.
Source: SCHIZOPHRENIA RESEARCH Volume: 122 Issue: 1-3 Pages: 172-178 DOI: 10.1016/j.schres.2010.06.011 Published: SEP 2010
Times Cited: 12 (from All Databases)
Title: Treating deficits in emotion perception following traumatic brain injury
Author(s): Bornhofen, Cristina; McDonald, Skye
Source: NEUROPSYCHOLOGICAL REHABILITATION Volume: 18 Issue: 1 Pages: 22-44 DOI: 10.1080/09602010601061213 Published: JAN 2008
Times Cited: 17 (from All Databases)
Title: Comparing strategies for treating emotion perception deficits in traumatic brain injury
Author(s): Bornhofen, Cristina; McDonald, Skye
Source: JOURNAL OF HEAD TRAUMA REHABILITATION Volume: 23 Issue: 2 Pages: 103-115 DOI: 10.1097/01.HTR.0000314529.22777.43 Published: MAR-APR 2008
Times Cited: 12 (from All Databases)

7. Ambiguous Intentions and Hostility Questionnaire (AIHQ)

1. 実用可能性と忍容性

(Practicality for Administration and Tolerability for Patients)

Notes:

Notes: Administration time for full version is about 15-20 minutes; 5-8 minutes for the short version. No information was available on drop-out or incompletion rates. However, the task involves the participant

writing a brief reason for character behaviors in each vignette and the rating of likert scales.

２．信頼性

(Test-Retest, Internal Consistency, Inter-rater Reliability as applicable)

Author and Date	Sample	Findings
Combs et al. (2007)	322 CS	Across intentional, ambiguous, and accidental situations, the average ICCs were high for both the hostility bias (range .91 - 99) and aggression bias ratings (range .93 - .99). Internal consistency: intentional (α = .85), ambiguous (α =.86), and accidental situations (α =.84).
Mancuso et al. (2011)	85 SSI	ICC's for two blinded raters was > .85 (aggression and hostile biases); SHORT VERSION
Combs et al. (2009)	50 HC, 32 SSI with persecutory delusions, 28 SSI without persecutory delusions	Raters trained to ICCs > .80; Agreement on hostility and aggression scores ranged from .80-.86; internal consistency of blame score was .74 clin, .78 nonclin (SHORT VERSION)
Roberts et al. (2009)	31 SSI	ICC was 0.85.<br>Cronbach's alpha of the Likert-rated Blame scores was 0.92 (SHORT VERSION)
Roberts et al. (2010)	50 SSI	ICCs > .75 for hostility and aggression bias ratings (SHORT VERSION)
Waldheter et al. (2005)	29 SSI	ICCs for the hostility bias ranged from .87 to 1.00
Elnakeeb et al. (2010)	150 participants in Egypt SCZ	Internal consistency: Blame scores (intentional, accidental, and ambiguous situations) .81 or higher; hostility bias (.52-.63), aggression bias (.63-.70).<br>Test-retest reliability: Blame scores (.66-.87), hostile bias (.52-64), and aggression bias (.22-.70).
Ann et al. (2010)	39 HC, 24 ultra high risk participants, and 20 young first episode from South Korea	α: .85-.89<br>ICCS: Hostility bias (.85-.93); aggression bias (.71-.88) (SHORT VERSION)

３．有用性

(Floor and Ceiling Effects, Normality of Distributions)

Author and Date	Sample	Findings

Combs et al. (2009)	50 HC, 32 SSI with persecutory delusions, and 28 SSI without persecutory delusions.	HC: hostility score = 1.5 (SD=.31), blame score = 2.5 (SD=.61), aggression score = 1.4 (SD=.14) SSI with persecutory delusions: hostility bias = 2.5 (SD=.52), blame score = 3.1 (SD=.60), aggression score = 1.7 (SD=.47)<br>(Short version)
Horan et al. (2009)	31 SSI	Range of scores 1.5 (SD=.6) (hostility score) to 3.0 (SD=.8) (blame score)
Mancuso et al. (2011)	85 SSI	Range of scores were from 1.80 (SD=.53) (hostility score) to 2.97 (SD=.96) (blame score)
Roberts et al. (2009)	31 SSI	Range of scores were from 1.51 (SD=.60) (hostility score) to 2.93 (SD=.95) (blame score)
Combs et al. (2007)	322 CS	Range of scores: hostility accidental item mean = 1.1 (SD = .22), and Aggression accidental = 1.3 (SD = .33), to Blame intentional mean = 4.3 (SD = .55)

(Utility as a Repeated Measure)

※この項目では、自然経過では不変で介入によってより大きく動く尺度が良いと考える。

Author and Date	Sample	Treatment Study?<br>Testing interval	Findings
Roberts et al. (2009)	31 SSI	Yes<br>20 weeks	SCIT (social cognition and interaction training) + TAU versus TAU alone: no treatment effects and no changes resulting from TAU
Roberts et al. (2010)	50 SSI	Yes<br>5 months	No significant improvement from SCIT in an uncontrolled open trial.
Horan et al (2009)	31 SSI	Yes<br>6 weeks	No impact of social cognition training or TAU on AIHQ
Horan et al. (2011)	111 SSI; 67 HC	Yes<br>12 weeks	Social cognition training + neurocognitive remediation = reduction in blame scores and a trend level reduction in aggression biases.
Penn et al. (2005)	7 inpatients (SSI)	Yes<br>3 months	Open trial of social cognition training = trend level, moderate effect size reduction in hostile (.54) and aggression (.33) biases.
Lahera et al (in press).	37 outpatients with bipolar and	Yes<br>18 weeks	Social cognition training=Effect size (-.55) reduction in hostility bias<br>TAU = Effect size (.49) increase in hostility bias (opposite direction to expectation)

	schizoaffective disorder	

４．妥当性

(Relationship to social cognitive measures relative to other abilities and constructs)

※この項目では、他の社会認知機能との関連が強い尺度が良いと考える。

Author and Date	Sample	Findings
Mancuso et al. (2011)	85 SSI	-No significant correlations with emotion perception, ToM, and social perception<br>- AIHQ factor correlated .22 ($p < .05$) with MATRICs cognitive battery
Combs et al. (2009)	50 HC, 32 SSI with persecutory delusions, and 28 SSI without persecutory delusions.	AIHQ hostility bias correlated with IPSAQ personalizing bias (r=.35)<br>AIHQ aggression bias correlated with IPSAQ externalizing bias (r=.20)<br>(Full sample; both are statistically significant)

(Correlations with Real-World Social Outcomes)

Author and Date	Sample	Findings
Waldheter et al. (2005)	29 inpatients with SSI	AIHQ predicted severity of violence in an inpatient unit (beta = .20, $p < .05$)

文献

ORIGINAL CITATION:

Title: The Ambiguous Intentions Hostility Questionnaire (AIHQ): a new measure for evaluating hostile social-cognitive biases in paranoia.

Author(s): Combs, Dennis R; Penn, David L; Wicher, Melanie; et al.

Source: Cognitive neuropsychiatry Volume: 12 Issue: 2 Pages: 128-43 DOI: 10.1080/13546800600787854

Published: 2007-Mar

Times Cited: 11

CITING ARTICLES

Title: Social cognition in psychosis: Multidimensional structure, clinical correlates, and relationship with functional outcome

Author(s): Mancuso, Francesco; Horan, William P.; Kern, Robert S.; et al.

Source: SCHIZOPHRENIA RESEARCH Volume: 125 Issue: 2-3 Pages: 143-151 DOI: 10.1016/j.schres.2010.11.007 Published: FEB 2011

Times Cited: 7 (from All Databases)
Title: Perceptions of hostility by persons with and without persecutory delusions.
Author(s): Combs, Dennis R; Penn, David L; Michael, Christopher O; et al.
Source: Cognitive neuropsychiatry Volume: 14 Issue: 1 Pages: 30-52 Published: 2009-Jan
Times Cited: 5 (from All Databases)
Title: Social Cognition in Schizophrenia: An NIMH Workshop on Definitions, Assessment, and Research Opportunities
Author(s): Green, Michael F.; Penn, David L.; Bentall, Richard; et al.
Source: SCHIZOPHRENIA BULLETIN Volume: 34 Issue: 6 Pages: 1211-1220 DOI: 10.1093/schbul/sbm145 Published: NOV 2008
Times Cited: 50 (from All Databases)
Title: Social cognition and interaction training (SCIT) for outpatients with schizophrenia: A preliminary study
Author(s): Roberts, David L.; Penn, David L.
Source: PSYCHIATRY RESEARCH Volume: 166 Issue: 2-3 Pages: 141-147 DOI: 10.1016/j.psychres.2008.02.007 Published: APR 30 2009
Times Cited: 23 (from All Databases)
Title: Subtypes of paranoia in a nonclinical sample.
Author(s): Combs, Dennis R; Penn, David L; Chadwick, Paul; et al.
Source: Cognitive neuropsychiatry Volume: 12 Issue: 6 Pages: 537-53 DOI: 10.1080/13546800701707306 Published: 2007-Nov
Times Cited: 3 (from All Databases)
Title: Transportability and Feasibility of Social Cognition and Interaction Training (SCIT) in Community Settings
Author(s): Roberts, David L.; Penn, David L.; Labate, Daniella; et al.
Source: BEHAVIOURAL AND COGNITIVE PSYCHOTHERAPY Volume: 38 Issue: 1 Pages: 35-47 DOI: 10.1017/S1352465809990464 Published: JAN 2010
Times Cited: 6 (from All Databases)
Title: Efficacy and specificity of Social Cognitive Skills Training for outpatients with psychotic disorders
Author(s): Horan, William P.; Kern, Robert S.; Tripp, Cory; et al.
Source: JOURNAL OF PSYCHIATRIC RESEARCH Volume: 45 Issue: 8 Pages: 1113-1122 DOI: 10.1016/j.jpsychires.2011.01.015 Published: AUG 2011
Times Cited: 3 (from All Databases)
Title: Pilot study of social cognition and interaction training (SCIT) for schizophrenia
Author(s): Penn, D; Roberts, DL; Munt, ED; et al.
Source: SCHIZOPHRENIA RESEARCH Volume: 80 Issue: 2-3 Pages: 357-359 DOI: 10.1016/j.schres.2005.07.011 Published: DEC 15 2005
Times Cited: 32 (from All Databases)
Title: Utility of social cognition and insight in the prediction of inpatient violence among individuals with a

severe mental illness

Author(s): Waldheter, EJ; Jones, NT; Johnson, ER; et al.

Source: JOURNAL OF NERVOUS AND MENTAL DISEASE Volume: 193 Issue: 9 Pages: 609-618 DOI: 10.1097/01.nmd.0000177788.253573.de Published: SEP 2005

Times Cited: 10 (from All Databases)

Title: Attributional style of Egyptians with schizophrenia

Author(s): Elnakeeb, Mayar; Abdel-Dayem, Samia; Gaafar, Maha; et al.

Source: INTERNATIONAL JOURNAL OF MENTAL HEALTH NURSING Volume: 19 Issue: 6 Pages: 445-456 DOI: 10.1111/j.1447-0349.2010.00707.x Published: DEC 2010

Times Cited: 0 (from All Databases)

Title: Attribution bias in ultra-high risk for psychosis and first-episode schizophrenia

Author(s): An, Suk Kyoon; Kang, Jee In; Park, Jin Young; et al.

Source: SCHIZOPHRENIA RESEARCH Volume: 118 Issue: 1-3 Pages: 54-61 DOI: 10.1016/j.schres.2010.01.025 Published: MAY 2010

Times Cited: **10** (from All Databases)

Title: Social cognition and interaction training (SCIT) for outpatients with bipolar disorder.

Authors: Lahera, G., Benito, A., Montes, J. M., Fernandez-Liria, A., Olbert, C. M., & Penn, D. L.

Source: JOURNAL OF AFFECTIVE DISORDERS, in press.

Title: Social cognitive skills training in schizophrenia: An initial efficacy study of stabilized outpatients.

Authors: Horan W. et al.

Source: SCHIZOPHRENIA RESEARCH, Volume 107, Pages: 47-54

8. Social Attribution Task- Multiple Choice（SAT-MC）

1. 実用可能性と忍容性

(Practicality for Administration and Tolerability for Patients)

Author and Date	Sample	Notes

２. 信頼性

(Test-Retest, Internal Consistency, Inter-rater Reliability as applicable)

Author and Date	Sample	Findings
Lee et al., 2018	120 participants (30 per group; Korean SZ; Korean HC; North American SZ; North American HC)	Good internal consistency regardless of the clinical and cultural group as evidence by Cronbach's alpha >= 0.78 in all groups.
Johannesen et al., 2018	32 schizophrenia (SZ) and 30 substance use disorder (SUD) participants	・Test-retest: reliability of both forms (SAT-MC and SAT-MC-II) were above acceptable levels in SZ (r = 0.74–0.86) and higher than obtained for SCOPE comparison measures. Reliability estimates were lower in SUD (r = 0.49–0.57), however, mean scores were highly stable across sessions.<br>・Internal consistency: good inter-item consistency (alpha 0.83–0.89) with only marginal, statistically non-significant improvement by select item removal.

３. 有用性

(Floor and Ceiling Effects, Normality of Distributions)

Author and Date	Sample	Findings
Hasson-Ohayon et al., 2018	81 adults with a schizophrenia spectrum disorder in a non-acute phase of the disorder	Median score 12.00, mean score 11.65 (SD=4.12), minimum 2.00, maximum 19.00 out of 19.00.

Johannesen et al., 2018	32 schizophrenia (SZ) and 30 substance use disorder (SUD) participants	・Floor and ceiling effects: 25% and 22% of SZ participant performing at floor on first administration of SAT-MC and SAT-MC-II, respectively, and 3.10% performing at ceiling on 2nd administration of both forms. A maximum of 13.30% performed at floor and ceiling across forms and administrations in SUD.<br>・Mean score: SZ 10.16 (SD=5.01), SUD 13.70 (SD=4.63).<br>・Deficit distribution: Deficit was observed in approximately 60% of SZ, compared to 30% or less in SUD, for both the SAT-MC [SZ = 62.5%, SUD = 23.3%, $\chi 2(1) = 9.66$, $p < .01$] and SAT-MC-II [SZ = 59.4%, SUD = 30.0%, $\chi 2(1) = 5.40$, $p < .05$]. Agreement in deficit classification between the two forms was moderate in strength [κ = 0.575 (95% CI, .370 to 0.780), $p < .0001$].
Slane et al., 2014	48 children recruited from local public schools	Mean 12.96 (SD=3.63)
Lazar et al., 2014	57 Undergraduate students at a liberal arts university	Male Mean score 16.32 (SD=2.43), Female Mean score 15.70 (SD=2.41)
Bell et al., 2013	77 adult schizophrenia or schizoaffective	Mean (SD): Total 10.8 (4.5)
Ikezawa et al., 2012	28 schizophrenia or schizoaffective, 24 healthy controls	Scz mean score 12.1 (SD=4.2), HC mean score 15.5 (SD=3.2)

(Utility as a Repeated Measure)

※この項目では、自然経過では不変で介入によってより大きく動く尺度が良いと考える。

Author and Date	Sample	Treatment Study? Testing Interval	Findings
Johannesen et al., 2018	32 schizophrenia (SZ) and 30 substance use disorder (SUD) participants	2 weeks	There was little evidence of practice effect, either between forms taken within a single session, or over a two-week retest period; however, variability in performance between forms was observed in SUD.

４. 妥当性

(Relationship to social cognitive measures relative to other abilities and constructs)

※この項目では、他の社会認知機能との関連が強い尺度が良いと考える。

Author and Date	Sample	Findings
James et al. (2019)	72 adults with schizophrenia in a nonacute phase	Participants with intact SI (social inference; as measured with SAT-MC) had higher MCCB visual and verbal learning and SC scores. ER(as measured with BLERT) and SI are differentially related to cognitive processes.
Lee et al., 2018	120 participants (30 per group; Korean SZ; Korean HC; North American SZ; North American HC)	・Confirmatory factor analysis confirmed the one-factor model with a good model fit (chi(2) = 188.122, TLI = 0.958, CFI = 0.963, RMSEA = 0.045)<br>・HC had higher scores than SZ in SAT-MC regardless of culture (mean = 15.38, SD = 3.44 for HC; mean = 11.33 SD = 4.44 for SZ)<br>・Combined samples of SZs and HCs showed that the SAT-MC score was moderately correlated with the Hinting task(Korea: $r = 0.430$, $p < 0.01$; North America: $r = 0.432$, $p < 0.01$), Eyes test (Korea: $r = 0.465$, $p < 0.01$; North America: $r = 0.374$, $p < 0.01$) , emotion recognition tasks (K-FEIT: $r = 0.341$, $p < 0.01$; BLERT: $r = 0.526$, $p < 0.01$)), and estimated IQ (Korea: Information subtest of the K-WAIS-IV $r = 0.317$, $p < 0.01$; North America: the Vocabulary and Block Design subtest combination of WAIS-III $r = 0.491$, $p < 0.01$)<br>・Combined sample of SZs（KSZ and ASZ) were positively correlated with the Hinting task ($r = 0.325$, $p < 0.05$) but not with the Eyes test ($r = 0.207$, $p = 0.12$).<br>・Combined sample of HCs (KHC and AHC) were positively correlated with the Eyes test, but not with the Hinting task (Hinting task: $r = 0.117$, $p = 0.37$; Eyes test: $r = 0.331$, $p < 0.01$).<br>・Significantly correlated with emotion recognition tasks in HCs(K-FEIT: $r = 0.395$, $p = 0.03$; BLERT: $r = 0.645$, $p < 0.01$), but not in SZs (K-FEIT: $r = 0.137$, $p = 0.47$; BLERT: $r = 0.352$, $p = 0.06$).
Johannesen et al., 2018	32 schizophrenia (SZ) and 30 substance use disorder (SUD) participants	・Construct validation:<br>SZ: similar patterns of association with SCOPE tests, with medium correlations with BLERT (SAT-MC 0.51 ($p<.01$), SAT-MC-II 0.51 ($p<.01$)) and TASIT (SAT-MC 0.51 ($p<.01$), SAT-MC-II 0.46 ($p<.01$)) and no appreciable relationship to the Hinting Task (SAT-MC 0.04, SAT-

		MC-II 0.16) or AIHQ (SAT-MC -0.17～-0.01, SAT-MC-II -0.08～0.14). SUD: differed slightly from SZ; medium-to-large correlations with BLERT (SAT-MC 0.52 (p<.01), SAT-MC-II 0.76 (p<.01)), but non-significant correlations with TASIT (SAT-MC 0.36, SAT-MC-II 0.19), unique significant correlation between SAT-MC-II and AIHQ Aggression (0.44 (p<.05)).<br>・Relationship to interpersonal functions: Generally small and not statistically significant. The two SAT-MC forms were similar in strength of association across groups. However, the SAT-MC was uniquely related to VR-12 (-0.43 (p<.05)) in SUD in a direction suggesting better health status with higher SAT-MC performance.<br>・Partial correlations were used to repeat analyses while controlling for Picture Completion; results suggest that relationships between SAT-MC forms and other SC tests are robust to affects of visual attention in SZ.
Burger-Caplan et al., 2016	23 children with ASD, 57 age-matched and verbal IQ matched typically developing children	・SAT-MC scores were positively correlated with age (r=0.474) while being independent from verbal IQ (r=0.236).<br>・SAT-MC was strongly correlated with Vineland Adaptive Behavior Scales Communication (r=0.464) and Socialization (r=0.482) scores, but not with Daily Living Skills scores (r=0.116), suggesting that the implicit social cognitive ability underlying performance on the Social Attribution Task, Multiple Choice is associated with real-life social adaptive function.
Minor et al., 2013	46 schizophrenia, 22 schizoaffective	Significant inverse correlations with Disorganized factor of PANSS (-0.32 (p < 0.01)), but no correlations with Reality distortion (0.03) or Negative (0.04) factors.
Slane et al., 2014	48 children recruited from local public schools	Associated with multiple OXTR single nucleotide polymorphisms.
Lazar et al., 2014	57 Undergraduate students at a liberal arts university	Predicted left hemispheric cortical activity (N170 peak latency).
Bell et al., 2013	77 adult schizophrenia or schizoaffective	Significant correlation with SANS Anhedonia/Asociality (0.249 (p ＜.05)).

(Correlations with Real-World Social Outcomes)

Author and Date	Sample	Findings

文献

ORIGINAL CITATION

Title: Social Attribution Test- Multiple Choice (SAT-MC) in Schizophrenia: Comparison with Community Sample and Relationship to Neurocognitive, Social Cognitive and Symptom Measures.
Author(s): Bell, Morris D.; Fiszdon, Joanna M.; Greig, Tamasine C.; Wexler, Bruce E.
Source: SCHIZOPHRENIA RESEARCH Volume: 122 Issue: 1-3 Pages: 164-171 DOI: 10.1016/j.schres.2010.03.024 Published: 2010 Apr 18
Times Cited: 44 (from Web of Science)

CITING ARTICLES

Title: Neurocognitive and Metacognitive Profiles of Intact Social Cognition in Prolonged Schizophrenia
Author(s): James, Alison V.; Johannesen, Jason K.; Lysaker, Paul H.
Source: JOURNAL OF NERVOUS AND MENTAL DISEASE Volume: 206 Issue: 12 Pages: 907-912 DOI: 10.1097/NMD.0000000000000900 Published: DEC 2018
Times Cited: 1 (from Web of Science)

Title: The centrality of cognitive symptoms and metacognition within the interacting network of symptoms, neurocognition, social cognition and metacognition in schizophrenia
Author(s): Hasson-Ohayon, Ilanit; Goldzweig, Gil; Lavi-Rotenberg, Adi; et al.
Source: SCHIZOPHRENIA RESEARCH Volume: 202 Pages: 260-266 DOI: 10.1016/j.schres.2018.07.007 Published: DEC 2018
Times Cited: 8 (from Web of Science)

Title: Measuring theory of mind in schizophrenia research: Cross-cultural validation
Author(s): Lee, Hyeon-Seung; Corbera, Silvia; Poltorak, Ania; et al.
Source: SCHIZOPHRENIA RESEARCH Volume: 201 Pages: 187-195 DOI: 10.1016/j.schres.2018.06.022 Published: NOV 2018
Times Cited: 3 (from Web of Science)

Title: The Social Attribution Task - Multiple Choice (SAT-MC): Psychometric comparison with social cognitive measures for schizophrenia research

Author(s): Johannesen, Jason K.; Fiszdon, Joanna M.; Weinstein, Andrea; et al.
Source: PSYCHIATRY RESEARCH Volume: 262 Pages: 154-161 DOI: 10.1016/j.psychres.2018.02.011 Published: APR 2018
Times Cited: 4 (from Web of Science)

Title: Predicting social and communicative ability in school-age children with autism spectrum disorder: A pilot study of the Social Attribution Task, Multiple Choice
Author(s): Burger-Caplan, Rebecca; Saulnier, Celine; Jones, Warren; et al.
Source: AUTISM Volume: 20 Issue: 8 Pages: 952-96 DOI: 10.1177/1362361315617589 Published: NOV 2016
Times Cited: 1 (from Web of Science)

Title: Necessary, but not sufficient: Links between neurocognition, social cognition, and metacognition in schizophrenia are moderated by disorganized symptoms
Author(s): Minor, Kyle S.; Lysaker, Paul H.
Source: SCHIZOPHRENIA RESEARCH Volume: 159 Issue: 1 Pages: 198-204 DOI: 10.1016/j.schres.2014.08.005 Published: OCT 2014
Times Cited: 25 (from Web of Science)

Title: Social cognition, face processing, and oxytocin receptor single nucleotide polymorphisms in typically developing children
Author(s): Slane, Mylissa M.; Lusk, Laina G.; Boomer, K. B.; et al.
Source: DEVELOPMENTAL COGNITIVE NEUROSCIENCE Volume: 9 Pages: 160-171 DOI: 10.1016/j.dcn.2014.04.001 Published: JUL 2014
Times Cited: 14 (from Web of Science)

Title: Social cognition and neural substrates of face perception: Implications for neurodevelopmental and neuropsychiatric disorders
Author(s): Lazar, Steven M.; Evans, David W.; Myers, Scott M.; et al.
Source: BEHAVIOURAL BRAIN RESEARCH Volume: 63 Issue: 2 Pages: 1-8 DOI: 10.1016/j.bbr.2014.01.010 Published: APR 15 2014
Times Cited: 14 (from Web of Science)

Title: Social Cognitive Impairments and Negative Symptoms in Schizophrenia: Are There Subtypes with Distinct Functional Correlates?
Author(s): Bell, Morris D.; Corbera, Silvia; Johannesen, Jason K.; et al.
Source: SCHIZOPHRENIA BULLETIN Volume: 39 Issue: 1 Pages: 186-196 DOI: 10.1093/schbul/sbr125 Published: JAN 2013

Times Cited: 44 (from Web of Science)

Title: Empathy in electrodermal responsive and nonresponsive patients with schizophrenia
Author(s): Ikezawa, Satoru; Corbera, Silvia; Liu, Jiacheng; et al.
Source: SCHIZOPHRENIA RESEARCH Volume: 142 Issue: 1-3 Pages: 71-76 DOI: 10.1016/j.schres.2012.09.011 Published: DEC 2012
Times Cited: 11 (from Web of Science)

9. Adult Faux Pas

1. 実用可能性と忍容性

(Practicality for Administration and Tolerability for Patients)

Author and Date	Sample	Findings
Bora et al. (2006)	50 SCZ	36 items, multiple choice, requires no informant rating
Notes: The above is the standard stimulus set. It is typically administered on a PC or laptop, is quick to score, and takes about 25-30 minutes.		
Ahmed and Miller (2011)	123 HC	The Faux Pas test requires one to be able to clearly express their responses in a verbal format.
Gregory et al. (2002)	19 frontotemporal dementia<br>12 Alzheimer's disease<br>16 HC	A few (*n* = 3) of the fvFTD group were unable to complete all of the stories due to their limited tolerance of testing.
Hooker et al. (2011)	21 SCZ/SCZaff<br>17 HC	The experimenter read each scenario aloud. Participants referred to a printed copy, as necessary, to minimize memory demands.
MacPherson, Phillips, and Della Sala (2002)	Three age groups involving 30 healthy participants in each (15 men, 15 women); 20-38, 40-59, 61-80	One of the older participants was excluded from the analysis as he claimed that a faux pas had been committed in all 20 stories.

2. 信頼性

(Test-Retest, Internal Consistency, Inter-rater Reliability as applicable)

Author and Date	Sample	Findings
Gregory et al. (2002)	47 participants: 19 patients with frontotemporal dementia, 12 with Alzheimer's disease, and 16 HC	ICC (2 independent raters) = 0.98.
Zhu et al. (2007)	40 SCZ and 31 HC	Test-retest reliability (3 months): .83

		ICC = .76
Ahmed and Miller (2011)	123 HC	ICC > .89
Ferguson and Austin (2010)	162 CS	α =.95
Spek and Scholte (2010)	32 adults with HFA, 29 adults with Asperger syndrome and 32 HC	Concordance between two raters: 95%
Zalla et al. (2009)	15 adults with Aspergers or HFA<br>15 HC	ICC = 0.95.

３．有用性

(Floor and Ceiling Effects, Normality of Distributions)

Author and Date	Sample	Findings
Stone, Baron-Cohen, and Knight (1998)	5 Bilateral damage to orbitofrontal cortex<br>5 unilateral damage in left dorsolateral prefrontal cortex<br>5 HC	Patients (DF, OF) mean correct false belief problems with memory load: 66%, 100%<br>Patients (DF, OF) mean correct false belief problems with no memory load: 98%, 100%<br>Patients (DF, OF) mean correct 2nd order false belief problems with memory load: 82%,100%<br>Patients (DF, OF) mean correct 2nd order false belief problems with no memory load: 99%, 100%<br>HC: 100% on all tasks
Gregory et al. (2002)	19 frontotemporal dementia<br>12 alzheimers<br>16 HC	Frontotemporal = .67 (.3)<br>Alzheimers = .88 (.1)<br>HC = .95 (.1)
Abu-Akel and Abushua'leh (2004)	24 SCZ (divided into violent and non-violent groups)	Violent paranoid scz 54.2%; nonviolent paranoid scz 68.8%<br>(no SDs reported)
Martino et al. (2007)	21 SCZ<br>15 HC	ToM index<br>SCZ = 82% (11%)<br>HC = 94% (5%)<br>Memory index

		SCZ = 89% (9%)<br>HC = 91% (5%)
Herold et al. (2008)	18 SCZ<br>21 HC	SCZ = 6.3 (2.6)<br>HC = 8.0 (1.4)<br>NOTE: scored out of 10
Shur and Shamay-Tsoory (2008)	28 SCZ<br>35 HC	SCZ = -17.25 (9.89)<br>HC = -12.54 (8.28)<br>This scoring is based on a composite of number of errors (lower negative number signifying more errors)
Hooker et al. (2011)	21 SCZ<br>17 HC	SCZ = 70% (20%) correct<br>HC = 94% (9%) correct
Wang et al. (2008)	53 HC<br>33 non-psychotic depressed<br>23 psychotic depressed	HC = 19.51 (.78)<br>Non-psychotic depressed = 16.88 (1.54)<br>Psychotic depressed = 14.87 (1.32)
Konstantakopoulosa et al. (2011)	36 SCZ<br>36 Controls	Patients 55.9 (14.1); controls 61.7 (12.4)
MacPherson, Phillips, and Della Sala (2002)	30 HC (divided into young, middle-aged and older)	Young 8.6 (5.4); middle aged 9.7 (5.7); older 7.9 (4.0)
Milders, Fuchs, and Crawford 2003	17 HC<br>17 post-traumatic amnesia	HC = 34.1 (3.3)<br>Post-traumatic amnesia = 28 (7.5)
Ahmed and Miller (2011)	123 HC	93.3% (7%)
Spek and Scholte (2010)	32 adults with HFA<br>29 adults with Asperger syndrome<br>32 HC	HFA = 20.28 (3.40)<br>ASP = 18.97 (3.95)<br>HC = 22.22 (2.70)
Zalla et al. (2009)	15 HFA/AS<br>15 HC	AS/HFA 39.6 (9.9); HC 54 (5.8)

(Utility as a Repeated Measure)

※この項目では、自然経過では不変で介入によってより大きく動く尺度が良いと考える。

See test-retest reliability section

４．妥当性

(Relationship to social cognitive measures relative to other abilities and constructs)

※この項目では、他の社会認知機能との関連が強い尺度が良いと考える。

Author and Date	Sample	Findings
Shur and Shamay-Tsoory (2008)	26 SCZ<br>35 HC	SCZ: 22% of the variance in Faux Pas was accounted for by a cognitive shifting test)
Hooker et al. (2011)	21 SCZ/SCZaff<br>17 HC	Global cognition (MATRICS) associated with Faux Pas (r=.57*).
Wang et al. (2008)	53 HC<br>33 non-psychotic depressed<br>23 psychotic depressed	When controlling for BDI-II scores in patients only: Verbal fluency (r=.57*); IQ (r=10, ns); digit span (r=.12, ns).
Ahmed and Miller (2011)	123 HC	Eyes task (r=.13, ns); strange stories (r=.11, ns) Delis-Kaplin Executive Function System (D-KEFS) variables (all p's < .05), verbal fluency (r=.22*), problem solving (r=.22*), categorical processing (r=.19*).
Arguedas, Langdon, and Stevenson (2012)	21 SCZ/SCZaff	No correlation with IQ
Ferguson and Austin (2010)	162 HC	Eyes task (r=.28, p<.01), situational test of emotional understanding (r=.31, p<.01), situational test of emotion management (r=.21, p<.01), Trait Emotional Intelligence Questionnaire (r=.21, p<.01).
Spek and Scholte (2010)	32 HFA<br>29 Asperger syndrome<br>32 HC	Strange stories (r=.36*); Eyes test (r=-.18, ns).

(Correlations with Real-World Social Outcomes)

Author and Date	Sample	Findings
Zhu et al. (2007)	71 people (40 schizophrenia patients and 31	Faux pas 'recognition' and 'related' questions were significantly correlated with SFS subscales Independence (performance) (r=.349, p<.05) and

	matched normal controls)	Employment (r=.365, p<.05)
Milders, Fuchs, and Crawford 2003	17 patients (7 female) with posttraumatic amnesia; 17 healthy participants (7 female)	Neuropsychology Behavior and Affect Profile (NBAP) is a measure designed to assess the emotional and behavioral consequences of acquired brain damage. NBAP total score was correlated (r=-.61) with faux pas score, suggesting poor FP performance is related to more behavioral problems.

文献

ORIGINAL CITATION:

Title: Frontal lobe contributions to theory of mind
Author(s): Stone, VE; Baron-Cohen, S; Knight, RT
Source: JOURNAL OF COGNITIVE NEUROSCIENCE Volume: 10 Issue: 5 Pages: 640-656 DOI: 10.1162/089892998562942 Published: SEP 1998
Times Cited: 437 (from All Databases)

CITING ARTICLES

Title: Theory of mind in patients with frontal variant frontotemporal dementia and Alzheimer's disease: theoretical and practical implications
Author(s): Gregory, C; Lough, S; Stone, V; et al.
Source: BRAIN Volume: 125 Pages: 752-764 DOI: 10.1093/brain/awf079 Part: Part 4 Published: APR 2002
Times Cited: 232 (from All Databases)

Title: Impairments of social cues recognition and social functioning in Chinese people with schizophrenia
Author(s): Zhu, Chun-Yan; Lee, Tatia M. C.; Li, Xiao-Si; et al.
Source: PSYCHIATRY AND CLINICAL NEUROSCIENCES Volume: 61 Issue: 2 Pages: 149-158 DOI: 10.1111/j.1440-1819.2007.01630.x Published: APR 2007
Times Cited: 10 (from All Databases)

Title: 'Theory of mind' in violent and nonviolent patients with paranoid schizophrenia
Author(s): Abu-Akel, A; Abushua'leh, K
Source: SCHIZOPHRENIA RESEARCH Volume: 69 Issue: 1 Pages: 45-53 DOI: 10.1016/S0920-9964(03)00049-5 Published: JUL 1 2004
Times Cited: 28 (from All Databases)

Title: Neuropsychological frontal impairments and negative symptoms in schizophrenia
Author(s): Martino, Diego J.; Bucay, Demian; Butman, Judith T.; et al.
Source: PSYCHIATRY RESEARCH Volume: 152 Issue: 2-3 Pages: 121-128 DOI: 10.1016/j.psychres.2006.03.002 Published: AUG 30 2007
Times Cited: 20 (from All Databases)

Title: Regional gray matter reduction and theory of mind deficit in the early phase of schizophrenia: a voxel-based morphometric study
Author(s): Herold, R.; Feldmann, A.; Simon, M.; et al.
Source: ACTA PSYCHIATRICA SCANDINAVICA Volume: 119 Issue: 3 Pages: 199-208 DOI: 10.1111/j.1600-0447.2008.01297.x Published: MAR 2009

Times Cited: 15 (from All Databases)
Title: Integration of emotional and cognitive aspects of theory of mind in schizophrenia and its relation to prefrontal neurocognitive performance.
Author(s): Shur, Syvan; Shamay-Tsoory, Simone G; Levkovitz, Yechiel
Source: Cognitive neuropsychiatry Volume: 13 Issue: 6 Pages: 472-90 DOI: 10.1080/13546800802490034 Published: 2008-Nov
Times Cited: 13 (from All Databases)
Title: Theory of Mind Skills Are Related to Gray Matter Volume in the Ventromedial Prefrontal Cortex in Schizophrenia
Author(s): Hooker, Christine I.; Bruce, Lori; Lincoln, Sarah Hope; et al.
Source: BIOLOGICAL PSYCHIATRY Volume: 70 Issue: 12 Pages: 1169-1178 DOI: 10.1016/j.biopsych.2011.07.027 Published: DEC 15 2011
Times Cited: 2 (from All Databases)
Title: Theory of mind disability in major depression with or without psychotic symptoms: A componential view
Author(s): Wang, Yong-guang; Wang, Yi-qiang; Chen, Shu-lin; et al.
Source: PSYCHIATRY RESEARCH Volume: 161 Issue: 2 Pages: 153-161 DOI: 10.1016/j.psychres.2007.07.018 Published: NOV 30 2008
Times Cited: 22 (from All Databases)
Title: Apathy, cognitive deficits and functional impairment in schizophrenia
Author(s): Konstantakopoulos, George; Ploumpidis, Dimitris; Oulis, Panagiotis; et al.
Source: SCHIZOPHRENIA RESEARCH Volume: 133 Issue: 1-3 Pages: 193-198 DOI: 10.1016/j.schres.2011.07.003 Published: DEC 2011
Times Cited: 0 (from All Databases)
Title: Age, executive function, and social decision making: A dorsolateral prefrontal theory of cognitive aging
Author(s): MacPherson, SE; Phillips, LH; Della Sala, S
Source: PSYCHOLOGY AND AGING Volume: 17 Issue: 4 Pages: 598-609 DOI: 10.1037//0882-7974.17.4.598 Published: DEC 2002
Times Cited: 159 (from All Databases)
Title: Neuropsychological impairments and changes in emotional and social behaviour following severe traumatic brain injury
Author(s): Milders, M; Fuchs, S; Crawford, JR

Source: JOURNAL OF CLINICAL AND EXPERIMENTAL NEUROPSYCHOLOGY Volume: 25 Issue: 2 Pages: 157-172 DOI: 10.1076/jcen.25.2.157.13642 Published: APR 2003
Times Cited: 69 (from All Databases)
Title: Executive Function Mechanisms of Theory of Mind
Author(s): Ahmed, Fayeza S.; Miller, L. Stephen
Source: JOURNAL OF AUTISM AND DEVELOPMENTAL DISORDERS Volume: 41 Issue: 5 Pages: 667-678 DOI: 10.1007/s10803-010-1087-7 Published: MAY 2011
Times Cited: 2 (from All Databases)

- 151 -

Title: Neuropsychological Characteristics Associated with Olfactory Hallucinations in Schizophrenia
Author: Deborah Arguedas, Robyn Langdon, Richard Stevenson
Source: Journal of the International Neuropsychological Society pp 1-10. Year: 2012
Title: Associations of trait and ability emotional intelligence with performance on Theory of Mind tasks in an adult sample
Author(s): Ferguson, Fiona J.; Austin, Elizabeth J.
Source: PERSONALITY AND INDIVIDUAL DIFFERENCES Volume: 49 Issue: 5 Pages: 414-418 DOI: 10.1016/j.paid.2010.04.009 Published: OCT 2010
Times Cited: 5 (from All Databases)
Title: Theory of Mind in Adults with HFA and Asperger Syndrome
Author(s): Spek, Annelies A.; Scholte, Evert M.; Van Berckelaer-Onnes, Ina A.
Source: JOURNAL OF AUTISM AND DEVELOPMENTAL DISORDERS Volume: 40 Issue: 3 Pages: 280-289 DOI: 10.1007/s10803-009-0860-y Published: MAR 2010
Times Cited: 3 (from All Databases)
Title: Faux Pas Detection and Intentional Action in Asperger Syndrome. A Replication on a French Sample
Author(s): Zalla, Tiziana; Sav, Anca-Maria; Stopin, Astrid; et al.
Source: JOURNAL OF AUTISM AND DEVELOPMENTAL DISORDERS Volume: 39 Issue: 2 Pages: 373-382 DOI: 10.1007/s10803-008-0634-y Published: FEB 2009
Times Cited: 10 (from All Databases)

10. Face Emotion Discrimination Test (FEDT)

1. 実用可能性と忍容性

(Practicality for Administration and Tolerability for Patients)

Author and Date	Sample	Notes
Mueser et al. (1996)	28 inpatients (20 SCZ, 8 SCZaff); 15 controls	The task lasts approximately 15 minutes.

Notes: The FEDT consists of 30 pairs of photos presented via video or computer presentation (e.g. Powerpoint) for a maximum of 15 seconds each.

2. 信頼性

(Test-Retest, Internal Consistency, Inter-rater Reliability as applicable)

Author and Date	Sample	Findings
Mueser et al. (1996)	28 inpatients (20 SCZ, 8 SCZaff); 15 controls	α =.74 for patients and α =.65 for HC
Kerr and Neale (1993)	29 SCZ; 23 HC	α=.74 for patients and α=.70 for HC
Salem, Kring, and Kerr (1996)	23 male SCZ; 22 male HC	α =.41 for patients and α=.70 for HC
Penn et al. (2000)	39 SCZ in extended-care program; 35 SCZ in acute-care unit; 40HC	α = .37 for HC; α = .52 for acute-care SCZ; α = .41 for extended-care SCZ
Ihnen et al. (1998)	26 outpatient SCZ	α =0.70
Pinkham and Penn (2006)	49 patients (35 SCZ, 12 SCZaff, 2 Psychosis NOS); 44 HC	α =0.68
Matthews and Barch (2010)	40 outpatients (30 SCZ, 10 SCZaff); 40 HC	α =.73 for combined items in the face and voice tasks across the entire sample
Penn and Combs (2000)	40 inpatients (23 men, 17 women; 29 SCZ, 11 SCZaff)	The average internal consistency across three test administrations (described below) was 0.64.
Pinkham et al.	19 individuals __at	α=.68

(2007)	risk‘‘ for psychosis, 21 SSI early in illness (≤ 5 years), 28 chronic SSI; 21 healthy controls	
Bellack, Blanchard, and Mueser (1996)	35 inpatients with SCZ or SCZaff; 11 with bipolar disorder; 19 matched HC	α=.64

３．有用性

(Floor and Ceiling Effects, Normality of Distributions)

Author and Date	Sample	Findings
Mueser et al. (1996)	28 inpatients (20 SCZ, 8 SCZaff); 15 controls.	Mean % correct was 69.5% (SD=15.3%) for patients, 81.8% (SD=9.7%) for controls.
Kerr and Neale (1993)	29 SCZ; 23 HC	Mean item difficulty of .85 (variance .01), 67.4% (SD=15.8) correct for patients, 86.9% (SD=10.3%) correct for HC
Salem, Kring, and Kerr (1996)	23 male SCZ; 22 male HC	Mean correct was 23.22 (SD=3.78) for SCZ and 25.23 (SD=3.10) for HC.<br>Percent correct: 77.4% for SCZ (SD=12.6%), 84.1% (SD=10.3%) for HC.
Penn et al. (2000)	39 SCZ in extended-care program; 35 SCZ in acute-care unit; 40 HC.	Mean correct: controls 25.75 (SD=2.86), extended care 23.44 (SD=3.54), and acute care 21.29 (SD=4.07)<br>Percent correct: controls 85.8%, extended care 78.1%, acute care 71%
Ihnen et al. (1998)	26 outpatient SCZ	Mean correct was 24.1 (SD=3.6), percent correct was 80.3%
Pinkham and Penn (2006)	49 patients (35 SCZ, 12 SCZaff, 2 Psychosis NOS); 44 HC	Mean correct: HC 26.47 (SD=2.30) and patients 24.59 (SD=3.470)<br>Percent correct: HC 88.2%, patients 82%
Matthews and Barch (2010)	40 outpatients (30 SCZ, 10 SCZaff); 40 HC	Mean correct: patients 25.03 (SD=2.43) and HC 25.85 (SD=2.69)<br>Percent correct: patients 83.4%, controls 86.2%
Penn and Combs (2000)	40 inpatients (23 men, 17 women; 29	Number correct ranged across administrations from 22.5 (SD=3.7) to 27.3 (SD=1).

	SCZ, 11 SCZaff)	
Pinkham et al. (2007)	19 individuals __at risk'' for psychosis, 21 SSI early in illness (≤ 5 years), 28 chronic SSI; 21 HC	Mean correct: controls 26.67 (SD=2.22), "at-risk" 26.79 (SD=1.99), early SSI 24.86 (SD=3.29), chronic SSI 24.39 (SD=3.65).<br>Percent correct: controls 88.9%, ―at-risk‖ 89.3%, early SSI 82.9%, chronic SSI 81.3%
Addington, Saeedi, and Addington (2006)	50 first-episode psychosis; 53 multi-episode SCZ; 55 HC	Number correct ranged across patient groups from 24.6 (SD=3.1) to 26.6 (SD=2.0) out of 30.
Bellack, Blanchard, and Mueser (1996)	35 inpatients with SCZ or SCZaff; 11 with bipolar disorder; 19 matched HC	Mean correct: SCZ 23.46 (SD=3.35), Bipolar disorder 23.36 (SD=3.01), HC 24.79 (SD=3.12).<br>Percent correct: SCZ 78.2%, Bipolar 77.9%, HC 82.6%
Combs et al. (2007)	18 SCZ completing a social cognition treatment, 10 SCZ completing coping skills treatment	Number correct ranged across patient groups from 20.7 (SD=3.7) to 26 (SD=1.9) out of 30.
Penn et al. (2009)	873 SCZ who completed the FEDT immediately prior to randomization and 2 months post-baseline.	The FEDT scores had a ceiling effect (24.58 correct out of 30) (SD=3.40) and negative skew. However, the change score (FEDT 2-month scores minus FEDT baseline scores), had an approximately normal distribution, with the scores being symmetrical.
Leitman et al. (2005)	43 SCZ/SCZaff; 34 HC	Percent correct: SCZ 78.5% (SD=11.1%), HC 92% (4%).
Tas et al. (2012)	52 outpatient SCZ (19 completing a social cognition treatment and 26 completing social stimulation)	Pre/post-test groups ranged in number correct: 23.69 (SD=3.21) to 26.79 (SD=2.25) out of 30.
Addington et al. (2008)	86 clinical high-risk individuals; 50 first-	Mean correct: high-risk 25.76 (SD=1.85); first-episode 24.79 (SD=2.66); multi-episode 24.85 (SD=2.70); controls 26.64

	episode psychosis; 53 multi-episode SCZ; 55 non-psychiatric controls.	(SD=2.02)<br>Percent correct: high-risk 85.69%, first-episode 82.6%, multi-episode 82.8%, controls 88.8%
Silver and Shlomo (2001)	36 inpatient SCZ	Mean correct of 23.06 (SD=2.62), 76.9 % correct
Horan and Blanchard (2003)	45 SCZ (15 deficit, 30 nondeficit); 41 HC	Mean correct: deficit 24.43 (SD=3.32); nondeficit 24.63 (SD=3.35); HC 26.18 (SD=2.55)<br>Percent correct: deficit 81.4%, nondeficit 82.1%, HC 87.3%
Vaskinn et al. (2007)	31 SCZ; 21 bipolar I disorder; 31 HC	Mean correct: SCZ 25.7 (SD=2.2), bipolar disorder 26.5 (SD=2.0), HC26.4 (SD=2.3)<br>Percent correct: SCZ 85.7%, bipolar disorder 88.3%, HC 88%
Mathews and Barch (2010)	40 outpatients (30 SCZ, 10 SCZaff); 40 HC	Mean correct: SCZ 25.03 (SD=2.43), HC 25.85 (SD=2.69)<br>Percent correct: SCZ 83.4%, HC 86.2%
Pinkham et al. (2005)	23 SCZ; 21 HC	Mean correct: SCZ 24.25 (SD=4.63) and HC 27.05 (SD=1.47)<br>Percent correct: SCZ 80.8%, HC 90.2%
Erol et al. (2010)	57 SCZ; 58 healthy siblings; 58 HC	Mean correct: SCZ 23.4 (SD=3.3), siblings 24.9 (SD=3.1), HC 26.1 (SD=1.7).<br>Percent correct: SCZ 78%, siblings 83%, HC 87%

(Utility as a Repeated Measure)

※この項目では、自然経過では不変で介入によってより大きく動く尺度が良いと考える。

Author and Date	Sample	Treatment Study?<br>Testing Interval	Findings
Penn and Combs (2000)	10 inpatients (8 SCZ, 2 SCZaff) - a subset of 40 SCZ in the study	No<br>1 week	Mean number correct at baseline was 22.5 (SD=3.7), 22.9 at same-day post-test (SD=3.6), and 24.5 (SD=3.4) at one-week follow-up. No statistical comparisons among means were available.
Addington, Saeedi, and Addington (2006)	50 first-episode psychosis; 53 multi-episode SCZ; 55 HC	No<br>1 year	Time 1 means: first-episode 24.6 (SD=3.1), multi-episode 24.8 (SD=2.7), HC 25.9 (SD=2.6)<br>Time 2 means: first-episode 24.9 (SD=2.7), multi-episode 25.0 (SD=2.7), HC 26.6 (SD=2.0).

			Changes in the patient groups did not reach statistical significance.
Combs et al. (2007)	18 SCZ completing a social cognition treatment (SCIT), 10 SCZ completing coping skills treatment	Yes<br>18 weeks	Pre-test means: SCIT 22.6 (SD=2.3), coping skills 22.3 (SD=2.7)<br>Post-test means: SCIT 26.0 (SD=1.9), coping skills 20.7 (SD=3.7)
Tas et al. (2012)	52 outpatient SCZ (19 completing a social cognition treatment (F-SCIT) and 26 completing social stimulation (SS)	Yes<br>16 weeks	Pre-test means: F-SCIT 24.89 (SD=2.23), SS 24.04 (SD=3.18)<br>Post-test means: F-SCIT 26.79 (SD=2.25), SS 23.69 (SD=3.21)
Piskulic and Addington (2011)	103 SCZ (50 first-episode, 53 chronic)<br>- tested 1 year apart	No<br>1 year	No significant changes over time for facial affect recognition (FEIT and FEDT) ($t(102) = -1.50$)<br>- no raw data provided

４．妥当性

(Relationship to social cognitive measures relative to other abilities and constructs)

※この項目では、他の社会認知機能との関連が強い尺度が良いと考える。

Author and Date	Sample	Findings
Penn et al. (2000)	39 SCZ in extended-care program; 35 SCZ in acute-care unit; 40 HC.	FEDT was significantly correlated to FEIT only for the acutely ill participants (r = .39 p < .05), although this association was in the expected direction for the extended-care sample (r = .26).<br>The Benton test of facial recognition was significantly related to the FEDT (r = .33, p < .05) in the extended care sample.

Ihnen et al. (1998)	26 outpatient SCZ	FEDT was significantly correlated with FEIT (r=.35, P<0.05) and the social cue recognition test (r=.60, p<.01).<br>No significant correlations with age, gender, years of education, prior hospitalizations, neuroleptic dosage level, or symptomatology
Pinkham and Penn (2006)	49 patients (35 SCZ, 12 SCZaff, 2 Psychosis NOS); 44 HC<br>Note: SCST = Schema Component Sequencing Task	In HC:<br>Social cognitive tasks: r=.48 with BLERT, r=.53 with FEIT, *ns* with SCST, Hinting, or ToM vignettes<br>Neurocognitive tasks: r=.35 with WRAT, r=-.44 with Trails B, *ns* with immediate memory and Trails A.<br>In patients:<br>Social cognitive tasks: r=.33 with BLERT, r=.66 with FEIT, r=.39 with SCST # correct, r=.33 with ToM vignettes, *ns* with SCST time and Hinting<br>Neurocognitive tasks: r=.34 with WRAT, r=.40 with Immediate Memory, *ns* with Trails A and B
Silver and Shlomo (2001)	36 inpatient SCZ	A moderate correlation was found between FEIT and FEDT scores (r=0.42). Exclusion of outliers from the analysis reduced the correlation to non-significance (r=−0.2, P=0.3).<br>No significant correlations Benton facial recognition, mini mental test.
Addington et al. (2010)	55 clinical high-risk individuals; 43 first-episode psychosis; 53 chronic SCZ; 55 HC	Composite measure of FEIT/FEDT was significantly associated with the Social Cue Recognition Test (r=.59, p<.001) and neurocognition (r=.64, p<.001)
Mathews and Barch (2010)	40 outpatients (30 SCZ, 10 SCZaff); 40 HC	FEDT correlated with the voice emotion discrimination task for SCZ (r= .46, p<.01) and HC (r=.48, p<.01).<br>The TASIT did not significantly correlate with the FEDT (r= .15, p > .18) in SCZ.

(Correlations with Real-World Social Outcomes)

Author and Date	Sample	Findings
Mueser et al. (1996)	28 inpatients (20 SCZ, 8 SCZaff); 15 controls	FEDT was not significantly associated with the Conversation Probe Role Play (measure of social skill). FEDT was significantly correlated with the following subscales of the Social Behavior Schedule: social mixing (r=-.35, p<.05), altered activity level (r=-.34, p<.05), and personal

		appearance/hygiene (r=-.38, p<.05).
Ihnen et al. (1998)	26 outpatient SCZ	FEDT was not significantly correlated with a measure of overall social skill (OSS, r=.17, ns), but was correlated with the "gaze" subscale of the conversational probe role play (r=-.39, p<.05).
Pinkham and Penn (2006)	49 patients (35 SCZ, 12 SCZaff, 2 Psychosis NOS); 44 HC	For SCZ, better performance on the FEDT was not significantly associated with greater interpersonal skill.
Matthews and Barch (2010)	40 outpatients (30 SCZ, 10 SCZaff); 40 HC	FEDT was not significantly associated with better functional outcome (total score on the SFS/SAS measure).
Addington, Saeedi, and Addington (2006)	50 first-episode psychosis; 53 multi-episode SCZ; 55 HC	Facial affect (FEDT and FEIT) were significantly correlated with measures of social functioning (Quality of Life Scale) in the following groups: patients at baseline (r=.22, p<.05), patients at 1 yr follow-up (r=.42, p<.0001), HC at baseline (r=.48, p<.0001), but not HC at follow-up (r=.11, ns).
Penn et al. (2009)	873 SCZ who completed the FEDT immediately prior to randomization and 2 months post-baseline.	r=.10 (p<.01) for baseline QOL interpersonal relations
Addington et al. (2010)	55 clinical high-risk individuals; 43 first-episode psychosis; 53 chronic SCZ; 55 HC	Composite measure of FEIT/FEDT was significantly associated (p<.001) with: assessment of interpersonal problem solving (AIPPS) (r=.47), social functioning scale (SFS) (r=.36), and quality of life scale (QLS) (r=.54)
Matthews and Barch (2010)	40 outpatients (30 SCZ, 10 SCZaff); 40 HC	Correlations between Face/voice (VEIT/FEDT) and functional outcome are not significant for SCZ (r=-.022) or HC (r=-.007)

文献

ORIGINAL CITATION:

Title: FACIAL EMOTION DISCRIMINATION .2. BEHAVIORAL FINDINGS IN DEPRESSION

Author(s): GUR, RC; ERWIN, RJ; GUR, RE; et al.

Source: PSYCHIATRY RESEARCH Volume: 42 Issue: 3 Pages: 241-251 DOI: 10.1016/0165-1781(92)90116-K Published: JUN 1992
Times Cited: 228 (from All Databases)

CITING ARTICLES

Title: Emotion recognition and social competence in chronic schizophrenia
Author(s): Mueser, KT; Doonan, R; Penn, DL; et al.
Conference: 27th Annual Convention of the Association-for-Advancement-of-Behavior-Therapy Location: ATLANTA, GA Date: NOV, 1993
Sponsor(s): Assoc Adv Behav Therapy
Source: JOURNAL OF ABNORMAL PSYCHOLOGY Volume: 105 Issue: 2 Pages: 271-275 DOI: 10.1037/0021-843X.105.2.271 Published: MAY 1996
Times Cited: 198 (from All Databases)

Title: EMOTION PERCEPTION IN SCHIZOPHRENIA - SPECIFIC DEFICIT OR FURTHER EVIDENCE OF GENERALIZED POOR PERFORMANCE
Author(s): KERR, SL; NEALE, JM
Source: JOURNAL OF ABNORMAL PSYCHOLOGY Volume: 102 Issue: 2 Pages: 312-318 DOI: 10.1037/0021-843X.102.2.312 Published: MAY 1993
Times Cited: 225 (from All Databases)

Title: More evidence for generalized poor performance in facial emotion perception in schizophrenia
Author(s): Salem, JE; Kring, AM; Kerr, SL
Conference: 9th Annual Meeting of the Society-for-Research-in-Psychopathology Location: CORAL GABLES, FL Date: OCT, 1994
Sponsor(s): Soc Res Psychopathol
Source: JOURNAL OF ABNORMAL PSYCHOLOGY Volume: 105 Issue: 3 Pages: 480-483 DOI: 10.1037//0021-843X.105.3.480 Published: AUG 1996
Times Cited: 129 (from All Databases)

Title: Emotion recognition in schizophrenia: Further investigation of generalized versus specific deficit models
Author(s): Penn, DL; Combs, DR; Ritchie, M; et al.
Source: JOURNAL OF ABNORMAL PSYCHOLOGY Volume: 109 Issue: 3 Pages: 512-516 DOI: 10.1037/0021-843X.109.3.512 Published: AUG 2000
Times Cited: 75 (from All Databases)

Title: Social perception and social skill in schizophrenia
Author(s): Ihnen, GH; Penn, DL; Corrigan, PW; et al.
Source: PSYCHIATRY RESEARCH Volume: 80 Issue: 3 Pages: 275-286 DOI: 10.1016/S0165-1781(98)00079-1 Published: SEP 21 1998
Times Cited: 53 (from All Databases)

Title: Neurocognitive and social cognitive predictors of interpersonal skill in schizophrenia

Author(s): Pinkham, Amy E.; Penn, David L.
Source: PSYCHIATRY RESEARCH Volume: 143 Issue: 2-3 Pages: 167-178 DOI: 10.1016/j.psychres.2005.09.005 Published: AUG 30 2006
Times Cited: 70 (from All Databases)
Title: Emotion Responsivity, Social Cognition, and Functional Outcome in Schizophrenia
Author(s): Mathews, Jennifer R.; Barch, Deanna M.
Source: JOURNAL OF ABNORMAL PSYCHOLOGY Volume: 119 Issue: 1 Pages: 50-59 DOI: 10.1037/a0017861 Published: FEB 2010
Times Cited: 11 (from All Databases)
Title: Modification of affect perception deficits in schizophrenia
Author(s): Penn, DL; Combs, D
Source: SCHIZOPHRENIA RESEARCH Volume: 46 Issue: 2-3 Pages: 217-229 DOI: 10.1016/S0920-9964(00)00005-0 Published: DEC 15 2000
Times Cited: 49 (from All Databases)
Title: Emotion perception and social skill over the course of psychosis: a comparison of individuals "at-risk" for psychosis and individuals with early and chronic schizophrenia spectrum illness.
Author(s): Pinkham, Amy E; Penn, David L; Perkins, Diana O; et al.
Source: Cognitive neuropsychiatry Volume: 12 Issue: 3 Pages: 198-212 DOI: 10.1080/13546800600985557 Published: 2007-May
Times Cited: 36 (from All Databases)
Jean Addington, Huma Saeedi, Donald Addington, Facial affect recognition: A mediator between cognitive and social functioning in psychosis?, Schizophrenia Research, Volume 85, Issues 1–3, July 2006, Pages 142-150, ISSN 0920-9964, 10.1016/j.schres.2006.03.028.
Title: Cue availability and affect perception in schizophrenia
Author(s): Bellack, AS; Blanchard, JJ; Mueser, KT
Conference: International Congress for Schizophrenia Research Location: COLORADO SPRINGS, CO Date: APR 17-21, 1993
Source: SCHIZOPHRENIA BULLETIN Volume: 22 Issue: 3 Pages: 535-544 Published: 1996
Times Cited: 67 (from All Databases)
Title: Social Cognition and Interaction Training (SLIT) for inpatients with schizophrenia spectrum disorders: Preliminary findings
Author(s): Combs, Dennis R.; Adams, Scott D.; Penn, David L.; et al.
Source: SCHIZOPHRENIA RESEARCH Volume: 91 Issue: 1-3 Pages: 112-116 DOI: 10.1016/j.schres.2006.12.010 Published: MAR 2007
Times Cited: 61 (from All Databases)
Title: The effects of antipsychotic medications on emotion perception in patients with chronic schizophrenia in the CATIE trial
Author(s): Penn, David L.; Keefe, Richard S. E.; Davis, Sonia M.; et al.

Source: SCHIZOPHRENIA RESEARCH Volume: 115 Issue: 1 Pages: 17-23 DOI: 10.1016/j.schres.2009.08.016 Published: NOV 2009
Times Cited: 13 (from All Databases)
Title: Sensory contributions to impaired prosodic processing in schizophrenia
Author(s): Leitman, DI; Foxe, JJ; Butler, PD; et al.
Conference: Annual Meeting of the Society-for-Biological-Psychiatry Location: New York, NY Date: MAY 29-JUN 01, 2004
Sponsor(s): Soc Biolog Psychiatry
Source: BIOLOGICAL PSYCHIATRY Volume: 58 Issue: 1 Pages: 56-61 DOI: 10.1016/j.biopsych.2005.02.034 Published: JUL 1 2005
Times Cited: 52 (from All Databases)
Title: Baseline neurocognitive deficits in the CATIE schizophrenia trial
Author(s): Keefe, Richard S. E.; Bilder, Robert M.; Harvey, Philip D.; et al.
Conference: 60th Annual Convention of the Society-of-Biological-Psychiatry Location: Atlanta, GA Date: MAY 19-21, 2005
Sponsor(s): Soc Biol Psychiat
Source: NEUROPSYCHOPHARMACOLOGY Volume: 31 Issue: 9 Pages: 2033-2046 DOI: 10.1038/sj.npp.1301072 Published: SEP 2006
Times Cited: 140 (from All Databases)
Title: Impact of family involvement on social cognition training in clinically stable outpatients with schizophrenia - A randomized pilot study
Author(s): Tas, Cumhur; Danaci, Aysen E.; Cubukcuoglu, Zeynep; et al.
Source: PSYCHIATRY RESEARCH Volume: 195 Issue: 1-2 Pages: 32-38 DOI: 10.1016/j.psychres.2011.07.031 Published: JAN 30 2012
Times Cited: 1 (from All Databases)
Title: Facial affect recognition in individuals at clinical high risk for psychosis
Author(s): Addington, Jean; Penn, David; Woods, Scott W.; et al.
Source: BRITISH JOURNAL OF PSYCHIATRY Volume: 192 Issue: 1 Pages: 67-68 DOI: 10.1192/bjp.bp.107.039784 Published: JAN 2008
Times Cited: 35 (from All Databases)
Perception of facial emotions in chronic schizophrenia does not correlate with negative symptoms but correlates with cognitive and motor dysfunction.
H. Silver, N. Shlomo
Schizophr Res. 2001 December 1; 52(3): 265–273.
Title: Neurocognitive, social, and emotional dysfunction in deficit syndrome schizophrenia
Author(s): Horan, WP; Blanchard, JJ
Source: SCHIZOPHRENIA RESEARCH Volume: 65 Issue: 2-3 Pages: 125-137 DOI: 10.1016/S0920-9964(02)00410-3 Published: DEC 15 2003

Times Cited: 35 (from All Databases)
Title: The effect of gender on emotion perception in schizophrenia and bipolar disorder
Author(s): Vaskinn, A.; Sundet, K.; Friis, S.; et al.
Source: ACTA PSYCHIATRICA SCANDINAVICA Volume: 116 Issue: 4 Pages: 263-270 DOI: 10.1111/j.1600-0447.2007.00991.x Published: OCT 2007
Times Cited: 21 (from All Databases)
Title: Social cognition mediates illness-related and cognitive influences on social function in patients with schizophrenia-spectrum disorders
Author(s): Addington, Jean; Girard, Todd A.; Christensen, Bruce K.; et al.
Source: JOURNAL OF PSYCHIATRY & NEUROSCIENCE Volume: 35 Issue: 1 Pages: 49-54 DOI: 10.1503/jpn.080039 Published: JAN 2010
Times Cited: 17 (from All Databases)
Title: Emotion Responsivity, Social Cognition, and Functional Outcome in Schizophrenia
Author(s): Mathews, Jennifer R.; Barch, Deanna M.
Source: JOURNAL OF ABNORMAL PSYCHOLOGY Volume: 119 Issue: 1 Pages: 50-59 DOI: 10.1037/a0017861 Published: FEB 2010
Times Cited: 11 (from All Databases)
Title: Facial emotion perception and fusiform gyrus volume in first episode schizophrenia
Author(s): Pinkham, A; Penn, D; Wangelin, B; et al.
Source: SCHIZOPHRENIA RESEARCH Volume: 79 Issue: 2-3 Pages: 341-343 DOI: 10.1016/j.schres.2005.07.012 Published: NOV 15 2005
Times Cited: 4 (from All Databases)
Title: Facial emotion recognition in patients with schizophrenia and their siblings
Author(s): Erol, Almila; Mete, Levent; Sonmez, Ipek; et al.
Source: NORDIC JOURNAL OF PSYCHIATRY Volume: 64 Issue: 1 Pages: 63-67 DOI: 10.3109/08039480903511399 Published: FEB 2010
Times Cited: 2 (from All Databases)
Title: Social cognition and negative symptoms in psychosis
Author(s): Piskulic, Danijela; Addington, Jean
Source: PSYCHIATRY RESEARCH Volume: 188 Issue: 2 Pages: 283-285 DOI: 10.1016/j.psychres.2011.04.028 Published: JUL 30 2011
Times Cited: 0 (from All Databases

11. Social Cognition Screening Questionnaire（SCSQ）

1. 実用可能性と忍容性

(Practicality for Administration and Tolerability for Patients)

Author and Date	Sample	Notes
Roberts et al. (2011)	30 SCZ	The SCSQ contains five subscales: verbal memory, schematic inference, ToM, metacognition, and hostility bias.

Notes: The task comprises 10 short vignettes presenting an interaction between a fictional character and the study participant. Each vignette was read aloud by the tester.

２. 信頼性

(Test-Retest, Internal Consistency, Inter-rater Reliability as applicable)

Author and Date	Sample	Findings
Kanie et al. (2014)	52 SCZ; 53 HC	Cronbach's alpha for the SCSQ total score, including verbal memory, schematic inference, ToM and metacognition, was 0.72.

３. 有用性

(Floor and Ceiling Effects, Normality of Distributions)

Author and Date	Sample	Findings

(Utility as a Repeated Measure)

※この項目では、自然経過では不変で介入によってより大きく動く尺度が良いと考える。

Author and Date	Sample	Treatment Study? Testing Interval	Findings

４．妥当性

(Relationship to social cognitive measures relative to other abilities and constructs)

※この項目では、他の社会認知機能との関連が強い尺度が良いと考える。

Author and Date	Sample	Findings
Roberts et al. (2011)	30 SCZ	The SCSQ's mentalizing scale correlated with the SFS Interpersonal Communication scale (r = .422, P = .023) and Employment scale (r = .368, P = .049). The MSCEIT correlated with the overall SFS score (r = .405, P = .033), and the IPSAQ Personalizing scale correlated with the SFS Prosocial scale (r = .525, P =.003). Notably, the SCSQ total score, Hinting task, and MSCEIT all exhibited trend level correlations with the SSPA (r's = .307–.356).
Kanie et al. (2014)	52 SCZ; 53 HC	**Table 2.** Between-group comparison of SCSQ and AIHQ subscale scores

	Schizophrenia ($n = 52$)	Normal controls ($n = 53$)	Between-group comparison
SCSQ			
Verbal memory	7.92 (1.13)†	8.64 (0.86)	$Z = -3.57$, $P < 0.001$
Schematic inference	7.54 (1.35)	8.60 (0.91)	$Z = -4.31$, $P < 0.0001$
Theory of mind	6.56 (1.51)	8.43 (1.38)	$Z = -6.08$, $P < 0.0001$
Metacognition	9.22 (0.64)	9.50 (0.53)	$Z = 2.43$, $P < 0.05$
Hostility bias	1.52 (1.09)	0.89 (0.91)	$Z = -3.08$, $P < 0.01$
Total	31.24 (3.47)	35.08 (2.52)	$Z = -5.99$, $P < 0.0001$
AIHQ			
Hostility bias			
Intentional	1.93 (0.41)	2.19 (0.40)	$Z = -3.68$, $P < 0.001$
Ambiguous	1.71 (0.46)	1.59 (0.32)	NS
Accidental	1.30 (0.33)	1.25 (0.20)	NS
Blame score			
Intentional	3.23 (0.75	3.54 (0.55)	$Z = -2.44$, $P < 0.05$
Ambiguous	2.49 (0.71)	2.34 (0.48)	NS
Accidental	2.13 (0.64)	2.09 (0.41)	NS
Aggression bias			
Intentional	1.77 (0.64)	1.82 (0.52)	NS
Ambiguous	1.75 (0.61)	1.69 (0.32)	NS
Accidental	1.61 (0.64)	1.51 (0.40)	NS
Hinting Task	14.02 (3.67)	16.23 (3.07)	$Z = -3.34$, $P < 0.001$

†Mean (SD).
AIHQ, Ambiguous Intentions Hostility Questionnaire; NS, not significant; SCSQ, Social Cognition Screening Questionnaire.

Author and Date	Sample	Findings
Kanie et al. (2014)	52 SCZ; 53 HC	**Table 3.** Spearman's rho between SCSQ scores and other social cognition measures

	Verbal memory	Schematic inference	Theory of mind	Metacognition	Hostility bias	Total
Hinting Task	0.35*	0.25	0.52****	0.13	−0.25	0.48***
BCIS						
Composite	−0.10	0.10	0.22	0.32*	0.19	0.20
AIHQ (ambiguous)						
Hostility	0.12	−0.05	−0.06	0.24	0.34*	0.03
Blame	−0.25	−0.28*	−0.42**	−0.17	0.47***	−0.42**
Aggression	−0.31*	−0.26	−0.45***	−0.02	0.37**	−0.35*

****$P < 0.0001$. ***$P < 0.001$. **$P < 0.01$. *$P < 0.05$.
AIHQ, Ambiguous Intentions Hostility Questionnaire; BCIS, Beck Cognitive Insight Scale; SCSQ, Social Cognition Screening Questionnaire.

Author and Date	Sample	Findings
Hamatani et al. (2016)	18 AN (anorexia nervosa); 18 HC	Scores of the SCSQ in AN and HC groups. There were significant differences in ToM (U=85.50, P=0.012, r=−0.42), metacognition (U=94.00, P=0.023, r=−0.38), and total score of the SCSQ (U=67.50, P=0.003, r=−0.50) between the two groups. On the other hand, there was no significant difference in verbal memory, schematic inference, and hostility bias between them. Analysis

		of covariance with scores of the BDI-II, the STAI-JYZ, and BMI as a covariate showed significant differences in scores of ToM ($F(1, 30)=6.60$, $P=0.015$, $\eta p2=0.16$), metacognition ($F(1, 30)=5.62$, P,0.024, $\eta p2=0.13$), and the total ($F(1, 30)=8.01$, $P=0.008$, $\eta p2=0.17$) between the two groups.
Hagiya et al. (2015)	52 SCZ; 53 HC	Table 3 (below)

Table 3. Correlations between FEST demographic, symptom, neurocognitive and social cognition by group

Measure	Score with the FEST
Schizophrenia subjects	
Education	0.08
JART	0.09
PANSS positive symptoms	0.15
PANSS negative symptoms	0.09
Antipsychotic dose	0.01
Hinting task	0.34*
SCSQ	
Verbal memory	0.40**
Schematic inference	0.09
Theory of mind	0.24
Metacognition	0.27*
Hostility bias	–0.22

(Correlations with Real-World Social Outcomes)

Author and Date	Sample	Findings
Glenthøj et al. (2016)	65 UHR patients and 30 healthy controls	SOFAS: The SANS entered the equation first ($F(1,59)=15.463$, $p < .001$) with an R2 of .208, followed by SCSQ ($F(2,58) = 11.176$, $p < .001$) with an R2 of .278. The model indicated that a higher SOFAS is accounted for by a lower SANS and higher SCSQ. PSP: SANS was the only variable entering the equation ($F(1,59) = 37.982$, $p < .001$) with an R2 of .392. The model indicated that a higher PSP is accounted for by a lower SANS. GF: Social. SANS was the only variable entering the equation F(1,59) = 27.927, $p < .001$) with an R2 of .321. The model indicated that a higher GF:Social is accounted for by a lower SANS. GF: Role. The SANS entered the equation first ($F(1,59) = 20.160$, $p < .001$) with an R2 of .225, followed by emotion

		recognition of disgust (F(2,58)=16.123, $p < .001$) with an R2 of .357. The model indicated that a higher GF:Role is accounted for by a lower SANS and lower ERT disgust recognition.
Kanie et al. (2014)	52 SCZ; 53 HC	Table 4. Spearman's rho between SCSQ and SFS subscale scores (see below)

Table 4. Spearman's rho between SCSQ and SFS subscale scores

	Verbal memory	Schematic inference	Theory of mind	Metacognition	Hostility bias	Total
SFS	0.03	0.10	0.23	0.08	–0.22	0.11
Total score						
Social engagement	0.00	0.10	0.33*	0.16	–0.29*	0.27
Interpersonal communication	0.14	–0.01	0.41**	0.18	–0.18	0.26
Independence-performance	–0.09	0.02	–0.04	–0.13	–0.08	–0.12
Recreation	–0.01	0.01	0.38**	0.30*	–0.13	0.22
Social activities	0.12	0.18	0.15	0.08	–0.22	0.16
Independence-competence	–0.08	0.02	0.01	–0.12	–0.11	–0.12
Occupation	0.17	0.04	0.46***	0.29*	–0.22	0.32*

***$P < 0.001$. **$P < 0.01$. *$P < 0.05$.
SCSQ, Social Cognition Screening Questionnaire; SFS, Social Functioning Scale.

文献

ORIGINAL CITATION:

Title: ECOLOGICAL VALIDITY OF THE SOCIAL COGNITION SCREENING QUESTIONNAIRE (SCSQ)

Author(s): Roberts DL, Fiszdon J, Tek C

Conference: 13th International Congress on Schizophrenia Research (ICSR), Colorado Springs, CO, APR 02-06, 2011

Sponsor(s): SCHIZOPHRENIA BULLETIN

Source: Schizophr. Bull. (2011) 37(Suppl. 1): 280

Times Cited: 18 (from All Databases)

CITING ARITCLES

Title: Social cognition in patients at ultra-high risk for psychosis: What is the relation to social skills and functioning?

Author(s): LB Glenthøj, B Fagerlund, C Hjorthøj et al.

Source: Schizophrenia Research: Cognition.Volume 5, September 2016, Pages 21-27

Times Cited: 29 (from All Databases)

Title: New instrument for measuring multiple domains of social cognition: Construct validity of the Social Cognition Screening Questionnaire (Japanese version)

Author(s): A Kanie, K Hagiya, S Ashida, S Pu et al.

Source: Psychiatry Clin Neurosci. 2014 Sep;68(9):701-11.

Times Cited: 21 (from All Databases)

Title: Social cognition and prefrontal hemodynamic responses during a working memory task in schizophrenia

Author(s): S Pu, K Nakagome, T Yamada, M Itakura et al.

Source: Scientific Reports volume 6, Article number: 22500 (2016)

Times Cited: 12 (from All Databases)

Title: Impaired social cognition in anorexia nervosa patients

Author(s): S Hamatani, M Tomotake, T Takeda et al.
Source: Neuropsychiatr Dis Treat. 2016; 12: 2527–2531.
Times Cited: 12 (from All Databases)
Title: An Alternative to Generating Alternative Interpretations in Social Cognitive Therapy for Psychosis
Author(s): DL Roberts, P Kleinlein, B Stevens.
Source: Behavioural and Cognitive Psychotherapy Volume 40, Issue 4 July 2012 , pp. 491-495
Times Cited: 12 (from All Databases)
Title: Facial expression perception correlates with verbal working memory function in schizophrenia
Author(s): K Hagiya, T Sumiyoshi, A Kanie, S Pu et al.
Source: Psychiatry and Clinical Neurosciences2015;69: 773–781
Times Cited: 8 (from All Databases)
Title: Association of fronto-temporal function with cognitive ability in schizophrenia
Author(s): S Pu, K Nakagome, M Itakura, M Iwata, I Nagata et al.
Source: Scientific Reports volume 7, Article number: 42858 (2017)
Times Cited: 5 (from All Databases)
Title: Influence of cognitive function on quality of life in anorexia nervosa patients
Author(s): S Hamatani, M Tomotake, T Takeda et al.
Source: Psychiatry and Clinical Neurosciences2017;71: 328–335
Times Cited: 2 (from All Databases)
Title: Effect of cognitive function on jumping to conclusion in patients with schizophrenia
Author(s): T Takeda, M Nakataki, M Ohta, S Hamatani et al.
Source: Schizophrenia Research: Cognition Volume 12, June 2018, Pages 50-55
Times Cited: 1 (from All Databases)
Title: Neurobiology and treatment of social cognition in schizophrenia: Bridging the bed-bench gap
Author(s): S Kimoto, M Makinodan, T Kishimoto et al.
Source: Neurobiology of Disease Volume 131, November 2019, 104315
Times Cited: 2 (from All Databases)
Title: The Feasibility and Efficacy of Social Cognition and Interaction Training for Outpatients With Schizophrenia in Japan: A Multicenter Randomized Clinical Trial
Author(s): A Kanie, A Kikuchi, D Haga, Y Tanaka, A Ishida et al.
Source: Front. Psychiatry, 23 August 2019
Times Cited: 0 (from All Databases)
Title: Hostile attribution bias in schizophrenia-spectrum disorders: narrative review of the literature and persisting questions
Author(s): B Buck, J Browne, EC Gagen, DL Penn et al.
Source: Journal of Mental Health
Times Cited: 0 (from All Databases)
Title: A meta-analysis of the associations between theory of mind and neurocognition in schizophrenia

Author(s): É Thibaudeau, AM Achim, C Parent, M Turcotte et al.

Source: Schizophrenia Research Available online 31 December 2019

Times Cited: 0 (from All Databases)

Title: Basic symptoms influence real-life functioning and symptoms in individuals at high risk for psychosis

Author(s): LB Glenthøj, B Bailey, TD Kristensen et al.

Source: Acta Psychiatr Scand 2020: 141: 231–240

Times Cited: 0 (from All Databases)

Title: Predictors of remission from the ultra-high risk state for psychosis

Author(s): LB Glenthøj, TD Kristensen et al.

Source: Early Intervention 2020

Times Cited: 0 (from All Databases)

Title: A Feasibility and Acceptability Trial of Social Cognitive Therapy in Early Psychosis Delivered Through a Virtual World: The VEEP Study

Author(s): A Thompson, F Elahi, A Realpe, M Birchwood et al.

Source: Front. Psychiatry, 25 March 2020

Times Cited: 0 (from All Databases)

12. Metaphor and Sarcasm Scenario Test（MSST）

1. 実用可能性と忍容性

(Practicality for Administration and Tolerability for Patients)

Author and Date	Sample	Notes
Adachi, T. et al. (2004)	199 normal school children (the control group), 29 AD/HD children and 54 HFPDD children	This test consists of five metaphoric and sarcastic scenarios. The test employed a multiple-choice style. The number of correct answers in the metaphoric and the sarcastic scenarios represent the metaphoric score and sarcastic score, respectively. One of the incorrect answers in each sarcastic scenario was a ‘landmine answer’. The landmine answer involves misunderstanding the speaker’s sarcasm to mean admiration.
Notes: This is a paper and pencil test. Probably takes no more than 5 minutes.		

２. 信頼性

(Test-Retest, Internal Consistency, Inter-rater Reliability as applicable)

Author and Date	Sample	Findings

３. 有用性

(Floor and Ceiling Effects, Normality of Distributions)

Author and Date	Sample	Findings
Adachi, T. et al. (2004)	199 normal school children (the control group), 29 AD/HD children and 54 HFPDD children	Controls: total 7.4(2.5), metaphoric 4.1(1.2), sarcastic 3.3(1.7), Landmine avoidance 4.2(1.2)<br>AD/HD: total 5.5(2.7), metaphoric 2.5(1.7), sarcastic 3.0(1.6), Landmine avoidance 3.7(1.6)<br>HFPDD: total 4.3(2.8), metaphoric 2.5(1.6), sarcastic 1.8(1.8), Landmine avoidance 2.5(1.7)
Fukuhara, K. et al. (2017)	34patients with schizophrenia and 34 normal subjects	Controls: total 9.24(1.59), metaphoric 5.00(0.00), sarcastic 4.24(1.59), Landmine avoidance 4.62(1.11)<br>Schizophrenia: total 7.44(2.10), metaphoric 4.47(0.88), sarcastic 2.97(1.72), Landmine avoidance 3.88(1.57)

Maki, Y., Yamaguchi, T., Koeda, T., & Yamaguchi, H. (2013)	31 young normal controls（YNC）, 104 aged normal controls (ANC), 42 patients with amnesic mild cognitive impairment (aMCI), and 30 patients with mild AD（AD）	YNC：metaphoric 5.0(0.2), sarcastic 4.8(0.4), Landmine avoidance 0.19(0.40)<br>ANC: metaphoric 4.8(0.7), sarcastic 4.1(1.2), Landmine avoidance 0.19(0.44)<br>aMCI: metaphoric 4.3(1.2), sarcastic 3.4(1.3), Landmine avoidance 0.48(0.77)<br>AD: metaphoric 3.3(1.2), sarcastic 2.3(1.6), Landmine avoidance 1.77(1.72)

(Utility as a Repeated Measure)

※この項目では、自然経過では不変で介入によってより大きく動く尺度が良いと考える。

Author and Date	Sample	Treatment Study?<br>Testing Interval	Findings

４．妥当性

(Relationship to social cognitive measures relative to other abilities and constructs)

※この項目では、他の社会認知機能との関連が強い尺度が良いと考える。

Author and Date	Sample	Findings

<table>
<tr>
<td>Adachi, T. et al. (2004)</td>
<td>199 normal school children (the control group), 29 AD/HD children and 54 HFPDD children</td>
<td>
Correlation of MSST scores with age and IQ
<table>
<tr><th>Groups</th><th>MSST scores</th><th></th><th></th></tr>
<tr><th></th><th>Metaphoric</th><th>Sarcastic</th><th>Landmine avoidance</th></tr>
<tr><td>Controls</td><td></td><td></td><td></td></tr>
<tr><td>Age</td><td>0.576***</td><td>0.269***</td><td>0.095</td></tr>
<tr><td>AD/HDs</td><td></td><td></td><td></td></tr>
<tr><td>Age</td><td>0.326</td><td>0.142</td><td>0.049</td></tr>
<tr><td>FIQ</td><td>0.419*</td><td>−0.040</td><td>−0.086</td></tr>
<tr><td>VIQ</td><td>0.406*</td><td>0.077</td><td>−0.051</td></tr>
<tr><td>PIQ</td><td>0.406*</td><td>−0.050</td><td>−0.042</td></tr>
<tr><td>HFPDDs</td><td></td><td></td><td></td></tr>
<tr><td>Age</td><td>0.481***</td><td>0.220</td><td>−0.047</td></tr>
<tr><td>FIQ</td><td>0.422**</td><td>−0.052</td><td>0.067</td></tr>
<tr><td>VIQ</td><td>0.527***</td><td>0.084</td><td>0.018</td></tr>
<tr><td>PIQ</td><td>0.188</td><td>−0.179</td><td>0.019</td></tr>
</table>
*$P < 0.05$, **$P < 0.01$, ***$P < 0.001$.
</td>
</tr>
<tr>
<td>Fukuhara, K. et al. (2017)</td>
<td>34patients with schizophrenia and 34 normal subjects</td>
<td>In the schizophrenia group, the metaphor score was significantly and inversely correlated with the sum of normal item scores (r=− 0.45, p < 0.05). The sarcasm score was significantly correlated with the sum of abnormal item scores(r=0.40, p < 0.05).
※the Dewey Story Test (DST)におけるnormal item scores、abnormal item scoresとの相関</td>
</tr>
<tr>
<td>Maki, Y., Yamaguchi, T., Koeda, T., & Yamaguchi, H. (2013)</td>
<td>31 young normal controls（YNC）, 104 aged normal controls (ANC), 42 patients with amnesic mild cognitive impairment (aMCI), and 30 patients with mild AD（AD）</td>
<td>There was weak correlation between MMSE scores and metaphor (r¼.362 P < .001) and sarcasm scores (r¼.337, P < .001).</td>
</tr>
</table>

(Correlations with Real-World Social Outcomes)

Author and Date	Sample	Findings

文献

ORIGINAL CITATION:

Title: The metaphor and sarcasm scenario test: A new instrument to help differentiate high functioning pervasive developmental disorder from attention deficit/hyperactivity disorder.

Author(s): Adachi, T., Koeda, T., Hirabayashi, S., Maeoka, Y., Shiota, M., Wright, E. C., et al. (2004).

Source:Brain and Development, 26, 301–306. doi:10.1016/S0387-7604(03)00170-0.

Times Cited: 92 (from Google scholar)

CITING ARITCLES

Title: Impaired interpretation of others' behavior is associated with difficulties in recognizing pragmatic language in patients with schizophrenia.

Author(s):Fukuhara, K., Ogawa, Y., Tanaka, H., Nagata, Y., Nishida, S., Haga, D., & Nishikawa, T. (2017).

Source:Journal of psycholinguistic research, 46(5), 1309-1318.

Times Cited: 3 (from Google scholar)

Title: Communicative competence in Alzheimer's disease: metaphor and sarcasm comprehension. American

Author(s):Maki, Y., Yamaguchi, T., Koeda, T., & Yamaguchi, H. (2013).

Source:Journal of Alzheimer's Disease & Other Dementias®, 28(1), 69-74.

Times Cited: 42 (from Google scholar)

13. Noh Mask Test

1. 実用可能性と忍容性

(Practicality for Administration and Tolerability for Patients)

Author and Date	Sample	Notes
Minoshita, S., Satoh, S., Morita, N., Tagawa, A., & Kikuchi, T. (1999).	15 normal subjects (mean age: 32years, SD 9.7years)	12 tasks, and each task consisted of 15 trials. colour monitor.<br>The subject pressed either the yes or no key.<br>The total time was 30min even in normal individuals.<br>The affirmation rates of each Noh mask image and the mean reaction times to each image for all emotion items were evaluated.<br>The affirmation rates for each emotion item through the Noh mask images and the reaction times for each emotion item through the Noh mask images were evaluated
Minoshita, S., Morita, N., Yamashita, T., Yoshikawa, M., Kikuchi, T., & Satoh, S. (2005)	Fifteen men with schizophrenia and 15 normal controls	Completion of all trials took approximately 30 min for individuals in the control group and approximately 40 min for individuals in the patient group.

２. 信頼性

(Test-Retest, Internal Consistency, Inter-rater Reliability as applicable)

Author and Date	Sample	Findings

３. 有用性

(Floor and Ceiling Effects, Normality of Distributions)

Author and Date	Sample	Findings
Minoshita, S., Satoh, S., Morita, N., Tagawa, A., &	15 normal subjects (mean age: 32years, SD 9.7years)	The image affirmation rate (mean 41.1%, SD 4.9%)<br>The image reaction time (mean 1598.1ms, SD 67.1ms)<br>The emotion item affirmation rate (mean 39.9%, SD 11.0%)<br>The emotion item reaction time (mean 1598.1ms, SD 139.7ms)

Kikuchi, T. (1999).		
Koelkebeck, K., Minoshita, S. et al. (2018)	32 patients with schizophrenia, 32 patients with an MDD and 32 healthy residents	<u>Schizophrenia</u><br>The image affirmation rate (mean 25.1%)<br>The image reaction time (mean 2210ms, SD 1468ms)<br>The emotion item affirmation rate<br>Basic(mean 24.6%)<br>Subtle(mean 25.5%)<br>The emotion item reaction time<br>Basic(mean 2069ms, SD 1466ms)<br>Subtle(mean 2287ms, SD 2287ms)<br><u>MDD</u><br>The image affirmation rate (mean 28.9%)<br>The image reaction time (mean 1962ms, SD 1155ms)<br>The emotion item affirmation rate<br>Basic(mean 26.6%)<br>Subtle(mean 29.3%)<br>The emotion item reaction time<br>Basic(mean 1979ms, SD 1153ms)<br>Subtle(mean 2090ms, SD 1084ms)<br><u>HC</u><br>The image affirmation rate (mean 28.3%)<br>The image reaction time (mean 2046ms, SD 1113ms)<br>The emotion item affirmation rate<br>Basic(mean 27.5%)<br>Subtle(mean 29.7%)<br>The emotion item reaction time<br>Basic(mean 1830ms, SD 1112ms)<br>Subtle(mean 2050ms, SD 1175ms)

(Utility as a Repeated Measure)

※この項目では、自然経過では不変で介入によってより大きく動く尺度が良いと考える。

Author and Date	Sample	Treatment Study? Testing Interval	Findings

４．妥当性

(Relationship to social cognitive measures relative to other abilities and constructs)

※この項目では、他の社会認知機能との関連が強い尺度が良いと考える。

Author and Date	Sample	Findings

(Correlations with Real-World Social Outcomes)

Author and Date	Sample	Findings

文献

ORIGINAL CITATION:

Title: The Noh mask test for analysis of recognition of facial expression.

Author(s): Minoshita, S., Satoh, S., Morita, N., Tagawa, A., & Kikuchi, T. (1999).

Source: Psychiatry and clinical neurosciences, 53(1), 83-89.

Times Cited: 21 (from Google scholar)

CITING ARITCLES

Title: Masked ambiguity–Emotion identification in schizophrenia and major depressive disorder.

Author(s): Koelkebeck, K., Vosseler, A., Kohl, W., Fasshauer, T., Lencer, R., Satoh, S., ... & Minoshita, S. (2018).

Source: Psychiatry research, 270, 852-860.Source:Journal of psycholinguistic research, 46(5), 1309-1318.

Times Cited: 1 (from Google scholar)

Title: Recognition of affect in facial expression using the Noh Mask Test: Comparison of individuals with schizophrenia and normal controls.

Author(s): Minoshita, S., Morita, N., Yamashita, T., Yoshikawa, M., Kikuchi, T., & Satoh, S. (2005).

Source:Psychiatry and clinical neurosciences, 59(1), 4-10.

Times Cited: 23 (from Google scholar)

14. The situational feature recognition test（SFRT）

1. 実用可能性と忍容性

(Practicality for Administration and Tolerability for Patients)

Author and Date	Sample	Notes
Corrigan, P. W., & Green, M. F. (1993)	25 schizophrenic patients<br>15 normal controls	Four test situations were used. multiple-choice.
Notes: This is a paper and pencil test. Probably takes 10-20 minutes.		

２. 信頼性

(Test-Retest, Internal Consistency, Inter-rater Reliability as applicable)

Author and Date	Sample	Findings
Corrigan, P. W., & Green, M. F. (1993)	68 HC	KR-20= 0.95(Target items, Actions), 0.99(Target items, Roles), 0.97(Target items, Rules), 0.98(Target items, Goals), 0.99(Non-target items, Actions), 0.81(Non-target items, Roles), 0.95(Non-target items, Rules), 0.96(Non-target items,Goals)
Washburn, A. M., Sands, L. P., & Walton, P. J. (2003)	Forty nursing home residents with and without cognitive impairment	Cronbach’s alpha=.92<br>Test-retest(1-2 week)=.86

３. 有用性

(Floor and Ceiling Effects, Normality of Distributions)

Author and Date	Sample	Findings
Corrigan, P. W., & Green, M. F. (1993)	68 HC	Target items, Actions:92.6%(4.6%),<br>Target items Roles: 92.0(7.1),<br>Target items, Rules:93.7(4.9),<br>Target items, Goals91.3(7.5)<br>Non-target items, Actions:2.3%(4.4%)<br>Non-target items, Roles: 3.8(2.3)<br>Non-target items, Rules:2.9(4.0)

<table>
<tr><td></td><td></td><td>Non-target items, Goals:2.8(0.96)</td></tr>
<tr><td>Corrigan, P. W., & Green, M. F. (1993)</td><td>25 schizophrenic patients
15 normal controls</td><td>Mean and standard deviations of the frequency of schizophrenic and normal subjects recognition of situational features
<table>
<tr><th>Feature</th><th>Normal subjects</th><th>Schizophrenic subjects</th></tr>
<tr><td>Sensitivity, actions</td><td>0.968 (0.054)</td><td>0.901 (0.147)</td></tr>
<tr><td>Sensitivity, roles</td><td>0.980 (0.023)</td><td>0.879 (0.117)</td></tr>
<tr><td>Sensitivity, rules</td><td>0.980 (0.016)</td><td>0.880 (0.118)</td></tr>
<tr><td>Sensitivity, goals</td><td>0.981 (0.021)</td><td>0.845 (0.134)</td></tr>
<tr><td>Hits, actions</td><td>93.8% (10.3%)</td><td>84.8% (15.0%)</td></tr>
<tr><td>Hits, roles</td><td>90.6 (12.7)</td><td>80.5 (18.6)</td></tr>
<tr><td>Hits, rules</td><td>89.4 (25.1)</td><td>81.2 (18.1)</td></tr>
<tr><td>Hits, goals</td><td>89.7 (25.2)</td><td>77.1 (22.3)</td></tr>
<tr><td>False positives, actions</td><td>4.2% (6.4%)</td><td>13.8% (19.1%)</td></tr>
<tr><td>False positives, roles</td><td>0.1 (1.4)</td><td>18.8 (26.2)</td></tr>
<tr><td>False positives, rules</td><td>3.3 (3.6)</td><td>17.4 (17.5)</td></tr>
<tr><td>False positives, goals</td><td>3.1 (4.1)</td><td>22.0 (20.3)</td></tr>
</table>
Standard deviations given in parentheses.</td></tr>
<tr><td>Breitborde, N. Bell, E. K. et al. (2018)</td><td>71 individuals with first-episode psychosis</td><td>Familiar actions mean=0.91%, SD=0.08%
Familiar goals mean=0.92%, SD=0.10%
Unfamiliar actions mean=0.92%, SD=0.11%
Unfamiliar goals mean=0.90%, SD=0.12%</td></tr>
<tr><td>Washburn, A. M., Sands, L. P., & Walton, P. J. (2003)</td><td>Forty nursing home residents with and without cognitive impairment</td><td>mean=13.6(Possible Range:0-16), SD=2.09</td></tr>
<tr><td>Subotnik, K. L., Nguyen, A. T. et al (2006)</td><td>47 stable outpatients with schizophrenia</td><td>Sensitivity Rules: mean=.91, SD=.06
Sensitivity items, Rules: mean=.93, SD=.03
Sensitivity, Actions: mean=.93, SD=.03
Sensitivity, Goals: mean=.92, SD=.04</td></tr>
<tr><td>Corrigan, P. W., Garman, A., & Nelson, D. (1996)</td><td>31 patients with schizophrenia
Outpatient with schizophrenia 39 normal controls</td><td><u>Inpatients</u>
Sensitivity, Actions: mean=.901, SD=.015
Sensitivity Roles: mean=.879, SD=.12
Sensitivity items, Rules: mean=.880, SD=.12
Sensitivity, Goals: mean=.823, SD=.14
<u>outpatients</u></td></tr>
</table>

		Sensitivity, Actions: mean=.942, SD=.06<br>Sensitivity Roles: mean=.933, SD=.08<br>Sensitivity items, Rules: mean=.914, SD=.09<br>Sensitivity, Goals: mean=.908, SD=.09<br>Normal subjects<br>Sensitivity, Actions: mean=.964, SD=.03<br>Sensitivity Roles: mean=.966, SD=.03<br>Sensitivity items, Rules: mean=.958, SD=.04<br>Sensitivity, Goals: mean=.938, SD=.04
Washburn, A. M., & Sands, L. P. (2006)	15 nursing home residents with cognitive impairment and 25 withoutcognitive impairment	Unimpaired<br>mean=14.2(Possible Range:0-16), SD=2.01<br>Cognitively Impaired<br>mean=12.8(Possible Range:0-16), SD=1.96

(Utility as a Repeated Measure)

※この項目では、自然経過では不変で介入によってより大きく動く尺度が良いと考える。

Author and Date	Sample	Treatment Study?<br>Testing Interval	Findings
Breitborde, N. Bell, E. K. et al. (2018)	71 individuals with first-episode psychosis	Yes<br>6 month	Baseline<br>Familiar actions mean=0.91%, SD=0.08%<br>Familiar goals mean=0.92%, SD=0.10%<br>Unfamiliar actions mean=0.92%, SD=0.11%<br>Unfamiliar goals mean=0.90%, SD=0.12%<br>6 month assesment<br>Familiar actions mean0.92%, SD=0.06%, Cohens'd=0.10<br>Familiar goals 0.94%, SD=0.05%, Cohens'd=0.20<br>Unfamiliar actions 0.92%, SD=0.09%, Cohens'd<0.01<br>Unfamiliar goals mean=0.92%, SD=0.07%, Cohens'd=0.10
Addington, J., Saeedi, H., & Addington, D.	50 people with first-episode psychosis, 53 people with	No<br>1 year	Paired t-tests were used to assess change over time. There was no significant change in any of the social cognition measures for either the

(2006)	people with multi-episode schizophrenia and 55 people as controls		first-episode group or the multi-episode group over time. There was a significant improvement for the control group(SFRT (concrete): t=-4.04, P<0.0005)
Chen, Y., Norton, D., McBain, R., Ongur, D., & Heckers, S. (2009)	29 schizophrenia patients and 27 normal controls		Schizophrenia<br>mean=0.86 SD=0.08<br>Normal Control<br>mean=0.90 SD=0.04

４．妥当性

(Relationship to social cognitive measures relative to other abilities and constructs)

※この項目では、他の社会認知機能との関連が強い尺度が良いと考える。

Author and Date	Sample	Findings
Chen, Y., Norton, D., McBain, R., Ongur, D., & Heckers, S. (2009)	29 schizophrenia patients and 27 normal controls	In controls, SFRT were significantly correlated with performance in the face detection task (r=0.64).<br>In patients, SFRT were not correlated with performance in the face detection task and face discrimination task.

(Correlations with Real-World Social Outcomes)

Author and Date	Sample	Findings
Washburn, A. M., Sands, L. P., & Walton, P. J. (2003)	Forty nursing home residents with and without cognitive impairment	CAMCOG Executive function(r=.50, p<0.01)
Woolverton, C. B., Breitborde, N. J. et al (2018)	71 individuals with first-episode psychosis	There were no statistically significant associations between domains of social knowledge and domains of social functioning.

文献

ORIGINAL CITATION:
Title: The Situational Feature Recognition Test: A measure of schema comprehension for schizophrenia.
Author(s): Corrigan, P. W., & Green, M. F. (1993).
Source: International Journal of Methods in Psychiatric Research, 3(1), 29–35.
Times Cited: 47 (from Google scholar)
CITING ARITCLES
Title: An uncontrolled trial of multi-component care for first-episode psychosis: Effects on social cognition.
Author(s): Breitborde, N. J., Moe, A. M., Woolverton, C., Harrison-Monroe, P., & Bell, E. K. (2018).
Source: Early intervention in psychiatry, 12(3), 464-468.
Times Cited: 4 (from Google scholar)
Title: Assessment of Social Cognition in Frail Older Adults and Its Association With Social Functioning in the Nursing Home.
Author(s): Washburn, A. M., Sands, L. P., & Walton, P. J. (2003).
Source:The Gerontologist, 43(2), 203-212.Source:Psychiatry and clinical neurosciences, 59(1), 4-10.
Times Cited: 32 (from Google scholar)
Title: Influence of social perception and social knowledge on cognitive and social functioning in early psychosis.
Author(s): Addington, J., Saeedi, H., & Addington, D. (2006).
Source: The British Journal of Psychiatry, 189(4), 373-378.
Times Cited: 215 (from Google scholar)
Title: Neurocognitive and social cognitive correlates of formal thought disorder in schizophrenia patients.
Author(s): Subotnik, K. L., Nuechterlein, K. H., Green, M. F., Horan, W. P., Nienow, T. M., Ventura, J., & Nguyen, A. T. (2006).
Source: Schizophrenia research, 85(1-3), 84-95.
Times Cited: 79 (from Google scholar)
Title: Situational feature recognition in schizophrenic outpatients.
Author(s): Corrigan, P. W., Garman, A., & Nelson, D. (1996).
Source: Psychiatry research, 62(3), 251-257.
Times Cited: 22 (from Google scholar)
Title: Social cognition and the course of social functioning in first-episode psychosis.
Author(s): Woolverton, C. B., Bell, E. K., Moe, A. M., Harrison-Monroe, P., & Breitborde, N. J. (2018).
Source: Early intervention in psychiatry, 12(6), 1151-1156.
Times Cited: 4 (from Google scholar)
Title: Social cognition in nursing home residents with and without cognitive impairment.
Author(s): Washburn, A. M., & Sands, L. P. (2006).

Source: The Journals of Gerontology Series B: Psychological Sciences and Social Sciences, 61(3), P174-P179.
Times Cited: 24 (from Google scholar)
Title: Visual and cognitive processing of face information in schizophrenia: detection, discrimination and working memory.
Author(s): Chen, Y., Norton, D., McBain, R., Ongur, D., & Heckers, S. (2009).
Source: Schizophrenia Research, 107(1), 92-98.
Times Cited: 60 (from Google scholar)

15. Biological Motion Task（BM）

1. 実用可能性と忍容性

(Practicality for Administration and Tolerability for Patients)

今回使用する課題は、北海道大学精神医学教室のグループが作成した課題で、全く同じ課題の先行研究はない。所要時間は 10 分程。

２．信頼性

(Test-Retest, Internal Consistency, Inter-rater Reliability as applicable)

今回使用する課題は、北海道大学精神医学教室のグループが作成した課題で、全く同じ課題の先行研究はない。再検査信頼性は検討されていない。

３．有用性

(Floor and Ceiling Effects, Normality of Distributions)

今回使用する課題は、北海道大学精神医学教室のグループが作成した課題で、全く同じ課題の先行研究はない。床／天井効果は検討されていない。

(Utility as a Repeated Measure)

※この項目では、自然経過では不変で介入によってより大きく動く尺度が良いと考える。

今回使用する課題は、北海道大学精神医学教室のグループが作成した課題で、全く同じ課題の先行研究はない。自然経過による変化も検討されていない。

４．妥当性

(Relationship to social cognitive measures relative to other abilities and constructs)

※この項目では、他の社会認知機能との関連が強い尺度が良いと考える。

Author and Date	Sample	Findings
橋本ら、2011	29 schizophrenia patients and 29 normal controls	Cohen’s d= 0.99

(Correlations with Real-World Social Outcomes)

Author and Date	Sample	Findings
橋本ら、2011	29 schizophrenia patients	弁別ドット数（識別閾値）と、SOFAS との単相関 0.44 (p<0.05)が認められた。

文献

第6回日本統合失調症学会（2011年7月18-19日，札幌） 橋本直樹、久住一郎、豊巻敦人、賀古勇輝、朴秀賢、伊藤侯輝、小山司 「統合失調症患者におけるcoherent motion知覚とbiological motion知覚の関連および臨床背景との相関の検討」
